# Multi-Scale Modeling of Plastic Waste Gasification: Opportunities and Challenges

**DOI:** 10.3390/ma15124215

**Published:** 2022-06-14

**Authors:** Sepehr Madanikashani, Laurien A. Vandewalle, Steven De Meester, Juray De Wilde, Kevin M. Van Geem

**Affiliations:** 1 Laboratory for Chemical Technology (LCT), Faculty of Engineering and Architecture, Ghent University, Technologiepark 125, 9052 Ghent, Belgium; sepehr.madanikashani@ugent.be (S.M.); laurien.vandewalle@ugent.be (L.A.V.); 2 Materials and Process Engineering (IMAP), Institute of Mechanics, Materials and Civil Engineering (iMMC), Université Catholique de Louvain, Place Sainte Barbe 2, 1348 Louvain-la-Neuve, Belgium; juray.dewilde@uclouvain.be; 3 Laboratory for Circular Process Engineering (LCPE), Faculty of Bioscience Engineering, Ghent University, Campus Kortrijk, Graaf Karel de Goedelaan 5, 8500 Kortrijk, Belgium; steven.demeester@ugent.be

**Keywords:** thermochemical recycling, solid plastic waste, gasification, multi-scale modeling, kinetic modeling, transport phenomena, multi-phase flow, computational fluid dynamics

## Abstract

Among the different thermo-chemical recycling routes for plastic waste valorization, gasification is one of the most promising, converting plastic waste into syngas (H_2_+CO) and energy in the presence of an oxygen-rich gas. Plastic waste gasification is associated with many different complexities due to the multi-scale nature of the process, the feedstock complexity (mixed polyolefins with different contaminations), intricate reaction mechanisms, plastic properties (melting behavior and molecular weight distribution), and complex transport phenomena in a multi-phase flow system. Hence, creating a reliable model calls for an extensive understanding of the phenomena at all scales, and more advanced modeling approaches than those applied today are required. Indeed, modeling of plastic waste gasification (PWG) is still in its infancy today. Our review paper shows that the thermophysical properties are rarely properly defined. Challenges in this regard together with possible methodologies to decently define these properties have been elaborated. The complexities regarding the kinetic modeling of gasification are numerous, compared to, e.g., plastic waste pyrolysis, or coal and biomass gasification, which are elaborated in this work along with the possible solutions to overcome them. Moreover, transport limitations and phase transformations, which affect the apparent kinetics of the process, are not usually considered, while it is demonstrated in this review that they are crucial in the robust prediction of the outcome. Hence, possible approaches in implementing available models to consider these limitations are suggested. Finally, the reactor-scale phenomena of PWG, which are more intricate than the similar processes—due to the presence of molten plastic—are usually simplified to the gas-solid systems, which can result in unreliable modeling frameworks. In this regard, an opportunity lies in the increased computational power that helps improve the model’s precision and allows us to include those complexities within the multi-scale PWG modeling. Using the more accurate modeling methodologies in combination with multi-scale modeling approaches will, in a decade, allow us to perform a rigorous optimization of the PWG process, improve existing and develop new gasifiers, and avoid fouling issues caused by tar.

## 1. Introduction

Over the years, technological advances, population increase, and lifestyle changes have increased the amount of municipal solid waste (MSW), including food residue, wood waste, paper, textiles, plastics, and rubber. Although the rate of plastic production increase has been decreasing in recent years [1,2,3], and the world’s plastic production has been decreased by around 0.2% in 2020 compared to 2019 [4], the amount of plastic waste (PW) to be treated is still huge. Failure in the management of this waste results in catastrophic environmental problems. As an example, according to Cordier and Uehara [5], the estimated annual amount of PW entering the oceans is 4.8 MT and the total amount of plastics in the oceans could increase from 79.24 MT in 2010 to 183.14 MT (the worst-case scenario) in 2030. Moreover, the energy required for the production of plastics, similar to many other industrial processes, is associated with a huge amount of CO_2_ emissions, causing another important environmental problem.

**Figure 1:**
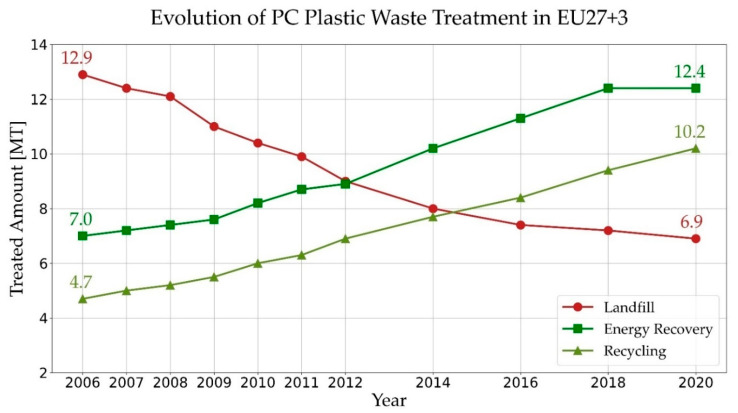
The evolution of the share of treatment methods for the post-consumer (PC) plastic waste in EU member states, Norway, Switzerland, and the United Kingdom (Adapted from Ref. [4]).

To deal with and manage this waste, different treatment methods are used, including landfilling, mechanical recycling, energy recovery, and thermo-chemical recycling. Depending on the location and specific requirements, each of these methods has its benefits and disadvantages. However, landfill sites shortage [6] and global warming are becoming serious deterrents to continuing landfilling and energy recovery, respectively. Hence, in the shift towards a circular economy for plastics, several recycling pathways have been investigated and in some cases commercialized. A higher increase rate of recycling compared to the energy recovery and negative trend of landfilling demonstrates the growing interest in recovering PW (Figure 1).

It is worth mentioning that other than the recycling methods, alternatives can also fulfill reaching the circular economy goal. One of the important alternatives can be the reuse of the plastic waste in the construction industry [7,8], e.g., using PET and/or polyolefinic wastes in concrete, paving, soil-cement blocks, mortar, unfired clay brick, asphalt-concrete mixtures [7], or masonry bricks [9]. Although the use of plastic waste in construction is still limited, it can compensate for the limitations that are faced in the recycling routes of the plastic circular economy. They not only help in reducing the problems associated with plastic wastes, but the construction industry can also benefit from it by decreasing the depletion of natural resources [8].

**Figure 2:**
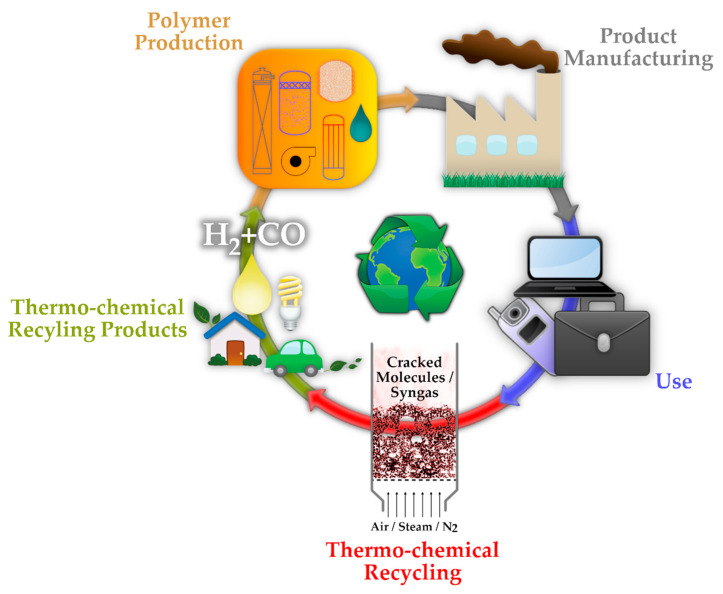
Thermo-chemical recycling path of the plastic circular economy (adapted from [3]).

Mechanical recycling and re-extrusion are currently the most commercialized routes contributing to the current recycling rates of plastics, but they face limitations related to the quality of regranulates. End markets for these recyclates are limited due to uncertainty about their technical properties and other issues such as color and odor [10]. Although some new advancements have been made—such as using the polymer waste in 3D printing [11]—the thermo-chemical recycling pathway is intriguing and promising since it produces, or leads to the production of, a wide variety of products, including syngas, liquid pyrolysis oil, monomers, and petrochemical feedstock [12], as well as energy. Hence, this method is one of the options that can close the loop from PW to plastic production, to achieve a circular economy (Figure 2).

Thermo-chemical recycling encompasses chemolysis, pyrolysis, gasification, fluid catalytic cracking (FCC), hydrogen technologies, and the KDV (Katalytische Drucklose Verölung in German or catalytic pressure-less depolymerization) process, which are explained extensively by Ragaert et al. [12]. Via catalytic routes for thermo-chemical recycling [13], some more unique products, such as carbon nanotube—a nano-scale hollow cylindrical structure—can also be produced. Moreover, the thermo-chemical recycling route can process a mixture of PW streams [14] without the need for substantial pre-treatment steps [15].

**Figure 3:**
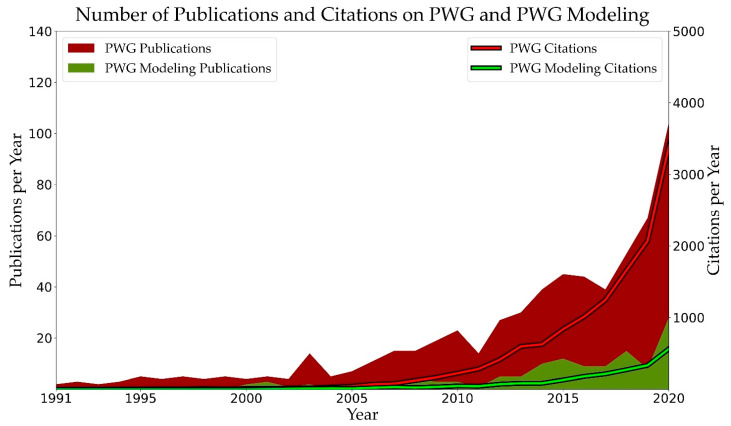
Increased number of publications and citations related to the PWG and modeling of PWG (extracted from [16]).

One of the most promising thermo-chemical recycling routes of PW is gasification, which is defined as a process to convert carbon-containing materials into synthetic/synthesis gas (H_2_ + CO) or so-called syngas, and is conducted at high temperatures (e.g., 850 °C) and usually at atmospheric pressure. This fact can be understood from the strong increase in the number of publications and citations in the field of plastic waste gasification (PWG) and its modeling (Figure 3). Considering this trend and the extensive efforts that should be made to solve the problem of plastic waste, the goal of this review is to assess the opportunities and challenges of PWG from the viewpoint of multi-scale modeling. To do so, first, the process and its multi-scale modeling perspective in this review should be understood and clarified, which is summarized in Section 2. Afterward, opportunities and challenges in modeling this process on different scales, from molecular to the reactor, are reviewed, which span modeling the: thermophysical properties, reaction kinetics, internal and external transport phenomena together with phase transformations, and multi-phase flow modeling.

## 2. Plastic Waste Gasification: A Promising, but Less Mature Recycling Route

**Figure 4:**
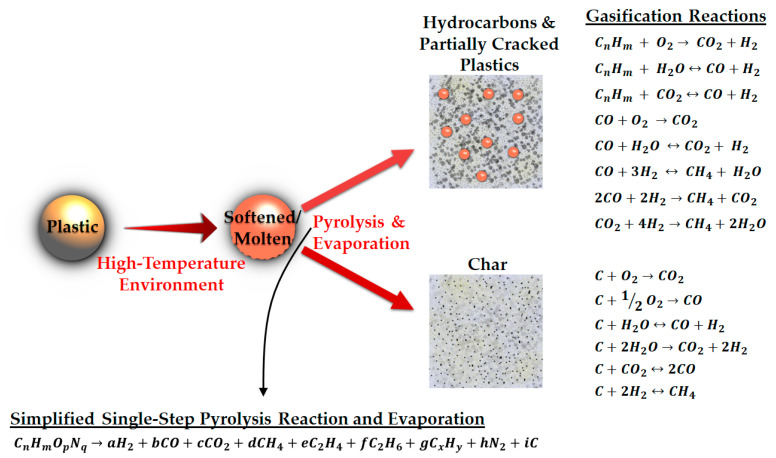
An overview of the plastic gasification process and the reactions taking place (the equations are taken from [20,22,23,24,25]). The separation of the hydrocarbons/partially cracked plastics and char is for the sake of illustration. Otherwise, they are present simultaneously in the reactor.

Figure 4 demonstrates a simplified schematic of sequences occurring during gasification. PWG starts with melting of the PW, which is followed by pyrolysis, evaporation, and gasification. In pyrolysis, the cracking of the molecules in the liquid phase occurs. Some of the lighter species that are produced during this step are transferred to the gas phase (evaporation) [17], while the molten phase contains the depolymerized intermediates, including oils and waxes, cyclic compounds, diesel, and naphtha [18]. Subsequently, gas-phase pyrolysis and/or gasification reactions occur. Moreover, the produced char takes part in the gasification reactions. Typically gasification agents such as air or a mixture of air and steam are present in the reactor, depending on the desired product distribution and heating value [19] and the available separation technologies downstream.

### 2.1. Opportunities and Challenges of PWG

The opportunity that PWG offers is having more flexibility in processing different mixtures of plastic types, together with other feedstock, such as coal and biomass [19], which has made it a stronger thermo-chemical recycling route compared to other methods [20]. This can be attributed to the higher temperature environment, which results in smaller and more stable components. Another advantage is that this process is usually conducted without a catalyst, eliminating concerns about deactivation by impurities [21]. Although this process is beneficial because of the abovementioned facts, its complexity should not be underestimated, as described below.

First, real-world PW streams show a wide variety of polymer compositions [10], and the process has to deal with changing feedstock composition, which results in non-negligible changes in its product distributions [26,27]. Moreover, the presence of impurities in PW makes the situation more complicated. Hence, substantial upgrading of the syngas is required before being used in downstream processes such as Fischer-Tropsch synthesis (FTS) [28].

Second, the product distribution/properties from the gasification of a mixture of PW cannot be derived as a weighted average of the products from the individual gasification of each type of plastic in the mixture [29]. Understanding how impurities and mixing influence the outcome of PWG could be one of the important challenges toward upscaling PWG.

Third, the operation of the PWG process is complex and different from other gasification technologies, e.g., for coal or biomass, due to the presence of molten plastic [6,12,30]. This is while many of the designs and operations of the PWG are based on experience from coal or biomass gasifiers. This can be inferred from the review articles in the field of gasification (general) or PWG [20,25,31,32,33,34,35,36], in which no (or very small) discussion has been made on the presence of molten plastic and liquid phase. 

### 2.2. Numerical Modeling

Considering the above-mentioned complexities and challenges, to properly understand this process, experimental studies can be carried out. Nevertheless, such experimental approaches are time-consuming and resource-intensive, and scale-up studies using experimental approaches remain not straightforward. Fortunately, a combination of experimental work and a validated numerical framework can be a solution to assess and optimize the performance of new reactor designs and process configurations at a much lower cost. This is valid for all processes in general. However, since the gasification of PW is less mature than of other feedstock, such as coal or biomass, the numerical studies are typically (over)simplified.

One of the numerical approaches to assess this process thoroughly is multi-scale modeling. Multi-scale modeling covers time and length scales that allow us to span from sub-atomic to chemical plants the size of small cities. In principle, all the phenomena even in plant size are happening on sub-atomic scales. However, due to the unfeasibility of modeling the whole process on this scale, some possible solutions can be used to decrease the computational cost. For example, the concept of rate-determining step can be used to decrease the size of reaction kinetic network, continuum description of solids can exclude the necessity of defining each particle in a computational fluid dynamics (CFD) framework, and Reynolds-averaging can decrease the computational cost of the instantaneous definition of turbulent fluctuations within a CFD framework. These solutions, in general, are called scale-bridging strategies that make calculations computationally tractable but introduce closure problems. This means that it is possible to simulate the small-scale phenomena (at a lower resolution and sometimes on larger scales), by creating models that predict the effect of sub-scale phenomena. In fact, multi-scale modeling is a framework in which different phenomena are modeled at different scales and subsequently, they become connected to each other via scale-bridging strategies, which are closure models and correlations.

In the field of PWG, the reactive CFD simulations can be considered as an example of a multi-scale modeling framework, which can include the kinetic model, transport phenomena at the particle scale, and the reactor model that is ultimately implemented for process designs or reactor optimization [37]. To date, many of the numerical modeling and simulation studies poorly address all the aforementioned (and other) complexities.

At the molecular scale, kinetic modeling and simulation of PWG are usually based on a single polymer type and the feedstocks are not described in detail. Hence, for numerically capturing the effect of a more complex feedstock, a more fundamental kinetic model, and hence, a more detailed description of the feed and its properties are needed.

**Figure 5:**
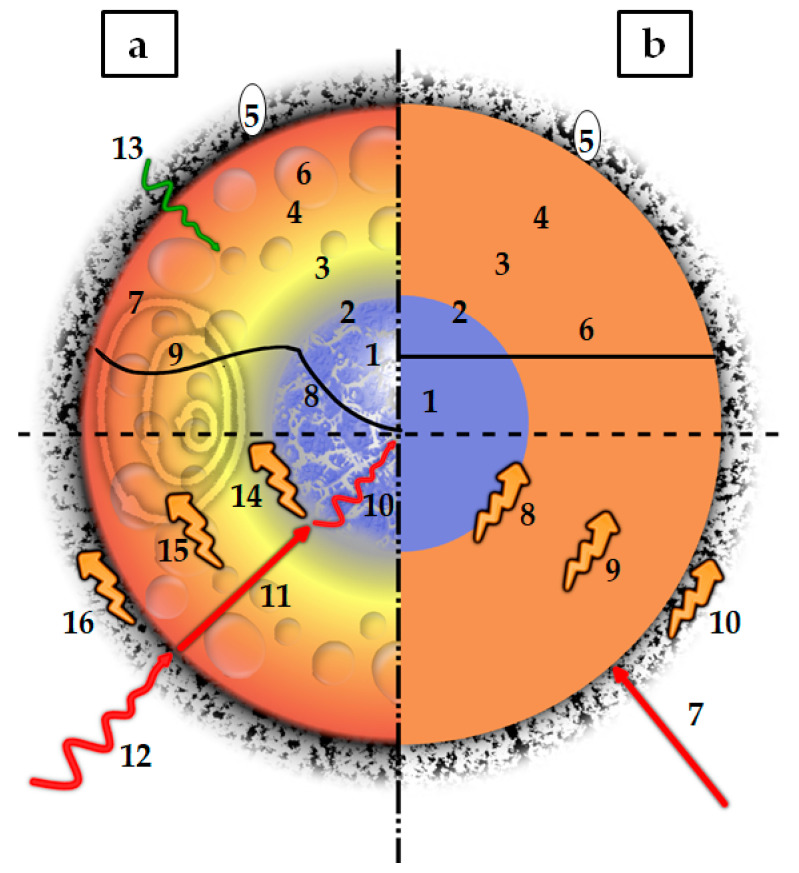
Simplified schematic of the sequential phenomena happening during the solid plastic pyrolysis and gasification (adapted from [38,51,52]); (**a**) Comprehensive modeling approach: (1) Porous solid plastic core; (2) Melt front; (3) Liquid layer; (4) Pyrolysis and evaporation (devolatilization) layer; (5) Gasification layer (including char); (6) Bubbles present in the liquid layer as the result of pyrolysis and evaporation; (7) Vortex-pattern flows as the result of Marangoni and convection effects; (8) Diffusive transport phenomena; (9) Possible temperature (or concentration) profile as the result of internal circulations in the liquid phase; (10) Internal radiative and conductive heat transfer; (11) Conductive and convective heat and mass transfer; (12) Radiation and convective heat and mass transfer; (13) Mass diffusion; (14) Heat of melting; (15) Heat of decomposition and evaporation; (16) Heat of gasification; (**b**) An example of a simplified approach: (1) Solid plastic core; (2) Sharp melt front; (3) Liquid layer; (4) Pyrolysis and evaporation (devolatilization) layer; (5) Gasification layer (including char); (6) Infinite internal heat and mass transfer; (7) Convective heat and mass transfer; (8) Heat of melting; (9) Heat of decomposition and evaporation; (10) Heat of gasification; A detailed description of each part is given throughout this review.

At the particle scale, the definition of gasification is simplified and different phenomena—e.g., melting, evaporation, internal transport limitations—are usually neglected. Figure 5 shows one of the possible routes of PWG in which plastics enter the reaction environment as a solid particle, in a comprehensive approach (a), as well as a simplified one (b). The outer layer of the plastic starts melting due to the external heat transfer. This melt front, which includes a transition mushy zone, moves toward the center while in the melted outer layer, decomposition, pyrolysis, and evaporation occur. Internal transport limitations can affect the rate of transfer of heat and mass to different layers and locations. This can be a reason for non-uniform but shell-progressive melting of the particle [38]. Non-uniform temperature/concentration profiles, as well as the presence of bubbles as the result of evaporation, can also be seen in this figure.

Considering the whole process, reactor scale phenomena are crucial in determining the hydrodynamic behavior and temperature/product distribution of the gasifier. Different types of gasifiers can be used for the PWG, such as fixed bed [39] and moving bed reactors [40]. The most commonly used reactor type for PWG is the fluidized bed (FB) and hence, this reactor type is the main focus of this review. Dual fluidized bed reactors (DFB) are the extension to the conventional FB [26] that can be used in this process. Conical-spouted bed reactors [41,42,43,44] are another type of FB gasifier that offers a wide range of operability from the particle size point of view [20] and for preventing agglomeration and defluidization [45] due to the strong circulation patterns of the solid particles in the reactor. Melting can cause agglomeration of the sticky particles and eventually defluidization [19]. Furthermore, molten particles might stick to the walls or form a layer on them. However, these phenomena are also often neglected. Other technologies can be also used in this process, such as vortex reactor or vortex chamber [46,47], plasma technology [48], entrained flow gasifiers [29], rotary kiln reactor [49], and moving-grate gasifiers [50]. 

The flow pattern in the fluidized bed reactor types can get complex. Simplified reactor models don’t reflect the precise hydrodynamic behavior and temperature profile throughout the reactor, especially the FB reactors, and hence they under- or over-predict the formation of side products. As an example, the residence time and temperature are two important parameters in determining the tar formation and its type [20]. A simplified reactor model can result in an incorrect prediction of tar formation—as the result of inaccurate residence time and temperature distribution prediction. This can lead to fouling issues that are resulted from tar condensation [6] in the reactor and downstream units.

### 2.3. Multi-Scale Modeling of Plastic Waste Gasification

**Figure 6:**
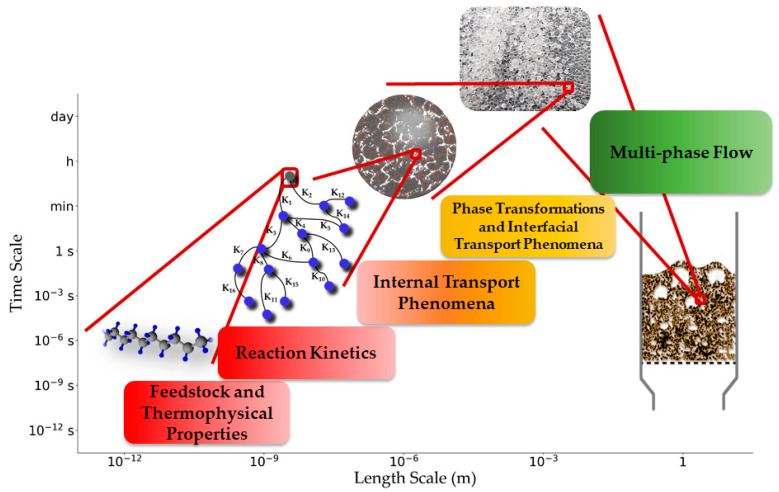
An overview of the multi-scale nature of plastic gasification (adapted from [53,54,55]).

Figure 6 illustrates different scales of a thermo-chemical conversion of a solid feedstock. At the molecular level, modeling the feedstock properties and the chemical reactions is done. Rate of the chemical reactions can be affected by internal transport limitations and interfacial transport phenomena. Hence, the kinetic models are coupled to these transport phenomena and phase transition models, which are usually assessed at the particle scale. Finally, the reactor model accounts for the multi-phase flow and turbulence. To include all or parts of these scales in a multi-scale framework, they have to be connected to each other, which is done by scale-bridging strategies.

In this context, the objective of the following sections is to review modeling approaches for PWG from a multi-scale modeling point of view, which was described above. This allows identifying the challenges and opportunities of PWG in general, but of a model-based design in particular. The starting point of modeling PWG is to describe the properties of the feedstock—discussed in Section 3. In Section 4, the most important phenomena at the molecular scale, i.e., the reactions and kinetic modeling are discussed. Section 5 and Section 6 cover the phenomena that are usually associated with the particle-scale resolution, including internal transport phenomena, phase transformations, and interfacial transport phenomena. In Section 7, the multi-phase flow modeling in the reactor scale using the engineering or computational fluid dynamics (CFD) approaches is assessed. To the best of the authors’ knowledge, few studies are focusing specifically on modeling the PWG process at different scales [29,56,57,58,59,60,61,62,63,64,65]. The analysis that is made during the next sections is used to provide conclusions to bring the first principles-based multi-scale modeling of PWG a step closer. 

Finally, it is worth mentioning that the PWG can be done in different ways, and hence, in this review, four most commonly encountered situations/routes will be considered:

1. The plastic, in the solid phase, is fed into the reactor
2. The plastic is melted first and then fed into the reactor to cover the fluidization agent or to be present as liquid droplets. The latter is reported very rarely [66].
3. The plastic is melted first but fed as a layer into a falling film reactor [67].
4. A bulk of molten plastic is present in the reactor and the gas phase is present as a bubble. This can be found in stirred reactor technologies [68,69].

In this regard, some simplifying assumptions can be of great help in modeling PWG in some of these situations. One of the important ones—which can be observed in most of the available numerical studies for PWG—is considering only gas and solid phases, i.e., similar to biomass or coal gasification. This can be a strong assumption. However, for certain cases, similarities with biomass and coal gasification can be observed and used to adapt modeling approaches developed for these applications. Besides, for the second situation mentioned above, if the operation allows, a thin layer of molten plastic covers the fluidization agents [45], so that the effects of liquid presence can be neglected. Consequently, this case can also be considered a gas-solid-only system. Otherwise, when the plastic is melted in the reactor or a thick layer of molten plastic covers the fluidization agent, the transport phenomena in the liquid and gas phases (ideally) should be considered. In this review, we tried to discuss multi-scale modeling approaches for these types of operations together with their simplifying assumptions. 

## 3. Thermophysical Properties

Determining the initial conditions and thermophysical properties is one of the important challenges in modeling PWG since it can substantially and negatively affect the outcome of even high-fidelity models if they are not defined correctly. This is due to the presence of chain length distribution, additives, particle size distribution, and the presence of a mixture of many different species in all phases.

### 3.1. Individual Species

The first step in determining the thermophysical properties in a model is to choose which species should be included. Defining the species as reactant and product depends on the detail level of the kinetic model that is going to be implemented (Section 4). Afterward, the properties of the individual species that are present in the system should be specified. For the solid phase, only the thermal properties are essential, i.e., the heat capacity, enthalpy, thermal conductivity, and radiation properties, because only heat transfer and melting [70,71,72] are important for this phase. These properties can be determined experimentally or numerically. In the conventional methods, usually, experimental techniques are used to determine the properties, while in the more advanced methods, numerical techniques are of great help.

#### 3.1.1. Conventional Methods

In the conventional (mainly experimental) methods, often a constant value [70] or a temperature-dependent relation [72] is reported. This method is usually used in multi-scale modeling of PWG or similar processes due to its low computational cost. For several reasons, a more robust alternative method to the experimental approach can be the computational methods, which are discussed in the next paragraphs.

First, it has been proven that the accuracy of the experimental values, e.g., heat capacity, can be doubtful. In some cases, significant inconsistencies in the reported values, even for the well-characterized polymers, have been observed [73].

Second, some of the properties are easier to be determined by simulation rather than the experimental methods [74]. As an example, anisotropy in the thermal conductivity of polystyrene has been demonstrated via molecular simulation and this can affect the heat transfer behavior of this polymer.

Third, different studies have shown the dependence of different transport properties of polymers, e.g., viscosity and diffusion, on the chain length because they are functions of the molecules’ arrangements [75]. Also, collision and phonon vibrations affect those values [76]. Hence, especially in the case of PW, it is possible that the available data cannot be used for all the molecules and species that are present in the system, and some experimental-based correlations should be developed for each specific species [77]. Consequently, for the less known and complicated molecules, the computational methods are preferred compared to the classical methods [78], since they give more insight into the molecular structure and are supposed to better predict the behavior of molecules in different conditions.

Determining the thermophysical properties in the liquid and gas phases is supposed to be more challenging, due to the presence of many more different species compared to the solid phase. Similar to the solids, conventional experimental methods can be used to obtain the thermophysical properties of liquid- and gas-phase species. The recent developments of state-of-the-art experimental methods in the field of microfluidics [79]—which can measure the properties at high temperature and pressure—can be an opportunity in this field. Nevertheless, determining the properties of the complex matrix of the PWG process using these methods, may not be less intricate and costly than the numerical methods.

Besides the experimental methods, conventional theoretical approaches can be also used for calculating thermophysical properties, e.g., the standard kinetic theory expressions to derive the transport properties of the gas species [80]. However, the challenge is implementing these conventional methods for the wide range of components that are present in the molten and gas phases. Hence, it can be concluded that, if a model is available to estimate these properties based on the atomic structure and/or molecular properties of the polymer [73], the prediction of the values as the result of a change in the molecule can be easier. Molecular dynamics (MD) methods [75,76] are powerful tools in this regard and are discussed in the following paragraphs.

#### 3.1.2. Advanced Numerical Methods

Conventionally, the role of numerical methods in determining the thermophysical properties was limited to correlating the experimental data [81]. On the contrary, fully theoretical methods, which date back to the 1930s [82], are based on the atomic interactions in the molecules. The opportunity that lies in the molecular and atomic scale simulation approaches, versus the classical methods, is the ability to predict a large number of the thermophysical properties, from vapor pressure to viscosity of molecules at different levels of complexities and a wide range of operating conditions [78]. In general, the ab-initio method [82] and molecular simulations [78] are mainly used for fundamentally deriving the properties of a molecule. As an example, De Tar et al. [82] proposed an ab-initio method using vibrational frequencies, without any tuning, to calculate the heat capacity, enthalpy, and entropy of a series of alkanes. The other properties can be also obtained similarly, the details of which are not in the scope of this review and can be found in the literature [83]. Implementing these methods strongly depends on the available resources and the economy of the problem. The current economy doesn’t allow using ab initio or MD simulations in large scale multi-scale frameworks, e.g., CFD simulations. Hence, the conventional methods are still favorable in these cases.

Using MD methods in predicting transport properties such as the thermal conductivity of polymers [84] becomes interesting, especially for the transition between different phases. According to Henry and Chen [85], polymers are materials with low conductivity. However, a single polyethylene chain can show a high thermal conductivity, as was demonstrated by the MD method. This can demonstrate that the thermal properties of polymers change during their phase transformation [86]. Hence, it can be concluded that numerical models at the molecular levels, if the economy allows, can be used to improve the performance of the multi-scale modeling frameworks where polymer melting and transformation occur. Implementing these models as an integrated part of the multi-scale simulation framework for the whole PWG process is still impossible because of the computational limitations. Nevertheless, they can be used to develop scale-bridging correlations to better predict the thermophysical properties of the materials as a function of larger scale phenomena, thus decreasing computational costs while introducing the closure models.

### 3.2. Effective Properties

In the cases that the intrinsic properties of a species change due to:

1. Structural effects
2. The presence of impurities, or
3. Internal motions (in the liquid phase)

Using the effective properties is a method to include those effects. In the solid phase, these effective properties are considered to correct the transport properties affected by structural characteristics of the solids, i.e., the porosity and tortuosity [87]. Another effective property that can be considered for the plastic while it is still solid is the effective thermal conductivity due to the radiation inside the porous polymer [86]. This has been shown in Figure 5.

The presence of non-native species/phases can also affect the properties. For the materials, such as composites, which are mixtures of different materials including polymers, the effective thermal conductivity can be calculated as a function of the thermal conductivity of different materials that are used in the composite structure [88]. Similarly, for the plastic in the molten phase, the presence of bubbles can impose effective properties. This has not been studied yet for plastics, but the approaches in similar works can be of help in this regard. Sakiyama et al. [89] have assessed the effect of volume fraction of gas bubbles in the food gels on its thermal conductivity and concluded that the effective thermal conductivity for this case is a function of the volume fraction of dispersed and continuous phases.

Besides the presence of impurities or gas bubbles in the liquid phase, the effective properties can be defined if recirculations and vortex flow inside droplets [90] are present. These recirculations are internal motions inside the liquid droplet caused by different factors such as thermocapillary effects [91], which are discussed more in Section 5.

### 3.3. Mixture Properties

After determining the individual species’ properties, they should be assessed in the mixtures that are present in a PWG process. For the solid phase, this is done via simple mass-weighted averaging methods since the mixing of solid plastic is on particle scales and it doesn’t have any effect on their properties at the molecular level. Nevertheless, in liquid and gas phases, the presence of different species affects their individual properties. As an example, in a multi-component gas, if the mixture contains components with a large difference in polarity, the molar average of thermal conductivity is less than the real thermal conductivity value of the mixture. In the opposite case, i.e., for non-polar gas mixtures, the molar average value is larger than the real value and this deviation becomes larger if the molecular weight/size difference of components is large. Similarly, in the case of organic liquids, the thermal conductivity of the mixture is usually less than the average of the components’ thermal conductivity [92]. Hence, a more complex formulation for the multi-component system, beyond the simple averaging methods for the mixtures [80], is required. However, this depends on the required resolution of the simulation framework, as well as the available computational resources. With the current infrastructure, for the large-scale simulation, simplifying assumptions or simple averaging is preferred over complex mixture properties calculations.

In the simulations that are directly or indirectly related to the PWG, the mixing rules are not usually discussed, while for different properties, different mixing rules are available in the literature [92], as are mentioned in Table 1. Even though implementing these mixing rules is very complicated and computationally expensive, they don’t always provide precise results and different mixing rules can result in different results [93]. Hence, to save the computational resources and based on the problem economy, first, it should be determined if using complex mixing rules are beneficial and feasible. MD simulations can also be used in this regard. Ferkl et al. [93] used MD simulations as a reference to determine the best mixing rule for their desired mixture. It can be concluded that although using molecular simulations in the multi-scale platforms may not be feasible yet, they can be used as a validation tool for the simplified models that are applied for calculating the thermophysical properties of the pure components or their mixture.

Finally, it is worth mentioning that in some situations, for the sake of the problem’s economy, calculating the transport properties can be simplified or avoided, such as when the heat diffusion is so fast that the particle or droplet can be considered as isothermal, or in the case that the turbulent viscosity is dominant over the molecular viscosity. Hence, the limiting steps in the transport phenomena can be determined first to avoid the effort and computational costs devoted to determining accurately the transport properties.

## 4. Reaction Kinetics

The reactive part of PWG was demonstrated in Section 2. The kinetic modeling of these reactions is complicated due to various reasons such as the inherent complexity of free-radical chemistry, the chain-length distribution of the polymer molecules [94], presence of the liquid, gas, and solid phases [95], the effect of interaction between different PW types [96], and also the effect of impurities [97], among others. 

The main focus of other review articles on kinetic modeling is the pyrolysis of the neat polymers [32,95,98,99,100]. Although the gasification process includes pyrolysis reactions, it is distinct from the pyrolysis process by three distinguishing factors, which are the temperature range, the presence of gasification agent, and the char gasification reactions, which are discussed more in the following parts.

### 4.1. Challenges Faced in Gasification and/vs. Pyrolysis

In general, kinetic modeling of pyrolysis and gasification of PW faces some challenges, some of which are specific to gasification. In the following sub-sections, these challenges are reviewed.

#### 4.1.1. Diverse Micro-Scale Characteristics of Plastics

**Figure 7:**
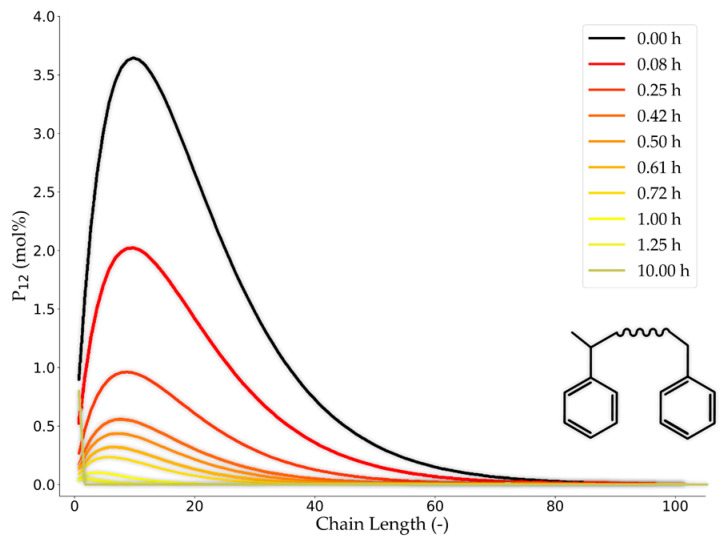
The initial, and transformation of the, chain-length distribution (molar mass distribution) of a type of Poly(styrene peroxide) (P_12_) during the thermal degradation, predicted by a tree-based kinetic Monte Carlo coupled to an artificial intelligence tool. Reprinted (adapted) with permission from [94]. Copyright 2021 American Chemical Society.

One of the reasons that complicates the kinetic modeling of PWG is the large variety in the chain-length distribution (or molar mass distribution) of molecules compared to other free radical processes such as steam cracking, oxidation, or combustion. This means that the kinetic models for PWG need to be huge because they should, in theory, consider all species where the initiation reactions take place. Inevitably this gets even more complex because the chain-length distribution changes while the polymer is being degraded in the pyrolysis reactions (Figure 7). Diverse chain length distribution of polymers from different sources increases these complexities. Consequently, defining a fixed initial condition for the composition and chain length distribution for modeling the gasification of PW is challenging [94]. Other polymer micro-scale characteristics, such as backbone structure and the pendant groups, also affect their degradation behavior [32].

In the case that PW composition is uncertain, an alternative solution to obtain the product distribution of high-temperature gasification of solid waste can be using the thermodynamic modeling of equilibrium state. Under some conditions, such as high temperature and/or high residence time, where reaching the thermodynamic equilibrium is probable, thermodynamic equilibrium models could be of great assistance in reducing the complexity and cost of the simulations. Two main methods are commonly utilized in thermodynamic equilibrium modeling; the principle of both is the same and that is the reduction of Gibbs free energy.

The first method is based on the minimization of the total Gibbs free energy of the system that contains the species that are supposed to be present in the reaction environment [101]. The objective is to minimize the total sum of the species Gibbs free energy and the constraint is that the elemental balance should be met and none of the values should be negative. The advantage of using this method, which is also called the non-stoichiometric [102] or differential approach [101], is its self-governing characteristic, i.e., it is independent of any reaction mechanism. This method is advantageous in complex systems where the main reactions are still unknown.

The second model is developed according to the equilibrium constant principle [103,104,105] in which all (selective) reactions are pushed toward equilibrium. Hence, the Gibbs free energy of the system is minimized with regard to the reaction mechanisms that have been chosen [106]. One of the types of this method is called Series Reactor Method. In this method, it is assumed that each reaction is done separately in individual reactors that reach the equilibrium conditions sequentially. The unknown values of the problem are the concentration of the species, which are calculated by solving a set of equations, consisting of elemental balances, energy balances, and the equilibrium constants of the reactions.

#### 4.1.2. Coupling of Available Kinetic Models

Although to the best of the authors’ knowledge, the numerical studies specifically focusing on the gasification of plastics are very scarce [29,57], the kinetic models on different parts of the process, i.e., pyrolysis, homogeneous reactions, and heterogeneous gasification reactions are widely available in the literature. Hence, it is possible to couple those separate kinetic models to construct the overall reaction network for the gasification process. However, this should be done with precautions since each individual model may not be predictive over a wide range of conditions. This is mainly due to the temperature range and presence of gasification agents, in addition to the difference in micro-scale properties of different polymers.

First, many of the available kinetic models for pyrolysis are validated against the experimental data, such as TGA (Thermo Gravimetric Analysis) performed at relatively low temperatures. Usually, the maximum temperature of TGA experiments is 800 °C [29,95,96,107,108,109]) while gasification is usually done in the range of 700–1000 °C [20]. This obviously decreases the reliability of the model, especially because at high temperatures alternative chemistry can become important for both pyrolysis and gasification [110].

Secondly, the presence of the gasification agent can also affect the kinetics of the pyrolysis process and, as is demonstrated here, this effect cannot be captured by simply coupling the pyrolysis and gasification reactions. Pyrolysis of the polymers consists of a series of radical chain reactions [95] of primarily heavy hydrocarbons in the liquid phase with, among others, diffusion-controlled recombination steps [111]. Isolating the intrinsic chemical reaction completely from these physical phenomena is difficult, especially because they are validated against TGA data [112], which may include transport limitations. Hence, if transport limitations cannot be isolated, they need to be considered. This is while the presence of a gasification agent can affect the transport limitations in the liquid phase. As was illustrated by Kashiwagi et al. [113], the concentration of the oxygen in the gasification agent affects the gasification kinetics, as well as the size and frequency of the bubble formation in the melt. Consequently, it is expected that to extend the applicability of a kinetic model for the gasification process, they are validated in a higher temperature range and the presence of the gasification agent [114] and looking at effects such as bubble formation and bubble transport.

#### 4.1.3. Presence of Char

Modeling the char gasification reactions is the other main challenge of gasification compared to pyrolysis. In principle, the gasification of the most common plastics is not associated with a large amount of char that affects the process substantially [20]. However, in the design and optimization of large-scale PWG plants, to increase the syngas quality and optimize the gasification reactions, char gasification can become an important part. This is justified by two main reasons: first, not all the PWG produces a negligible amount of char. PVC can yield up to 4.6% of char after being devolatilized for 15 s at 850 °C [115], FB air gasification of polypropylene can lead to the production of 15% (wt/wt Polypropylene (PP)) of char at an equivalence ratio of 0.2 [116], or 14 wt% of char can be produced in gasification of Polyethylene (PE) packaging boxes in a semi-batch pilot plant [117]. Second, as PWG will start from mixed plastic waste (MPW), chars are inevitably produced in a non-negligible amount because of the presence of other components in MPW, such as fibers, papers [20], dirt, or food residue, which promotes the char forming reactions [97].

### 4.2. Global vs. Detailed Kinetic Models

In general, two different methods are applied: global and mechanistic methods, which are explained in more detail in the following paragraphs. 

#### 4.2.1. Global Kinetic Models

The first and simplest method for kinetic modeling of gasification reactions is the global approach. This method is relatively straightforward and computationally less expensive. The starting point is that a limited number of simplified reactions are considered using pre-defined lumped components, such as gas, oil, and char. First, the decomposition is modeled and then a series of gasification reactions are added that convert the pyrolysis products, e.g., hydrogen, carbon monoxide, carbon dioxide, paraffins, olefins, and char, which can be observed in Figure 4. Hence, the gasification reactions, which mainly include steam reforming, dry reforming, water-gas, water-gas shift, partial oxidation, Boudouard, methanation, equimolar, and hydrogasification reactions, promote syngas production. These reactions are well-known reactions and their kinetic data are usually available in the literature. Nevertheless, the degradation step of polymers is complex and has been treated by different methods and perspectives in the literature.

**Figure 8:**
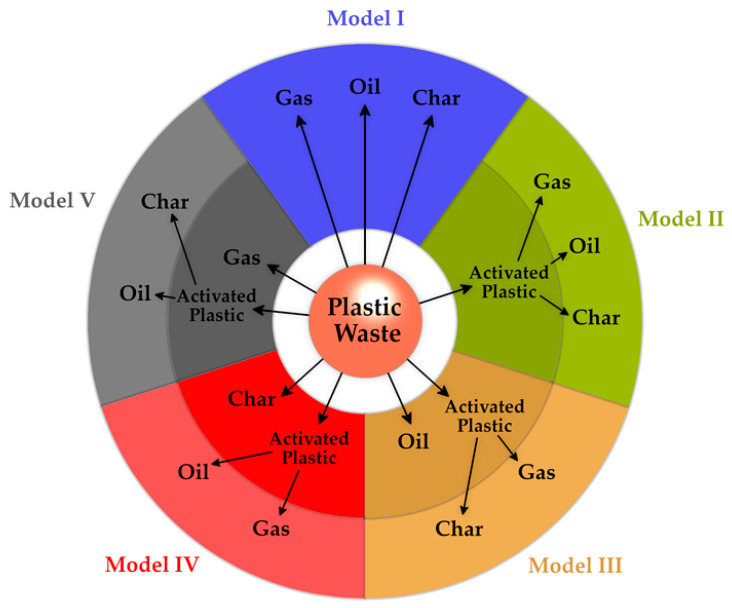
Five different proposed scenarios for the global kinetic model of plastic waste (PE + PS) pyrolysis (adapted from [118]).

In a kinetic modeling study of the thermochemical conversion of PE and Polystyrene (PS), Koo et al. [118] considered five different lumped components—PW, activated plastic, gas, oil, and char—in five different global kinetic model scenarios, including first-order irreversible reactions. The schematic of these different scenarios is illustrated in Figure 8. In four out of five models, the activated plastic is present and converted to different lumped components (gas, oil, char). According to Koo et al. [118], the second model results in the most accurate one for pure PE and PS. Activated plastic in these models is a pseudo-species intermediate product that is ultimately decomposed to the pyrolysis products. This approach—considering intermediate pseudo-species—is a common practice used to reflect the effect of some complex steps—such as “initiation reaction” [119]—which cannot be included in the model due to the computational costs.

In cases where only the degradation rate—and not the product distribution—is of interest and a mixture of different types of feedstock is the target, one opportunity for using the global method is to consider multiple single-step degradation reactions for each component. Then, the kinetic data for each reaction can be obtained by fitting the weighted sum of those reactions against the TGA results [120]. This can get more advanced by assuming multiple reactions for the degradation of each component and considering a distribution of activation energy for those reactions, in a so-called “distributed activation energy model” (DAEM) [121].

In some situations, for the sake of the problem’s economy, it is possible to use some simplifying assumptions. As reported by Martínez-Lera and Pallarés Ranz [122], based on the studies done by Conesa et al. [123] and Hoffmann et al. [124], two orders of magnitude can be the difference between characteristic times of pyrolysis and mixing in the FB gasification of polyolefins at 850 °C. Hence, it is a logical assumption that the devolatilization is an instantaneous step compared to the other ones. Subsequently, for the pyrolysis model, they assumed it as instantaneous. The devolatilization’s product distribution was obtained based on the experimental data as a function of the reactor temperature [122]. Finally, for the homogeneous reactions, they used a global mechanism of 18 reactions, the input of which are the products of instantaneous pyrolysis.

Based on the discussions above, the main advantage of the global reaction kinetic models is their simplicity (maximum few tens of reactions and species—Table 2). This advantage comes with a disadvantage: inaccuracy and limited range of applicability. Determining the lumped components and reactions depends basically on the problem, i.e., the available experimental data [122] and also, the level of details of the feedstock.

Another weakness is the different definitions of feedstock in various models. Hence, it is not possible to reliably generalize a global kinetic model that is obtained for a special type of feedstock with specific reactor and experimental conditions, and use it for another case as a fully predictive model. For this reason, many discrepancies can be observed in the kinetic parameters for the pyrolysis of the same polymer types by different researchers [98]. Consequently, considering the improved computational resources, the models that better describe the molecular interactions of the PWG reactions should be used.

#### 4.2.2. Detailed Kinetic Models

The most comprehensive method of kinetic modeling that can be used for PWG is the mechanistic approach, which provides a detailed description of the radical chain mechanism that is typical for most of the polymers and include initiation, propagation, and termination reaction classes [108]. In this approach, all known initiation mechanisms, the carbon atom number at which this happens, the interaction of intermediate product, and the termination steps, together with the probability of their occurrence, are considered. This way, a network of single-step elementary reactions is created, which describes molecular-level interactions. As a result, the construction of the reaction network is independent of the feedstock. Detailed kinetic modeling of the plastic gasification reactions includes three main steps: description of the feedstock, modeling the pyrolysis/devolatilization of the molten phase, and modeling the gasification reactions. These are explained in detail in the following sub-sections.

##### Feedstock Description

Creating a comprehensive kinetic model without having a correct description of the feedstock is inefficient since it can not predict the product distribution well. Hence, the first step in detailed kinetic modeling of plastic waste is the feed description, which is also demonstrated by Sommariva et al. [125] who studied the modeling of fixed bed gasifiers. Especially for the gasification, in which char gasification reactions play an important role, the feedstock characterization is of paramount importance. As was mentioned before, different polymer types and impurities that are present in the real-world PW can produce a considerable amount of char [20,115,116,117] and this affects the overall kinetic of the PWG process. Moreover, the characteristics of chars from different sources can be different [117] and this can have a remarkable effect on their gasification reactivity [126,127]. 

**Figure 9:**
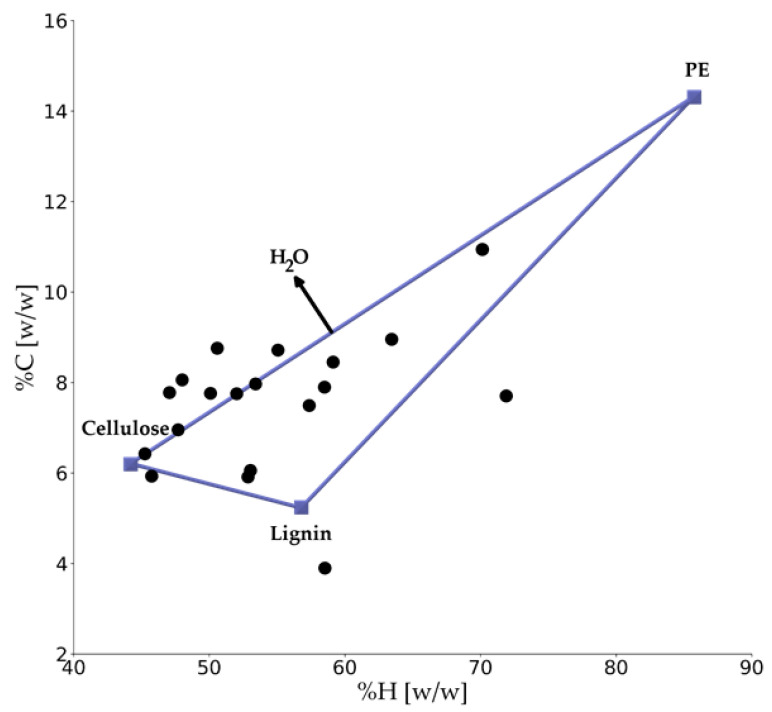
Typical compositions of different RDF in terms of C and H (wt% ash-free) (Redrawn from Ref. [125]). Black circles represent different data points. Reprinted from Computers and Chemical Engineering, Vol. 35, S. Sommariva, R. Grana, T. Maffei, S. Pierucci, E. Ranzi, A kinetic approach to the mathematical model of fixed bed gasifiers, Pages No. 928–935, Copyright (2011), with permission from Elsevier.

If the feedstock of the gasification is an individual polymer type, the starting point can be a well-defined structure and composition [125]. Modeling of the feedstock gets more complicated in the case of mixed plastic waste [128], including different polymer types, additives, and impurities. This is usually done by defining a few reference compounds [125]. As an example, Figure 9 illustrates a Refuse Derived Fuel (RDF) composition by considering PE, lignin, and cellulosic materials, in a framework of carbon and hydrogen weight percent. A similar approach can be also used to describe plastic waste streams. The available databases, such as Phyllis [128] can be used as a reference for determining the initial constituents of the waste stream (as is also illustrated in Figure 9). Besides the databases, the experimental techniques can be used to define the composition of the feedstock, such as, among others, sink–float processes or hyperspectral imaging [129]. To avoid the complexities associated with the abovementioned methods, simplified models for the feedstock can be defined. As an example, McGhee et al. have considered a combination of PVC and biomass as a model for MSW [130]. 

After determining the initial composition of the waste stream, the detailed description of each compound, for which the multi-step devolatilization kinetic is going to be applied [125], should be determined [131]. If a virgin neat polymer is considered, the most important parameter in this regard is the chain length distribution. Also, a simplification can be to assume an idealized monodispersed polymer [132].

##### Devolatilization

Upon having a clear picture of the feedstock, the devolatilization modeling can be applied. In the devolatilization step, pyrolysis in the molten phase and the subsequent evaporation are present. The evaporation is discussed in Section 6.2 and the focus of this subsection is on the pyrolysis reactions. Two of the common approaches that are implemented in the mechanistic modeling of polymer degradation are Method of Moments (MOM) and Kinetic Monte Carlo (kMC). The former, which is a continuous kinetic modeling technique [17], has been utilized more frequently for mechanistic modeling [99]. A detailed description of each method is provided by Dogu et al. [98].

The challenge of using these methods is their high computational costs, which makes it difficult to use them in a multi-scale framework such as reactive CFD simulations. However, from the computational cost point of view, an advantage is found in the MOM approach compared to the kMC, because it reduces the number of mass balance equations of the chain species and provides average-based data in each time step, such as averages of molecular weight and branching density [133]. Nevertheless, this advantage is brought along a disadvantage of providing imprecise information on the monomer sequences and structural defects [98]. Moreover, it doesn’t give a detailed distribution of the molar mass and branching density [133]. On the other hand, kMC method is a stochastic approach that can track each molecule [134] and provide more accurate and robust results. However, this is done at the expense of computational and time resources. Once the reaction families, mechanisms, and the initial species to be present in the network are known, computer software packages [95,135] should be used to create a huge network of individual reactions [98] out of the pre-defined families of compounds and reactions. Hence, the crucial part is to understand all the possibilities and reactions that could happen and include all of those in the kinetic model.

**Figure 10:**
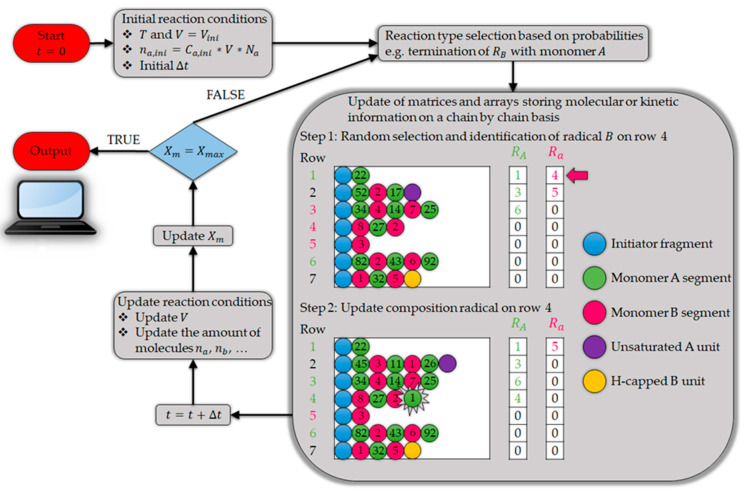
The flowchart of the mechanistic modeling approach (kMC) for the polymerization process developed by De Smit et al. [136], which tracks individual species and provides detailed information about the molecular structures within a chain. Reproduced from [136] with permission from the Royal Society of Chemistry.

A newly developed kMC-based method by De Smit et al. [136] can be used to predict more accurately the kinetics of polymerization as well as the thermo-chemical recycling of the polymers. This can be done by tracking each molecular structure within a chain and handling the long polymer chain data [136]. As can be observed in Figure 10, the method starts from the initial reaction conditions, followed by the selection of the reaction type, which is based on their probabilities. The probabilities are a function of the kinetic data, the molecular diffusion parameters, and the reactant concentrations. Afterward, as the result of the reaction, the initial data regarding the molecular structures change and the matrix-based data storage is updated for the sequence and segment length. Subsequently, the reaction conditions and the conversion is updated [136]. This procedure is illustrated for polymerization, but the same logic is applied for the reverse reaction, which is thermochemical conversion. As it can be inferred from this procedure, and as was mentioned before, the computational cost of this method is high. In this regard, hybrid kMC models exist that can be an option to decrease the computational load of the reaction kinetic modeling efforts [98].

##### Gasification

Detailed kinetic modeling has been also developed for the homogeneous and heterogeneous gasification reactions of PWG in addition to the pyrolysis. For the former, an opportunity lies in the numerous kinetic models for different fuels that have been developed and are available in the literature. As an example, the CRECK Modeling Lab (Milan, Italy) [137] developed different mechanisms, starting from a mechanism for syngas with 21 species and 62 reactions up to a mechanism for C1 to C16, at low and high temperatures, including 621 species and 27,369 reactions [137].

For the heterogeneous char gasification reactions, usually, the global single-step reactions are considered, which are illustrated in Figure 4. Char from the waste pyrolysis has a porous structure [138]. Consequently, at high temperatures [139], internal heat and mass transfer models should be coupled to the kinetic models to better predict the apparent rate of the char gasification reactions. However, the common models for char gasification, such as the unreacted shrinking core model [139] and homogeneous model [140] don’t consider the diffusion limits. Only the random pore model [141] accounts for the porous structure of the char, by including the porosity, pore length, and the specific surface area in the reaction kinetic equation [142].

The activity of char can also affect the kinetics of char gasification reactions. The loss of reactivity due to the so-called “thermal annealing” can be captured using (semi-)detailed approaches [143]. Chemisorption of the oxygen-containing gases on the surface to form carbon-oxygen complexes, the transformation of the surface species, and desorption of the oxygen-containing products can be the steps to be considered in the semi-detailed mechanism [143]. Overall, the rate-determining steps in the gasifiers can be the char gasification [125], and hence, it should be modeled precisely to reflect the correct performance of the whole reaction kinetic network.

##### Challenges and Opportunities

As an example of the kinetic models that are specifically developed for the gasification of PW, Horton et al. [57] developed a molecular-level kinetic model for the gasification of common plastic types. This model includes 283 reactions and 85 species accounting for pyrolysis, char formation, and gasification reactions. To the best of the authors’ knowledge, other PWG studies use global kinetic models.

*Coupling*

Despite the related challenges (Section 4.1.2), coupling different detailed kinetic mechanisms can end up in a united PWG framework, with some advantages:

1. The mechanistic models are feedstock independent. Hence, they are supposed to perform properly for different compositions and feedstock characteristics. Moreover, the similarities in the polymer segments and reaction families make it less burdensome to introduce new polymer types.
2. The presence of different gasification agents with different concentrations can be taken into account in a single model. This may also reflect the synergistic effects as a result of gasification with multiple gasification agents. As it can be seen in the developed detailed kinetic models [137], all the gasification agents are present and based on their concentration, their contribution to the overall gasification process is accounted for.
3. To introduce new species, only the initial propagation and decomposition steps should be defined [107]. Hence, reliable modification of the model can be done easily in this approach.

*Size of the Reaction Network*

The detailed kinetic models that can be used in the PWG are supposed to include a large reaction network: The detailed kinetic mechanism for a simple oxidation case of C_1_-C_16_, can include 621 species and 27,369 reactions [137], or the automated reaction kinetic network of naphtha steam cracking (which can be considered as a similar process to gasification), can encompass 1947 species and 82,130 reactions [144]. For polymers, the network size can grow exponentially. This can pose two important challenges: on the one hand, generating such a huge network is a cumbersome task; On the other hand, implementing the produced reaction network in higher scale frameworks, e.g., CFD, is unfeasible because of their high computational costs. To overcome these two challenges, some possible solutions are introduced in the next paragraphs.

For the first challenge, i.e., creating the reaction network, the existence of many species and reactions of similar types may allow applying the single event approach and Evans-Polanyi relation [145]. This imposes a dependency of the kinetic parameters for similar reactions and hence, decreases the design and computational efforts in creating the network of a large number of reactions. Besides that, automatic network generation tools have been developed over the past decades, which are discussed more in the next paragraph.

**Figure 11:**
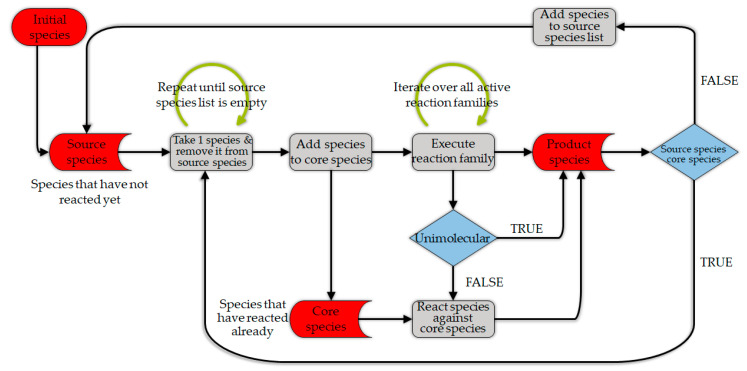
The algorithm of Genesys to create a reaction network by applying the pre-defined reaction families on the unreacted molecules (Redrawn from Ref. [135]) Reprinted from Chemical Engineering Journal, Vol. 207–208, Nick M. Vandewiele, Kevin M. Van Geem, Marie-Françoise Reyniers, Guy B. Marin, Genesys: Kinetic model construction using chemo-informatics, Pages No. 526–538, Copyright (2012), with permission from Elsevier.

State-of-the-art network generators, for example, the Reaction Mechanism Generator (RMG [146]) or Genesys [135], use a series of user-defined reaction templates to iteratively expand a network of so-called elementary reactions. Figure 11 illustrates the flow diagram that is used in an automatic reaction network generation (Genesys), proposed by Vandewiele et al. [135]. Many challenges in automated network generation have been successfully tackled over the years—including symmetry detection of molecules [147], taking stereochemistry into account [148,149], and predicting molecular properties [150,151]. However, one challenge still remains: kinetic model generators all rely on pre-defined reaction templates, which limit the reactions in the model to those anticipated by the user or developer. One method that has shown potential for explorative reaction network generation is potential energy surface scans, the details of which can be found elsewhere [152,153].

For the second challenge related to the large size of the reaction network, it is possible to reduce the number of reactions and species in a controlled (rule-based) manner. This can be done via the so-called reduction methods, before or after the generation of the detailed kinetic models [131]. The former is done by applying some reaction network parameters limits, e.g., the molecule size, type, number, and reaction families, among others. If this method is not done with enough care, it can cause an unconscious loss of information. This is because there could be some important molecules and/or reactions, the presence of which has not been even taken into account from the beginning because of limiting the types of molecules, reactions, etc. Hence, to cautiously perform the mechanism reduction, the best approach for the PWG process is the second option, which is to create the full reaction network first and then, intelligently reduce its size. Different reduction techniques in this option are global reduction, response modeling, chemical lumping, statistical lumping, and detailed reduction [131]. The most common ones are lumping and detailed reduction, which are described in the next paragraphs.

In the lumping method, the chemical species are lumped together based on their chemical structure or reactivity [107,154]. The species that are in equilibrium with each other can be also lumped together [131]. For this method to be applied, a fundamental understanding of the reaction chemistry is required to reliably lump the species and reduce the mechanism [154], without losing too much information. Lumping can also be done according to some predefined mathematical rules [154,155].

In the other common reduction technique, which is the detailed reduction, e.g., the one proposed by Wang and Frenklach [156], the reactions are removed based on the redundancy principle [157]. This means that the less important reactions—compared to predefined limiting reactions—are removed [131,154]. In contrast to the other reduction methods, which are usually case dependent, this method is more general [131]. Besides the detailed reduction method that tries to remove the reactions, similar methods can also be applied to remove species. For example, in the directed relation graph (DRG) method [158], the species that have a less important role in the production of crucial species (which have been selected before), are removed from the network [154,157].

It is also possible to couple different techniques to efficiently and reliably reduce the available mechanism. Hence, an initial reduction can be performed by lumping the species and reactions and then reducing them based on the detailed reduction or similar methods. Stagni et al. performed a similar reduction procedure for the detailed kinetic mechanism of n-heptane and n-dodecane oxidation and implemented the final results in CFD simulation of the laminar flames [154]. 

Based on what was discussed on different kinetic modeling approaches, including the global and detailed ones, as well as the reduction methods, Table 2 summarizes the main features of each approach, with some perspective on the gasification of plastic wastes.

**Table 2:** Overal comparison of different kinetic modeling approaches with a focus on multi-scale modeling of PWG.

	Global Modeling	Mechanistic (Detailed) Modeling	
		MOM	kMC
Requires detailed feedstock description	No (pre-defined lumps)	Yes	
Degree of complexity	Low	Medium	High
Degree of details on the product description	Low	Medium (average properties) [98]	High (full molecular detail) [98]
Computational cost	Low	Medium	High
OM of number of species	50	100–1000 [154] (Reduced: 10–100)	1000–10,000 [154] (Reduced: 10–100)
OM of number of reactions	50	1000–50,000 [154] (Reduced: 100–1000)	1000-50,000 [154] (Reduced: 100–1000)
Common application	CFD/1D Models	1D Models (Reduced: CFD)	
Feedstock independent	No	Yes	
Reliable coupling to other kinetic models	No	Yes	
Adaptability to new species (and gasification agents)	No	Yes	
Reliable temperature extrapolation	No	Yes	
Needs reaction network generator (extra complexity)	No	Yes	
Ability to consider dynamic char activity	No	Yes	

#### 4.2.3. Validation Challenges

Validation of kinetic models is challenging because of the complex behavior of feedstock, limits in the experimental techniques, and the dependency on the flow regime. Regarding the first, the synergistic effect of the plastic mixture is a fact that has been proved previously [29] and is difficult to be addressed in a kinetic model. According to Briceno et al. [159], the apparent activation energy of the degradation for a mixture of PP and HDPE is lower than the pure HDPE, and for the mixture with high content of PP and HDPE, is lower than the pure PP. Predicting the interaction between different polymers is not an easy task, since it is a function of different parameters, such as the reactivity of their intermediate products, the similarity of the polymer types to each other, the concentration of each component, and their mixing degree [160].

The second problem is the validity of the experimental data. Many of the kinetic models are validated against the TGA data, while there are different problems associated with these data:

1. TGA data include the evaporation rates, which are not equal to the degradation rates. So, if a kinetic model is validated against it, in FB regimes with higher evaporation rates, it is supposed to underestimate the devolatilization rate (if the evaporation and degradation models are not decoupled).
2. The reactive environment affects the degradation and the evaporation rate of polymers, as was discussed in Section 4.1.2.
3. It can include the internal heat and mass transfer limitations, which are not considered in the kinetic models. For large sample sizes [161], providing the isothermal conditions is not possible, and for samples with weak mass transfer properties, concentration gradients are observed within them [162]. Even if in a kinetic model, the effect of diffusion limits on the kinetic parameters is considered [111], two other problems can be raised: First, this shows the incapability in deriving the pure intrinsic kinetic data; and second, the mixing degree and mass transfer limitations can be different from the conditions in which this kinetic model is derived. Hence, this increases the uncertainty in using this kinetic model in different conditions.
4. It is not possible to measure the concentration of reacting species in the liquid phase, or the products right after being produced in the gas phase. Hence, secondary reactions can and will happen.
5. The uncertainty related to enough sensitivity of the balance used in the TGA instrument is another challenge [162].
6. The effect of radiation on the sample in high temperatures is different for the samples with different absorption properties [162].

The value of TGA-based data for measuring degradation rates has been heavily debated for decades [17]. To overcome some of the problems, TGA experiments should be modified so that heat and mass transfer play a less dominant role [161]. Moreover, new experimental methods have been developed in recent years, which can help improve obtaining the intrinsic kinetic data, such as fluidized bed TGA (FB-TGA) [163], which is also called micro fluidized beds (MFB) [164], and microwave thermogravimetric analyzers (MWTGA) [165] (the details of which are not in the scope of this review).

The last important point is that the kinetics can also rely on the flow regime and upstream of the process. If a high mixing level in the molecular scale is achieved beforehand, the produced radicals from different polymer types can interact and promote or inhibit the volatilization phenomena [96]. This may affect the product distribution, though small.

## 5. Internal Transport Limitations

The observed devolatilization rate is largely affected by internal transport phenomena, besides the intrinsic kinetics. Indeed, due to the fast reaction kinetics of pyrolysis and gasification [122,166], the internal heat and mass transfer may become the rate-limiting steps [161,167] (especially because plastics have shown poor heat and mass transfer characteristics [52,168], even in the molten phase [169]).

### 5.1. Internal Mass Transfer

#### 5.1.1. Solid Phase

Considering the solid phases present in the system (not-melted plastic and char), the internal mass transfer limitations are only important for the char gasification reactions, because in the solid plastic particles, until they are melted, no significant gas-solid interactions have been reported in the literature. Determining the role of mass diffusion limits for the char gasification reactions in the PWG process is a challenge due to the often-unknown properties of char. Chars from different feedstock, or produced under different conditions, have a variety of characteristics and morphological properties. First, the pore distribution function should be defined, and then, depending on the temperature and pore sizes, the importance of internal mass transfer can be assessed [170]. At the common temperature range of gasification (700–1000 °C) [20], the role of molecular or Knudsen diffusion is more important than the gasification reaction [170]. This has been confirmed also in the simulation framework recently developed by Schulze et al. [171] who assessed the CO_2_ gasification of char in a TGA instrument. This trend can be changed due to the decrease in char reactivity caused by thermal annealing, which should be assessed via the coupling of a semi-detailed model of char gasification and diffusion models [143].

#### 5.1.2. Liquid Phase

The mass transfer limitations inside the molten plastic phase are important phenomena to be taken into account [172]. As was discussed in the previous section, the diffusion of radical species and their mixing has an important effect on the observed recombination and termination reaction rates [96] during plastic pyrolysis. This becomes even more pronounced when different types of polymers are mixed and a wide range of intermediate products makes multi-component diffusion problems very complicated. This is further discussed in Section 6.2.2: Multiple Components.

Another important limit can be the resistance against the transfer of bubbles formed inside the liquid layer [173]. This may be more clear by referring to Figure 5. In this figure, it is assumed that pyrolysis and evaporation happen in the liquid layer. Hence, it is possible that some cracked species are evaporated, which should be transferred to the surface to participate in further pyrolysis and gasification reactions. In cases of the very small length scale of the liquid layer (e.g., when a very thin layer of molten plastic covers the solid fluidization agent [45]), it is logical to neglect this resistance. Otherwise, where the length scale of the liquid layer is large, this limitation should be considered because if the bubble transfer time is not short enough, the mass transfer between the vapor and the liquid phase can result in a composition change of the bubble. To simulate this phenomenon, the modeling and simulation work on the microbubble rising in a liquid phase can be inspiring [174]. 

##### Simplifying Assumptions

The discussions above concern the PWG, which is a reactive system. Nonetheless, even for simpler, non-reactive cases of multi-component evaporation, a normal practice in modeling such cases is to neglect the internal mass transfer limitations [175,176,177,178,179]. This can be a correct assumption if other phenomena in series with the diffusion are rate-limiting, or if the size of the liquid droplet or the thickness of the liquid layer is so small that the rapid mixing (or infinite diffusivity [180]) assumption can be applied [176]. However, in the case of a large droplet/thick layer of molten plastic, rapid mixing is unlikely. It has been shown that even at the very high circulations inside the liquid phase (which can result in rapid mixing), the length scale of the diffusion can be reduced only to a maximum of three times [180]. Hence, especially in the high viscosity liquids, such as molten plastics, in which the internal circulations (and hence mixing) are weaker [51], infinite diffusivity can be a very strong assumption. 

In modeling the mass transfer limitations in multi-component evaporation cases (which happens during PWG) other simplifying assumptions also exist that should be treated carefully for the PWG process. First, which is more important for the droplet phase, is assuming symmetry in internal transfer phenomena in the liquid phase. This has been used in many references in the related fields, such as evaporation [172,173,175,177,181,182]. This assumption requires that internal motions and non-radial gradients inside the liquid droplet are neglected [172], which is not always the case for the PWG processes. The internal motions can be due to the thermocapillary effects, which are called the thermocapillary Marangoni convection, and is the result of surface tension gradients [91,172]. Another possibility is that the internal recirculation motions in the systems are due to the high convective fluxes and particle movements, which affect the internal heat and mass transfer [173]. These effects can be taken into account by either applying a correction factor and deriving an effective parameter or modeling the internal motions [90]. The former is a more computationally efficient method.

### 5.2. Internal Heat Transfer

Modeling the heat transfer may be more crucial than the mass transfer, due to the effect of temperature. With typical PWG activation energies and at typical temperatures for PWG, a deviation in temperature of merely 10 °C can change the reaction rates by over 20% [166]. This demonstrates the importance of correctly predicting the temperature on all the scales.

#### 5.2.1. Solid Phase

For the solid phase, the important point to take into account is using the effective thermal conductivity (Section 3.2) due to the porosity of the plastics [87] and char [138]. However, due to the high thermal conductivity of the char core [183], the Biot number of this phase in the PW gasifiers becomes low and consequently, it is logical to neglect the heat transfer resistance and assume a uniform temperature inside it.

#### 5.2.2. Liquid Phase

Regarding the liquid phase, the temperature difference throughout the droplet can become large (up to 80 °C [51]). This temperature difference can cause a change in the rate of pyrolysis reactions by an order of magnitude or even more. The temperature distribution inside the molten plastic can be modeled via the simple conductive heat transfer term that appears in the energy equation. However, the challenge is to simulate—or include the effect of—thermocapillary Marangoni convection and internal circulations, which was also discussed in Section 5.1.2: Simplifying Assumptions. This type of heat transfer can be also called circulative heat transfer [51]. In high-temperature convective regimes, the internal circulation has a great impact on the temperature distribution in a liquid droplet [51]. To assess the effectiveness of the thermocapillary convection on the temperature distribution inside a liquid droplet, the dimensionless Marangoni number can be used [91,184]:

(1) $$Ma=-\frac{d\sigma }{dT}\times \frac{\Delta T\;c_{p}L\;\rho }{\lambda \;\mu },$$

where Ma is the dimensionless Marangoni number, the term $\frac{d\sigma }{dT}$ indicates the variation of surface tension with temperature, $c_{p}$ is the specific heat capacity, L is the characteristic length, ρ is the fluid density, λ is the thermal conductivity of the fluid, and μ is its dynamic viscosity. This number shows the ratio between the effect of Marangoni transport to diffusive transport. For each case, based on different liquid properties and particle size, a critical Marangoni number can be defined [91,184] above which the Marangoni effects should be taken into account in the internal heat transfer modeling.

The effects of the circulative heat transfer can be to an extent that, the minimum temperature occurs at a location close to the center of the Hill vortex, and not the droplet center (Figure 5). Wong and Lin [51] concluded that although the vortex model can qualitatively predict the temperature trends, they can not precisely reproduce the experimental temperature distribution through the liquid droplet. Hence, they applied an effective conductivity term in their simulations to account for both the conductive and circulative heat transfer. 

The study done by Shinjo et al. [185] is a great hint for detailed modeling of temperature distribution in molten plastic under pyrolysis reactions in a convective regime. The goal of their study is to model the heat transfer in a water-oil emulsion and can be ultimately used in the assessment of the micro-explosion and puffing of the water in the emulsion. This phenomenon can be analogous to the formation of bubbles due to the cracking of the species in the molten liquid. They developed a model (including the Marangoni effects) for the heat transfer inside the liquid particles in a convective regime, which incorporates the angular dependency of the effective thermal conductivity and takes into account the eccentricity of the temperature distribution inside the liquid droplet.

As can be inferred from this part, an important challenge in modeling the heat transfer in the particle scale of the PWG is the internal motions. Hence, the effect of viscosity becomes very crucial because of its effect on the internal motions and temperature distribution inside the liquid droplet. From Equation (1) and also as was demonstrated by Wong and Lin [51], higher viscosity of the liquid results in

1. A weaker effect of Marangoni convection (and hence weaker internal motions or circulative heat transfer); and
2. Monotonically decreasing temperature profile toward the center of the droplet.

Hence, due to the high viscosity of molten plastic liquids (η>10 Pa·s [186]), to decrease the computational costs, it is logical to neglect the internal circulative heat transfer. Nevertheless, this should be further studied because, first of all, the molten plastics have shown non-Newtonian behavior [187,188]; and second, the temperature affects their rheological behavior [189]. Hence, it can be concluded that in high temperature and strong convective fluxes of the PWG, the viscosity of molten plastic can become low and result in a high Marangoni number.

Considering all of these complexities and uncertainties regarding the important (or on the other side, negligible) influence of thermocapillary effects on the internal heat transfer, one of the best approaches to assess this problem can be using high-resolution numerical simulation, such as particle resolved direct numerical simulation (PR-DNS). 

It can be concluded that considering the complexities of simulating internal heat and mass transfer besides the improved simulation frameworks and computational resources, the best approach is to first assess in detail the degree of impact of ignoring those complexities. The work of Haim and Kalman [190] is an example of such an assessment; they coupled an internal heat conduction model to an Eulerian–Lagrangian framework to determine the conditions in which it is possible to neglect the internal heat transfer resistances.

## 6. Phase Transformations and Interfacial Transport Phenomena

In this section, first, the available approaches for the melting process are reviewed together with their applicability and challenges for the PW. Afterward, different approaches that are available for modeling the evaporation of a multi-component hydrocarbon droplet are discussed. Finally, the interfacial heat, mass, and momentum transfer are assessed, which can affect both melting and evaporation as well. 

### 6.1. Melting

The melting process is an important step and can be performed in-situ, meaning solid feeding, or ex-situ, meaning feeding with an extruder or so. There are some advantages in ex-situ liquefaction of PW in the PWG [30] including mild cracking of plastics, dehalogenation, and decreasing the thermal load of the reactor, among others. However, in some other cases, the plastics are fed into the reactor as solid particles. 

At the particle scale, melting is a non-linear displacement of the interface and its rate is a function of the amount of absorbed or desorbed latent heat at the boundaries [191], described by the following equation, called the classical Stephan condition:

(2) $$\rho L\left(\frac{ds}{dt}\right)=\lambda_{s}\left(\frac{\partial T_{s}}{\partial x}\right)-\lambda_{l}\left(\frac{\partial T_{l}}{\partial x}\right).$$

where ρ is the density, L is the latent heat of fusion, s is the solid-liquid interface position, $\lambda_{s}$ and $\lambda_{l}$ are the thermal conductivity of the solid and liquid phases, respectively, $T_{s}$ and $T_{l}$ are the temperature of the solid and liquid phases, respectively, and x is the spatial coordinate.

Melting of polymers and its modeling is associated with some extra complexities, which are explained in the following subsections.

#### 6.1.1. Melting Phenomenon

Melting of polymers is more complicated than lower molecular weight molecules for three reasons:

1. The presence of long-chain molecules; in the melting process, these long-chain molecules should get aligned [192].
2. The associated melting kinetics of polymers are more complicated compared to, e.g., metals [193], amongst others, due to their molecular chain folding [192]; and
3. It has been recently demonstrated that polymer melting can be a continuous phenomenon at the molecular scale [194].

In addition to the points above, high melt viscosity, low melting temperature, and partial crystallization characteristics—which becomes even more complicated in the case of PW that contains impurities [193]—increase the complexity of modeling melting in the PWG process. Finally, the cooling effect, which is usually neglected [195], can be another complexity. 

Considering these complexities, it is necessary to assess the melting process fundamentally at the molecular level to improve capturing the behavior of different types of plastics in all phases under a heating environment. Similar to the thermophysical properties, the fundamental understanding of the melting process can be done via the molecular simulation approaches [194,196,197], the details of which are not explained in this review. In the following sub-sections, the focus is on the models implemented for melting in the particle scale.

#### 6.1.2. Melting Models

In the following sub-sections, different general approaches that are available to model the melting process of polymers are explained, being extrusion, enthalpy, phase field, and reaction models.

##### Extrusion Models

The main modeling and simulation studies of plastics and polymer melting date back to the 1960s [71,198] when the main focus was on screw extruders [199]. In most of these simulations, the model is constructed based on:

1. The conventional conductive heat transfer
2. The pressure and shear forces [200]
3. The characteristics of the extruder, e.g., the rotational velocity [71]
4. Simplifying assumptions such as sharp melting of the plastics.

Hence, this phenomenon is modeled in an extrusion process, without any transition in which plastics are not typically reacting or forming a gaseous state. These types of models are in fact multi-scale frameworks for the melting process in an extruder, which include scale-bridging techniques the goal of which is to reduce the complexities associated with the smaller scale phenomena. However, more recent research on extrusion modeling includes also the particle-resolved simulations in which progressive melting of particles is considered. As an example, Celik et al. [201] considered the pellets as discretized particles in simulating single-screw extrusion. In this approach, each particle is considered as a sphere with a desired number of shells. In each time step, the temperature of each shell is calculated, and based on the melting rate, the melting of the outermost layer is simulated.

In this approach, which is called “front tracking” [202], a sharp interface between the molten phase and the solid phase is considered (Figure 5b). However, in reality, polymer melting is characterized by a continuous transition between the solid and liquid phases, in which both of them are present in the melting zone [202] (Figure 5a). Hence, the front tracking models are in fact a specific form of continuous models, but with an infinitesimally small thickness of solid-liquid interface and the changes between the two zones are step changes. Consequently, they are not able to reconstruct the transition zone between solid and liquid. To get a better insight into the melting process at particle scale with higher resolutions, two other approaches can be helpful, which are enthalpy-based and phase-field models. The first one is considered to be an easier approach [203] compared to the second one and has been used more frequently in the literature.

##### Enthalpy-Based Models

In the enthalpy-based models, the melting region is considered as a mixture of solid and liquid phases. Then, the temperature or enthalpy distribution throughout this region is obtained via solving a continuous heat transfer model, without specifying the solid or liquid phase. However, the phase properties, such as heat capacity, are different for the solid and liquid phases. This is considered in the heat transfer model by assuming a continuous function for that property from the solid phase to the liquid phase. This way, it is possible to take into account the latent heat evolution [202] in the melting zone (mushy zone). The share of phases in the melting zone is determined based on the volume fraction [70,72,204]. In fact, the interface is reconstructed as a region with a distribution of the liquid volume fraction [203]. This volume fraction can be expressed based on the enthalpy or temperature distribution. Different functions can be used in this regard. As an example, Wang et al. [204] have considered Equation (3) to define the volume fraction as a linear function of enthalpy of solid and liquid phases:

(3) $$\alpha_{l}=\{\begin{array}{cc} 0 & if\;H\leq H_{s}\;\\ \frac{H-H_{s}}{H_{l}-H_{s}} & if\;H_{s}<H\leq H_{l}\\ 1 & if\;H>H_{l} \end{array}.$$

where α is the volume fraction, s and l subscripts define the solid and liquid phases, and H indicates the enthalpy. Hence, in this method, since one equation is considered for the whole region, the discretized space to solve the governing equation doesn’t need to be changed according to the interface displacement (the Stephan problem [191]). This approach is called the fixed-grid approach [202].

The application of the enthalpy-based model is straightforward because it is sufficient to only add a transition function for the enthalpy or specific heat capacity [70,72]. 

##### Phase-Field Models

In the phase-field modeling approach, the transition between the solid and liquid phases is reflected as the free energy density at the interface, instead of the latent heat and enthalpy. Except for this difference, the logic of implementing this modeling approach is similar to the enthalpy-based approach. The free energy density at the interface is defined as a function of a crystal order parameter [193]. This parameter determines the free energy density and changes in the boundaries while melting [205]. It is a continuous function ranging from zero at the melt state to one at the solidified state [193].

The advantage of the phase-field model over the enthalpy-based model is that it is based on the crystal structure of the polymer and this provides the possibility of developing the model based on the plastic type more fundamentally, rather than using the enthalpy of solid and liquid phase, which are usually derived experimentally [201]. However, the challenge associated with the phase-field model is obtaining the free energy density function of the feedstock [205]. Moreover, the phase-field model is designed only for the transformation process, in which the melting has already started and the interface is known. In other words, it cannot be used to simulate a process, during which the melting starts [205]. Consequently, to model starting the melting process during PWG, the enthalpy-based one is likely to be the better approach.

##### Reaction-Type Models

To decrease the computational costs while reflecting the melting process in the simulation framework, another modeling approach is to assume melting as a reaction that converts the solid phase to the liquid phase [38]. This has been done by introducing the kinetic parameters [38] of the “melting reaction”. It is possible to obtain these parameters via differential scanning calorimetry (DSC) techniques [38,206]. This approach incorporates the effect of the melting process in the global time scale of the PWG while avoiding the detailed complexities of modeling this process. 

#### 6.1.3. Application in the Multi-Scale Framework

Different modeling approaches of melting can be incorporated into various multi-scale modeling frameworks of the multi-phase flows. As was mentioned in Section 6.1.2: Extrusion Models, the extrusion model can be considered a multi-scale framework. Besides, the enthalpy-based and phase-field models can be implemented in other multi-scale modeling frameworks, such as the Lattice-Boltzmann Method (LBM) [203,207] or Volume of Fluid (VOF) [208]. It is worth mentioning that implementing this process in the multi-scale simulation framework is challenging. This can be inferred also from the limitations of modeling melting even in the commercial simulation packages [208]. These limitations demonstrate that multi-scale modeling of the PWG in one software package is challenging and hence, further studies need to be done to implement modeling this phenomenon in a multi-scale modeling framework.

### 6.2. Evaporation

Evaporation is a crucial step that transfers the pyrolysis products to the gas phase where the main oxygen-containing gasification reactions take place. In this section, the focus is on the general modeling approaches of evaporation, together with the complexities and simplifying assumptions that are usually implemented in this regard.

#### 6.2.1. From 0D to 1D Models

The simplest approach to modeling evaporation is the zero-dimensional framework, which is usually used in CFD to save computational costs [209]. In this approach, the temperature and concentration distribution throughout the liquid droplet or the interface is not of interest, and heat and mass transfer inside the droplet is considered infinite. Overall, the evaporation mass rate and the change of the liquid droplet size are the most important results that are obtained in this approach. Using the 0D model may result in a deviation from the experimental data, e.g., predicting only slow evaporation rates, while not performing well in the high evaporation rates [209].

To increase the simulation results precision, a one-dimensional framework with finite heat and mass transfer is considered. This is done by assuming the presence of symmetry in the particle shape, as well as the internal transport phenomena. Implementing these models in higher-scale simulation frameworks significantly increases the computational time. In this approach, the classical heat and mass balance equations inside the droplet can be coupled to the film theory [210,211] that is usually considered in modeling the evaporation process. If the evaporation rate is not large, or convective fluxes are not important, the problem can be considered as so-called diffusion-controlled [208] and it is modeled by having the mass transfer coefficient and the concentration gradient between the interface and bulk gas phase. On the other hand, in cases of high evaporation rates in which the Stefan flow becomes important, or in cases of high flow of gas, e.g., in fluidized cases in which the convection becomes important, the Sherwood and Nusselt numbers should be incorporated into the equations [212]. This situation can be called the convection-diffusion controlled model [208]. This is an important feature that can be used for the FB gasification systems in which high relative velocity (gas to solid/liquid slip velocity) is desired to intensify the heat and mass transfer.

**Figure 12:**
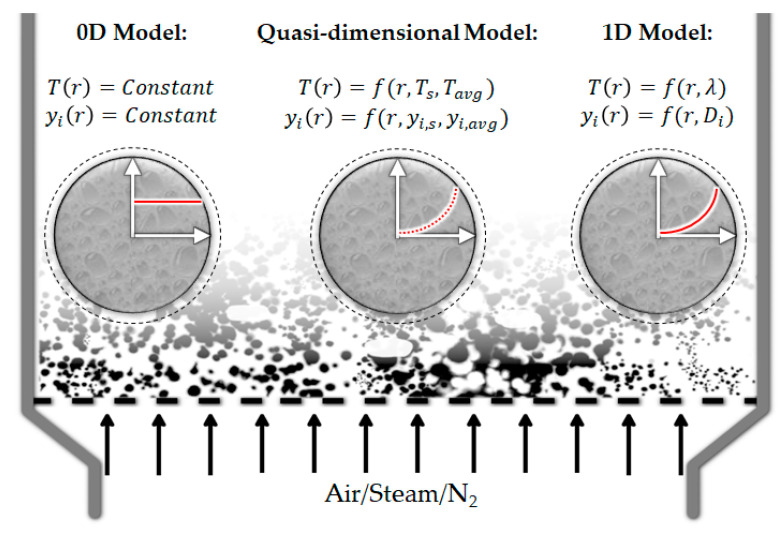
Schematic illustration of different approaches in modeling vaporization (adapted from [209]). The red line and curves are related to the temperature and concentration profiles within the liquid region in a multi-component fuel under vaporization. The dashed circles around the droplets represent the gas-liquid interface.

A transient modeling approach between 0D and 1D is called a quasi-dimensional model [209,213]. In this approach, which has been used for non-reactive systems, instead of solving the transport equation for the internal heat and mass transfer, a presumed quadratic polynomial is considered for the temperature and concentration gradients within the multi-component liquid. The constants of these polynomials for the temperature profile are expressed as a function of the surface and average temperature. In a similar manner, the concentration profiles are derived. Consequently, the concentration and temperature gradients are implicitly taken into account in the evaporation model, while avoiding adding heat and mass transfer equations into the system. Hence, the imposed gradients within the droplet account for the internal transport limitations. Figure 12 illustrates three different modeling approaches that were explained above.

#### 6.2.2. Modeling Complexities for PWG

##### Multiple Components

The most crucial aspect in modeling evaporation in the PWG process is the multi-component nature of the molten phase. The molten phase in PWG is supposed to include a wide range of molecules from small to heavy hydrocarbons, together with other impurities. Each of these components with different thermophysical properties makes modeling this phenomenon complicated. Even in the case of a few components, precisely calculating the evaporation rates is considered a difficult task [214]. 

Different approaches have been proposed to include different components in an evaporation model. These approaches are continuous multi-component (CMC), discrete multi-component (DMC), and hybrid multi-component (HMC) models [179].

In the CMC model, the multi-component liquid is considered as a distribution of molecules based on, e.g., molecular weight, and methods of continuous thermodynamics are used in this regard [215]. This approach is well suited for PWG, in which the liquid droplets may encompass a wide range of products. However, although it saves the computational costs, this cannot be coupled to the detailed kinetic models, since it cannot track each individual species [179]. DMC is, on the other hand, a simulating approach in which all the species are tracked individually. This method is the normal practice in most of the evaporation studies (Table 3).

In the hybrid model, different classes of species are defined and a distribution of a characteristic, e.g., molecular weight, is assigned to each class [179] to reflect the presence of different species in each class. A class may also include only one species. So, in principle, the problem is reduced to the number of classes instead of the number of individual species, while it is possible to track the changes in the distribution of species in each class. This approach is somehow similar to the lumping procedure or global kinetic modeling approach in kinetic modeling. An important and challenging fact about this approach is how to couple this model to a kinetic model, which should be further investigated. The hybrid models can be computationally efficient and their results are acceptable compared to the DMC approach [179]. Overall, if the goal is to couple the evaporation model with the chemistry, considering improvements in the computational resources, the only available solution seems to be the DMC approach.

##### Mass Fraction at the Interface

In quasi- and one-dimensional models, an important value is the components’ mass fraction at the interface between the liquid and bulk gas phase. In this regard, many researchers assume the vapor-liquid equilibrium, while it has been shown that this assumption is not necessarily correct, especially in the case of small droplet size, high temperature, and high convective regimes [216]. Consequently, although the intensified and highly convective regimes are desirable, they can complicate the modeling of this phenomenon. Hence, to increase the precision of the model, the effect of non-equilibrium conditions can be taken into account for the intensified processes or in the practical conditions [217].

##### Non-Ideal Behavior

In calculating the equilibrium (or non-equilibrium) composition at the interface, the deviation from the ideal situation [218] of the liquid droplet is an important parameter. Usually, the equilibrium concentration of the components at the interface is calculated via the ideal Rault’s law. However, to increase the reliability of the model, the modified Rault’s law by considering the activity coefficients of the components should be taken into account [219]. These activity coefficients can be calculated via the UNIFAC approach [220]. This way, it is possible to account for the size and shape of the molecules as well as the interaction between the functional groups, which are important parameters for the cracked molecules in the liquid phase during the pyrolysis of PW. As the result of non-ideality in the droplet, the concentration gradient is observed in the multi-component liquid droplet. However, by evaporation of the molecules with larger activity coefficients, the non-ideality decreases, and a more homogeneous concentration distribution of components in the liquid droplet is obtained in the later stages of the evaporation process [219]. It is worth mentioning that non-ideal effects at low temperatures are typically more significant than at high temperatures [219]. Hence, in the case of gasification with high temperature and low pressures, it is possible to neglect the effect of non-ideality [172] if the computational costs are a constraint. 

##### Role of Radiation

At high temperatures, radiation plays a significant role to the extent that, when considering the radiation contribution in a simulation, by increasing the temperature from 503 to 703 K, the time for converting the multi-component hydrocarbon liquid droplet to the gas phase decreases by 48% [219]. This contribution is incorporated in the effective enthalpy of evaporation and the heat balance equation of the droplet [219]. Hence, it is expected that this phenomenon is much faster in gasification, compared to pyrolysis, because of the higher temperatures of the former.

##### Role of Surface Area

Another important parameter to correctly estimate the evaporation rate is the surface area, which is in fact crucial for the rate of all transport phenomena. In general, to account for the effect of surface area, in the case of solid particles, averaging the surface area of the particles that are being fed to the system can be a solution. However, for the systems taking into account liquid droplets, this is more complicated. This can be done in a simplified case by solving a transport equation if it is assumed that instead of one droplet, n droplets are present in the system. Subsequently, a transport equation for the number density per unit mass can be coupled to the other transport equations to account for the effect of the number of particles in the total surface area of a liquid. Furfaro et al. [221] implemented this approach to take into account the multiple droplet effect (instead of a single droplet) in the study of droplet evaporation. Finally, the coalescence and breakup of the droplets/bubbles and their size distribution can be also taken into account, which is more related to the reactor scale modeling and will be discussed more in Section 7.

#### 6.2.3. Simplifying Assumptions

In simulating the evaporation of multi-component fuels, there are some common simplifying assumptions in the literature that allow simulating the PWG at lower computational costs. These assumptions include but are not limited to:

1. Considering the liquid as a spherical droplet
2. The presence of an inert atmosphere
3. Negligible diffusion of the gas to the liquid
4. Negligible mass diffusion due to temperature and pressure gradients

Some of these simplifications can be observed by comparing parts a and b of Figure 5. However, one should be careful about applying these assumptions in the PWG. One of the most important ones is assuming non-reactive conditions [175,216]. If the goal is to simulate evaporation during the PWG, this assumption neglects the presence of a gasification agent and the gas phase reactions that change the composition of the gas in the vicinity of the liquid droplet. This can change the gas thermophysical properties and composition, which strongly changes the evaporation rate [172] and affects the concentration gradients.

Table 3 provides an overview of different simulation studies that are focused on the evaporation of multi-component liquids. This table includes both liquid droplets and film. The latter can be used in the falling film reactors or to simulate the possible liquid layers that are attached to the reactor walls. This table also includes whether the internal and external heat and mass transfer have been considered or not, which is helpful for Section 6.3.

Based on what is observed in Table 3, some important points can be concluded that are helpful in creating a simulation framework for PWG in particle scale:

1. The heaviest component that has been implemented in these simulations is C_20_. Although the table doesn’t cover all the available studies in this regard, it can demonstrate that in general, not all the components available in the liquid phase of PW during the pyrolysis have been assessed extensively. Hence, one of the main areas to be focused on is the assessment of the cases that, from the components’ point of view, are closer to what is happening in PWG.
2. The shape of the liquid phase is important in simulations. In each study, either spherical or film shape is assessed. This is while different shapes can be simultaneously present in PWG, e.g., it can be droplet, agglomerate, or the liquid film on the wall. Besides, in all cases in the table, a uniform characteristic length of the liquid phase is considered, while the shapes that are present in the PWG are not perfect spheres or liquid film. This demonstrates the complexity that is faced in PWG due to the shape imperfections.
3. Most of the cases consider the ideal gas assumptions and this can be true due to the high temperature and low pressure [172,219]. However, for the liquid phase, due to the presence of multiple components with different properties, this is not necessarily true. Implementing the non-ideal conditions for a large number of components is a challenge itself.
4. Many of the studies use the DMC approach. This demonstrates that the simulation of the evaporation in the PWG can also be done in this approach at a logical computational expense and hence, can be coupled to the available detailed kinetic models for the plastic pyrolysis.

As it was demonstrated in this part, similar to other parts, available simulation studies of evaporation are based on simplifying assumptions. Hence, they can be improved by removing those simplifying assumptions. Open-source platforms have been recently developed in this regard, such as OpenSMOKE++ [222] and DropletSMOKE++ [214], which can be used and developed further to incorporate the abovementioned items in the simulation framework of evaporation.

### 6.3. Interfacial Heat and Mass Transfer

The effect of interfacial heat and mass transfer in the simulation framework gains special importance in the case of scale-up, where the role of these phenomena becomes crucial [98]. Different approaches and correlations can be implemented in this regard. However, the main challenge would be choosing the best available (Nusselt and Sherwood) correlations for the transfer coefficients. Hence, the focus is not on the details of all the modeling approaches for the interfacial transport phenomena, but to assess the challenges that are related to using those correlations in the PWG process.

#### 6.3.1. Empirical-Based Correlations

The parameters used in various proposed correlations are obtained via empirical or numerical approaches [223]. The empirical approaches, which are the more classical ones, are extensively used in large-scale simulation frameworks [224,225,226]. To name a few: Ranz-Marshall [227] and Gunn [228] for the Nusselt correlations. The advantage of using these correlations is their wide application in the literature and their simple implementation in the numerical modeling framework of the PWG. However, the drawback of using these empirical correlations in the PWG is that they are based on simplified transfer models and developed for general purposes and idealized conditions [229]. Hence, their validity is questionable for complex reactive flows. This can be inferred from the observed differences between different correlations that are more recently developed for various simple fluid–solid systems (Table 4). Even if the goal is to derive the empirical correlation specific to the desired PWG system, another challenge would be the complexity of the experiment and process based on which the correlation should be derived. Besides, empirical closures should be simplified to provide information on the mean thermal dynamic characteristic of the system [223]. Consequently, considering the advances in numerical simulation methods, such as PR-DNS or LBM, precisely deriving the correlation parameters via numerical methods might be a safer approach for PWG.

#### 6.3.2. Numerical-Based Correlations

With the recent development and improvements in numerical resources, high-resolution numerical simulations, such as PR-DNS, can be used as virtual experiments to obtain the parameters for the interfacial heat and mass transfer correlations. An example of a robust simulation in this regard is the framework that has been developed by Hardy et al. [230]. They coupled the weakly compressible approximation and the Brinkman penalization method in a PR-DNS framework. Such a framework can be used as a base to develop correlations for interfacial heat and mass transfer in PWG since it was created specifically for reacting gas-solid flows.

Table 4 summarizes the recently numerically developed correlations for the Nusselt number [223]. It is important to notice different cases and conditions for which the correlations are derived:

1. Each of them is derived for a specific range of void fraction and Reynolds and Prandtl numbers
2. For the special case of gasification in supercritical water (which can be used for the PWG as well [231]) specific correlations have been developed [232,233,234]
3. For the particles with different shapes, the Nusselt correlations have been developed, including the incident angle of the particles [233]
4. Depending on the direction of heat flow, the Nusselt correlation is different, due to the different behavior of water properties in the heating and cooling process at supercritical conditions [234]

It is worth mentioning that this discussion was based on the heat transfer and a similar discussion can be made for the Sherwood number correlations that are implemented in the interfacial mass transfer, and hence, are not explained here. 

Considering the abovementioned facts, it can be concluded that even with precise numerical methods, for each specific condition, a different correlation should be derived to correctly reflect the heat and mass transfer behavior of that specific system. Consequently, to simulate the interfacial heat and mass transfer for PWG:

1. The application of the classical empirical correlations for the complex systems is in doubt because it has been shown that for each case, a different correlation (which has been validated against the experimental data) should be developed
2. The numerical tools have been advanced enough to be used for developing new correlations for each specific condition of PWG process. This way, it is possible to increase the precision of the interfacial heat and mass transfer models used in this process.

**Table 3:** Overview of simulation works for evaporation of multi-component mixtures.

Feedstock	Liquid Shape	Ideality	Spherical Droplet/Uniform Film Thickness	0D/1D	Internal Heat/Mass Transfer	External Heat/Mass Transfer	Reactive	Radiation	Equilibrium	Approach	Ref
H_2_O, CH_3_OH, C_2_H_5_OH, 1-C_4_H_9_OH, n-C_7_H_16_, n-C_10_H_22_	Droplet	Real fluid (UNIFAC), ideal gas	Yes	0D	No/No	No/No	No	No	Yes	DMC	[175]
C_2_H_5_OH, n-C_5_H_12_, cyclo-C_5_H_10_, 1-C_6_H_12_, n-C_7_H_16_, C_7_H_8_, iso-C_8_H_18_	Droplet	Real fluid (UNIFAC), Ideal mixture for the gas phase	Yes	1D	Yes/Yes	Yes/Yes	Yes	Yes	Yes	DMC	[172]
iso-C_6_H_14_, n-C_7_H_16_, iso-C_8_H_18_, cyclo-C_9_H_18_, n-C_10_H_22_, ben-C_10_H_14_, n-C_11_H_24_, n-C_12_H_26_, ben-C_12_H_18_, n-C_13_H_28_, n-C_14_H_30_, n-C_15_H_32_, n-C_16_H_34_, n-C_17_H_36_, n-C_18_H_38_, n-C_19_H_40_, n-C_20_H_42_, n-C_21_H_44_, n-C_22_H_46_, n-C_30_H_62_	Droplet	Real fluid, Real gas	Yes	1D	Yes/Yes	Yes/Yes	No	-	No	DMC	[216]
n-C_6_H_14_, n-C_7_H_16,_ iso-C_8_H_18,_ n-C_10_H_22_	Film	Ideal fluid, Ideal gas	Yes	1D	Yes/No	-	No	-	Yes	DMC	[176]
C_4_H_9_OH, C_7_H_8,_ n-C_10_H_22_	Droplet	Non-Ideal fluid (UNIFAC)	Yes	1D	Yes/Yes	Yes/Yes	No	Yes	Yes	DMC	[219]
n-C_7_H_16_, n-C_16_H_34_	Film	Ideal gas	Yes	1D	Yes/Yes (polynomial expressions)	Yes/Yes	No	-	Yes	DMC	[181]
C_10_H_22_, C_16_H_34_	Film	Ideal and Non-Ideal Gas	Yes	1D /Quasi-Dimensional	Yes/Yes (polynomial expressions)	Yes/Yes	No	-	Yes	DMC	[213]
C_7_H_16_, C_10_H_22_, C_16_H_34_	Droplet	Ideal Gas	Yes	1D	Yes/Yes	Yes/Yes	No	-	Yes	DMC	[173]
n-C_5_H_12_, iso-C_5_H_12_, C_7_H_16_, iso-C_8_H_18_, C_9_H_20_, C_10_H_22_, C_12_H_18_, C_12_H_26_, C_16_H_34_, C_20_H_42_	Droplet	Ideal and Non-Ideal Gas	Yes	1D /Quasi-Dimensional	Yes/Yes (polynomial expressions)	Yes/Yes	No	Yes	Yes	DMC	[209]
H_2_O, CH_3_OH, C_2_H_5_OH, C_3_H_6_O, C_4_H_9_OH, 3-C_5_H_10_O, C_8_H_18_, C_10_H_22_, C_12_H_26_, C_14_H_30_, C_16_H_34_	Droplet	-		1D	No/No	No (Isothermal)/Yes (Stefan-Maxwell approach)	No	-	-	DMC	[177]
Air, H_2_O	Droplet	Ideal Gas	Yes (Including the number of droplets)	1D	Yes/Yes	Yes/Yes	No	-	-	DMC	[221]
C_7_H_8_, tr-C_10_H_18_, C_12_H_26_, iso-C_16_H_34_	Droplet	Ideal/Real Gas/Liquid	Yes	0D	Yes/Yes	Yes/Yes	Yes	No	Yes	DMC	[235]
n-Paraffin, Iso-Paraffin, Cyclo-Paraffin, Aromatics, Olefin	Droplet	Real Fluid, Ideal Gas (Modified)	Yes	1D	Yes/No	Yes/Yes	No	No	Yes	DMC	[179]
C_2_H_6_O (DME), C_7_H_16_	Droplet	Real Fluid (UNIFAC), Ideal Gas	-	0D	-	-	No	-	No (LK)	DMC	[217]
C_2_H_5_OH, iso-C_5_H_12_, iso-C_6_H_14_, iso-C_7_H_16_, iso-C_8_H_18_, C_9_H_20_, C_10_H_22_, C_12_H_26_	Droplet	Real Fluid (Wilson equation), Ideal Gas	Yes	1D	Yes/Yes	Yes/Yes	No	No	Yes	DMC	[182]
C_7_H_16_, C_10_H_22_	Droplet	Real/Ideal Gas	Yes	1D	No/No	Yes/Yes	No	No	Yes	DMC	[178]
iso-C_5_H_12_, iso-C_6_H_14_, iso-C_7_H_16_, C_7_H_8_, iso-C_8_H_18_, C_9_H_20_, C_10_H_22_, C_12_H_26_, C_14_H_30_, C_16_H_32_, C_18_H_34_	Droplet	Ideal Fluid	Yes	1D (Implemented in multi-dimensional CFD)	Yes/No	Yes/Yes	No	No	Yes	DMC (Derived from CMC)	[236]

Although numerical simulations such as PR-DNS are performed with high resolution, they are also limited because they are usually used for changing a few dimensionless numbers simultaneously and in a limited range [229]. The challenge in this field is how to deal with high values of the Prandtl and Schmidt numbers, which can occur in real systems. This causes a decrease in the size of the heat and mass boundary layers and imposes the necessity of higher resolution simulations, resulting in high computational costs. Moreover, coupling transport phenomena, the presence of multi-component systems (especially for the mass transfer), the presence of non-spherical particles, and experimental validation of the results are other difficulties in this regard [229]. 

To partially overcome the abovementioned challenges, an opportunity lies in merging different correlations that are developed for a specific range of applicability. Zhu et al. [223] have proposed a Nusselt/Sherwood correlation based on 145 PR-DNS results from six different references to be used in a wider range of conditions. Another goal of this correlation is to reflect the effect of coupled-transport phenomena including the effect of reactions. This was done by considering two extreme cases of Damköhler number (Da), which is 1 for the slow reactions and infinity for the extremely fast reactions. These correlations are derived for the particle-fluid system, performing relatively well for the non-spherical particles, for a range of 0.35 to 1 for the ε and 0 to 550 for Re. This approach shed a light on the importance and necessity of the machine learning application for the multi-scale modeling [55] of PWG, due to the presence of different regimes, conditions, and reactions that are observed in different locations of the reactor.

The focus of the discussions above was based on the gas-solid-only assumption. However, the same challenges and opportunities are expected for the situations in which the presence of liquid is considered, e.g., for the molten plastic droplet [237,238], the fluidization agents covered by the molten plastic layer [45], and the falling film reactors [67,169,239].

#### 6.3.3. Determining the Limiting Step 

As was demonstrated (Figure 5 and Figure 6), the sequence of chemical and physical phenomena occurring from molecular to reactor scales determines the overall performance of gasification. Dimensionless numbers are great tools in determining the limiting steps in this sequence. The first important one is the Biot number, which determines the dominant heat transfer mechanism by comparing the internal and external heat transfer, as:

(4) $$Bi=\frac{h\cdot L}{\lambda }.$$

where Bi is the Biot number, h is the convective heat transfer coefficient, L is the characteristic length, and λ is the thermal conductivity.

Hence, for large Biot numbers, the limiting heat transfer mechanism is the internal one. In this case, it is possible to neglect the interfacial heat and mass transfer limitations. The other two important dimensionless numbers are pyrolysis numbers, proposed by Pyle and Zaror [240], which are defined as:

(5) $$Py^{I}=\frac{\lambda }{\rho \cdot c_{p}\cdot L^{2}\cdot k},$$

(6) $$Py^{II}=\frac{h}{\rho \cdot c_{p}\cdot L\cdot k}.$$

In Equations (5) and (6), λ is the thermal conductivity, h is the convective heat transfer coefficient, ρ is density, $c_{p}$ is the specific heat capacity, L is the characteristic length, and k is the reaction kinetic constant. $Py^{I}$, the first pyrolysis number, defines the relationship between the rate of internal heat transfer and the reaction rate. $Py^{II}$, the second pyrolysis number, defines the relationship between the rate of external heat transfer and the reaction rate. In cases that Bi≫1, i.e., the internal resistances dominate the heat transfer rate, the competition is between the internal limitations and the reaction kinetics. In this case, the first pyrolysis number (Equation (5)) is utilized since it compares the velocity of the temperature front and the reactions taking place. Hence, for large $Py^{I}$ number, the internal transfer limitations are not the impediment to the pyrolysis reactions. This situation is expected to be observed in the cases of having a small characteristic length. Otherwise, in falling film technologies or the conventional FB in which a large characteristic length of the molten phase presents, the internal transport limitations can become an important and limiting step. On the other hand, for Bi≪1, the external resistances are the limiting steps for heat transfer. In that case, the second pyrolysis number (Equation (6)), which is also called the external pyrolysis number [240], determines the dominancy of the kinetics versus the external heat transfer. This number is derived from the multiplication of the first pyrolysis number and the Biot number to change the scenario from the internal limits to the external limits. Similar to the first pyrolysis number, in the case of a large external pyrolysis number, the process is controlled by the pyrolysis kinetic.

Different references [98,240,241] have used these dimensionless numbers to compare the importance of different steps. Nevertheless, determining k, which is the rate constant, is not clear. In the main reference that has introduced the pyrolysis number [240], this constant is defined as the “(first order) velocity constant for the intrinsic pyrolysis reaction”. Considering the discussions and complexities of the kinetic modeling of the plastic pyrolysis in Section 4, if the rate constant of a one-step first-order reaction is used for pyrolysis, it can not be representative of the intrinsic kinetics of pyrolysis reactions. Hence, these numbers are expected to be useful only in the engineering and global assessment of the process and not the detailed multi-scale modeling approaches. Thus detailed numerical modeling of particle scale, even in 1D, can help in determining the limiting step for the desired PWG operation and conditions. This way it is possible to minimize the computational cost of the multi-scale modeling of such a process, by neglecting the faster steps in the simulation framework.

### 6.4. Momentum Transfer

The momentum transfer is also important in determining the overall performance of the PWG on the particle scale. External convective fluxes and momentum transfer can alter the internal heat and mass transfer inside the molten phase [173], due to their possible effects on the internal circulation patterns. More importantly, they can determine the flow regime in the multi-phase system in the reactor scale, as will be explained in Section 7.

For the case of a gas-solid system (e.g., for char gasification, solid plastic before melting, or gas-solid-only assumption), the effect of the momentum transfer is only on the flow pattern in the reactor scale. The role of momentum transfer becomes more notable if the PWG simulation framework includes the liquid phase, since it also has an impact on the internal heat and mass transfer, in addition to the flow behavior, due to:

1. The Marangoni effects [210] and internal circulation flow inside the liquid [51,90,173]
2. The role that it plays in the interaction between the particle/droplet/bubbles and change in the interfacial area and shapes as the result of agglomeration, coalescence, and breakup

Hence, it is crucially important to precisely include momentum transfer in the simulations.

Different mechanisms contribute to the momentum transfer between phases and the forces exerted on each phase, the most important of which are the drag, lift, virtual mass [242], and buoyancy forces [243]:

1. The drag force is the main contributing force in the momentum transfer, which acts against the fluid flow direction to resist the motion of a particle, droplet, or bubble. This force is a function of fluid density, dispersed phase diameter, the slip velocity (difference between the velocity of the continuous and discrete phase), and a drag coefficient.
2. The lift force acts perpendicular to the flow direction and is the result of turning of the fluid because of the presence of the discrete phase.
3. The virtual mass force is the result of acceleration of the discrete phase, i.e., change of its relative motion compared to the fluid phase. This imposes an extra force as an extra mass or “added mass” in the acceleration force.
4. The buoyancy force acts against the gravity force as the result of the difference between the density of the fluid and the discrete phase

**Figure 13:**
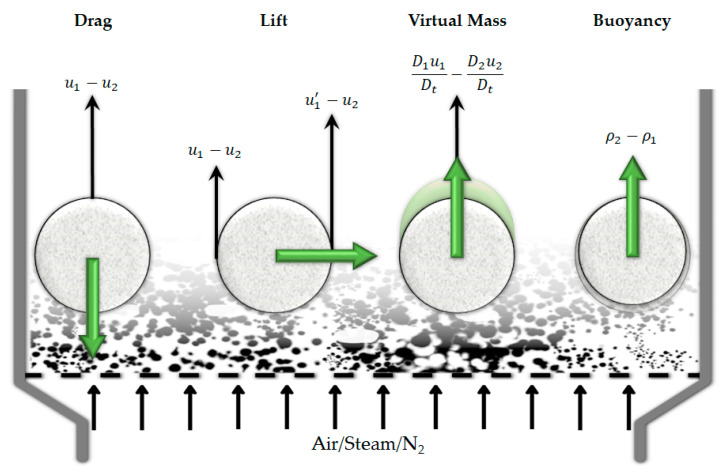
Schematic representation of different forces that contribute to the momentum transfer between phases (adapted from [263]). Indices 1 and 2 are related to the primary/fluid phase and the discrete phase, respectively.

The schematic of these forces is shown in Figure 13. This figure is based on the gas-solid fluidized bed reactor. However, similar forces act on the discrete phase in all systems, i.e., on solids, droplets, or bubbles.

#### 6.4.1. Drag Force

In the case of the gas-solid-only assumption, drag is the most contributing force in the momentum transfer. Similar to the interfacial heat and mass transfer, several correlations are available in the literature for the drag coefficient [242,244,245]. The frequently used ones are Gidaspow [246], Syamlal O’Brien [247], and energy minimization multiscale (EMMS) [248], to name a few. The challenge in PWG, however, is correctly implementing the drag force. The size of the particle is important because it accounts for the surface area that causes the drag. Hence, if the shape of the particle is not a perfect sphere, the drag force can be two to three times larger than the perfect sphere conditions [245]. Consequently, for PW feedstocks, which are not necessarily perfect spherical particles/droplets, special care should be taken. This, in situations where the liquid characteristic length is not small enough to be neglected, becomes more critical. This is because of the dynamic changes and deformations in the shape of particles and droplets, as well as their possible coalescence and break-up. Otherwise, similar to gas-solid systems, different drag correlations for the gas-liquid systems [242,249,250] can also be used to define the momentum transfer between the gas and liquid phases.

To deal with the non-sphericity, several correlations for the drag coefficient have been developed [244] in which a shape dependant parameter is incorporated. This parameter can include sphericity, circularity, aspect ratio, flatness, and elongation, among others [251]. In most of those correlations, the sphericity parameter is used to account for the shape non-idealities, and this has been the recommended shape factor for the microplastics as well [251]. This parameter is defined as the ratio of the surface area of a volume equivalent sphere to the actual surface area:

(7) $$\phi =\frac{A_{Sphere}}{A_{Actual}}.$$

where ϕ is the sphericity parameter and A is the surface area.

In the most simplified approach to taking the non-sphericity into account, the particle size is multiplied by the sphericity parameter [252]. Thus, the effect of shape non-idealities appears directly in the drag force, and not the drag coefficient. As a result, the drag force always increases for the discrete phase with a sphericity of less than 1. In more precise and complicated approaches, the sphericity parameter is used in the correlations of the drag coefficient and this can impose a non-linear behavior of the drag force versus the sphericity parameter [253,254]. 

**Table 4:** Recently developed Nusselt correlations via numerical methods for the particle-fluid systems (Adopted from Ref. [223]). Reprinted from Chemical Engineering Journal, Vol. 374, Li-Tao Zhu, Yuan-Xing Liu, Zheng-Hong Luo, An enhanced correlation for gas-particle heat and mass transfer in packed and fluidized bed reactors, Pages No. 531–544, Copyright (2019), with permission from Elsevier.

Correlation	Method	Limit				Year	Ref
		ε	Re	Pr	**Shape/Conditions**		
$Nu=\left(7-10\varepsilon +5\varepsilon^{2}\right)\left(1+0.1Re^{0.2}Pr^{1/3}\right)+\left(1.33-2.19\varepsilon +1.15\varepsilon^{2}\right)Re^{0.7}Pr^{1/3}$	DNS	0.4–0.9	10–100	1.0	Spherical	2014	[255]
$Nu=\left(-0.46+1.77\varepsilon +0.69\varepsilon^{2}\right)/\varepsilon^{3}+\left(1.37-2.4\varepsilon +1.2\varepsilon^{2}\right)Re^{0.7}Pr^{1/3}$	PR-DNS	0.5–0.9	1–100	0.7	Spherical	2015	[256]
$Nu=2.67\left(\pm 1.48\right)+0.53Re^{0.77}Pr^{0.53}$	PR-DNS	0.351–0.367	9–180	0.5–1.0	Spherical	2017	[257]
$Nu=1.77\left(\pm 1.39\right)+0.29\varepsilon^{0.81}Re^{0.73}Pr^{0.5}$	PR-DNS	0.418–0.526	9–180	0.5–1.0	Cylindrical	2017	[258]
$Nu=\left(1.49-0.88\varepsilon +0.078\varepsilon^{2}\right)\left(2.458-0.042Re^{1.09}Pr^{1/3}\right)+\left(1.114-0.62\varepsilon -0.08\varepsilon^{2}\right)Re^{0.7}Pr^{1/3}$	PR-DNS	0.65–0.9	10–200	0.74	Ellipsoidal	2017	[259]
$Nu=\left(8.35-7.4\varepsilon \right)\left(1-0.11Re^{0.2}Pr^{1/3}\right)+\left(3.92-7.67\varepsilon +3.96\varepsilon^{2}\right)Re^{0.7}Pr^{1/3}$	DNS	0.877–0.948	0–550	1	Cellular porous media	2018	[260]
$Nu=\left(2+0.77\varepsilon +0.64\varepsilon^{2}\right)+\left(0.6+1.1\varepsilon \right)Re^{0.5}Pr^{1/3}$	LBM	0.5–0.9	1–100	0.7	Sphere	2019	[261]
$Nu=\left(3.2846-5.1844\varepsilon +3.1741\varepsilon^{2}\right)\left(1+0.7Re^{7.219e^{-8}}Pr^{1.0663}\right)+\left(1.3715-1.3531\varepsilon +0.334\varepsilon^{2}\right)Re^{0.5939}Pr^{0.328}$	DNS-LBM	0.6–1.0	20–500	0.5–1.5	Sphere	2019	[262]
$Nu=0.3832Re^{2/3}Pr^{1/3}Ar^{-0.2456}-0.0641Re^{1/2}Pr^{1/3}Ar^{0.2411}+5.1188Ar^{0.0452}$	PR-DNS	-	10–200	3.07	Spheroid (*Ar* = 0.5–2.5)/SCW	2019	[232]
$Nu=0.3695Re^{2/3}Pr^{1/3}Ar^{-0.2761}-0.0387Re^{1/2}Pr^{1/3}Ar^{-0.6632}+5.2154Ar^{0.0254}+Ar^{-0.5561}\left(Ar-1\right)0.153Re^{0.6989}\sin^{2}\left(\frac{1.1187\theta \pi }{180}\right)$	PR-DNS	-	10–200	0.744, 3.07	Spheroid (*Ar* = 0.5–2.5)/SCW	2019	[233]
$Nu=Nu_{0}{\left(\frac{\rho_{in}}{\rho_{p}}\right)}^{-0.718}{\left(\frac{c_{p_{in}}}{{\bar{c}}_{p_{p}}}\right)}^{0.33}{\left(\frac{\lambda_{in}}{\lambda_{p}}\right)}^{-0.4}$ $Nu_{0}=2+Pr^{0.4}\left(0.4Re^{1/2}+0.06Re^{2/3}\right){\left(\mu_{in}/\mu_{p}\right)}^{0.25}$	PR-DNS	-	10–200	0.7–380	Spherical/SCW/Cold particle	2020	[234]

Besides the sphericity parameter, other shape-dependant parameters have also been implemented in the drag coefficient correlations to increase the precision of the drag force predictions. The reason lies in the fact that the sphericity parameter (ϕ) can not always reflect the shape non-ideality effects and the particle orientation. Moreover, for some situations such as spheroids in the Stokes region or lengthwise plates at high Re numbers, smaller drag forces have been recorded [264]. This is while the sphericity-parameter-based correlations (such as Haider and Levenspiel [254]), predict a larger drag coefficient for these situations, due to the decrease in their sphericity [264]. As an example of a more precise correlation for special shapes, Holzer et al. [264] used two different shape factors, called crosswise and lengthwise sphericity. In another, more frequently used approach, Dioguardi et al. [265] used a different definition of the shape factor (ψ),which is defined by Dellino et al. [266]:

(8) $$\psi =\frac{\phi }{\chi }$$

as the ratio between the sphericity parameter (ϕ) and the circularity (χ). The circularity is defined as the ratio of two different perimeters by Equation (9):

(9) $$\chi =\frac{P_{mp}}{P_{c}}$$

$P_{mp}$ is the maximum projection perimeter and $P_{c}$ is the perimeter of the circle equivalent to the maximum projection area of a particle. Using this shape factor, Dioguradi et al. [265] developed a correlation for the drag coefficient, which is validated against the spherical and non-spherical particles. This can be useful in the case of having a range of different shapes of particles, and hence, is practical for the PW feedstock, which was proved by Van Melkebeke et al. [251] as well.

Although its application is different from the gasification process, the research done by Van Melkebeke et al. [251] could be of great help, which assesses different shape-dependent drag models on the sinking behavior of micro-plastics. A wide range of particles size (0.63 to 3.48 mm), plastic types (HDPE, PET, PP, PS, PE, and PVC), and shape factor (sphericity from 0.04 to 0.97) were studied in their work. They declared that the recent non-spherical drag model of Dioguardi et al. [265] shows the best performance to study the microplastic potential remediation techniques. This demonstrates the extensive work that should be done to only validate a drag model for a specific case, e.g., in this case, the sinking behavior of microplastics. 

Non-sphericity is not the only factor that impacts the drag force. It has been proven by Zhang et al. [267] that the drag force of a particle in the presence of homogeneous and heterogeneous reactions is different from a simple particle with outflow in non-reactive conditions. This is supposed to be more pronounced in the gasification process compared to pyrolysis, due to subsequent homogeneous and heterogeneous gasification reactions around the particles/droplets (Figure 5).

High-resolution numerical simulations can be done to study the effect of a combination of different affecting parameters, such as the shape, temperature, and chemical reactions. To the best of the authors’ knowledge, this has not been done yet for the PWG or similar processes. However, they have been done in separate studies. As was mentioned above, Zhang et al. [267] studied the drag force for a burning particle using particle-resolved simulations and concluded that the drag force of a reactive particle is higher than the non-reactive one. They declared that the changes in the flow pattern (because of the Stefan flows prompted by the heterogeneous reactions), as well as the recirculation wake, will change the drag force. 

In another study, Sanjeevi et al. [268] performed a direct numerical simulation using the Lattice-Boltzmann method to develop a correlation for the drag coefficient of non-spherical particles, which accounts for their incident angle. Nonetheless, this comes with a high computational cost. Moreover, the computational cost increases when having different shape factors (which is usually the case for real-world PW). So, different classes of particles should be defined in the simulation framework. 

#### 6.4.2. Non-Drag Forces

Besides the drag force, other forces can also be important in the momentum transfer between phases. However, this depends on the different situations in which the PWG is being operated and how precise the simulation results should be. Papadikis et al. have taken into account the buoyancy and virtual mass forces and neglected the lift force in modeling a fast pyrolysis process in a FB reactor [243,269]. On the other hand, Armstrong et al. have reported that the lift and virtual mass force are neglected in a gasifier simulation by indicating that the lift force is highlighted only in the case of large particles [270]. This could be important in the case of PWG since if the PW is not pretreated well, nonuniformity or large sizes of particles are present in the system [251]. 

If the presence of a liquid phase is considered, the role of other forces in the momentum transfer between the phases depends on the operation. For the falling film systems, only the drag force is important and other forces are not usually assessed [169,239,271]. However, non-drag forces become more important in the case of the presence of liquid in the system [242] for the bubbly or droplet regimes. In any case, the drag force is still the most important while the importance of the lift force is still questionable [242] and the significance of the virtual mass force is only in the regimes with high-frequency fluctuations of the slip velocity [272]. Consequently, to the best of the authors’ knowledge, there are no clear criteria to determine the importance of other non-drag forces [273].

Hence, for the case of PWG, as far as the availability of the computational resources is not a concern, a conservative approach is to include the non-drag forces, since they increase the precision. As an example, Duguay et al. [274] assessed different interfacial momentum transfer models in a bubble-induced recirculatory flow. They demonstrated that neglecting the lateral lift force could end up in unreliable predictions of the system behavior, such as a 40% underestimation of volumetric fluid flux through a specific plane. Consequently, this should be assessed for each case individually to determine the importance of the non-drag forces. It is worth mentioning that, the complexities that were discussed for determining the drag force in the PWG are valid for the non-drag forces as well.

## 7. Multi-Phase Flow Modeling

One of the most crucial parts of the PWG process in a multi-scale framework is the multi-phase flow modeling of this process inside the reactor. This is because it determines the effect of the flow behavior and reactor scale phenomena on the process outcome and includes many different parameters. Some small changes in the design of a plastic gasifier can change the product distribution a lot, which can be due to the velocity profile, but also the temperature profile. Yamamoto et al. have reported that the optimized location of oxygen blowing could minimize the volume of produced dioxins [275]. A similar effect can be seen in the reduction of tar production as the result of the location of injecting the feedstock in a co-gasification process [276,277].

The performance of multi-phase flow PWG reactors is determined by the flow pattern and reaction kinetics, as well as all other parameters that were discussed in this review. The flow pattern determines the residence time, and this parameter, together with the kinetics and transport phenomena, determines the product distribution. To obtain the flow pattern behavior in the simulations, the effects of turbulence, interactions with the walls or reactor internals, and phase interactions can be accounted for, either by explicitly calculating these phenomena or through correlations, referred to as closure models. In this regard, time scales of chemistry, mass, momentum, and heat transfer play important roles. If they are different enough to make one of them the limiting step, one can simplify the model (e.g., consider only chemistry or only mass transfer) without losing much precision. Otherwise, the effects of different phenomena at different scales should be coupled to the hydrodynamics—and the interaction between phases and with the reactor walls and internals at different locations of the reactor—to predict the overall outcome of the process. Consequently, different approaches and methods have been devised with different levels of detail, complexity, and of course, computational costs to simulate a process at a reactor scale. In this section, the focus is not on the details of all the reactor modeling approaches but is on how different approaches can handle the phenomena and facts that are specific to the PWG.

The multi-phase flow simulation of PWG [29] or similar processes, such as municipal solid waste gasification [278,279,280], is usually based on coal or biomass gasification, while PWG is different from conventional solid fuel gasification and more complex, due to the presence of molten plastic [38]. This can impose a different behavior between particles/droplets due to their stickiness [281]. Hence, they may cause coalescence and breakup [282], aggregation in presence of fluidizing agent [282], or change their elastic behavior [283]. The same holds for the particle/droplet–wall interaction and may end up forming a layer of molten plastic on the reactor wall, similar to the slag layers in the coal gasification process [284]. A similar phenomenon is present in the thermochemical recycling of PW due to the presence of impurities in the waste streams [285]. Besides, due to the same reason, it is possible that the flow regime changes in some areas of the reactor, and the dispersed phase changes from the solid/liquid phase to the gas phase, and vice versa [286]. This is while the application of some models such as the drag model is valid based on a fixed dispersed phase, such as the Gidaspow drag model [246]. Finally, the molten plastics behave as a non-Newtonian liquid [187,188], which affects the flow pattern in the reactor.

Besides neglecting the presence of molten plastic, some other simplifying assumptions, which are also used in the simulation of coal and biomass gasification, can make the simulation results unreliable. One of the important assumptions is neglecting the particle size distribution, which can affect the: flow pattern, the particle-particle, particle-wall, and inter-phase interactions. Even if it is assumed that the feed is monodispersed, the shape and size of the particles change during the pyrolysis and gasification, and hence, their interactions with each other, the wall, and the gas phase may change. Other than that, the simulations are usually done assuming the solid fuel as a perfect soft-surface sphere, while this is not the case in reality.

In this section, it is tried to provide guidelines that can be implemented to solve the abovementioned challenges for the PWG process. However, first, it is necessary to provide short explanations of different modeling approaches together with simulation works that have used them for the PWG (and in some cases, similar processes).

### 7.1. Reactor Modeling Approaches

**Figure 14:**
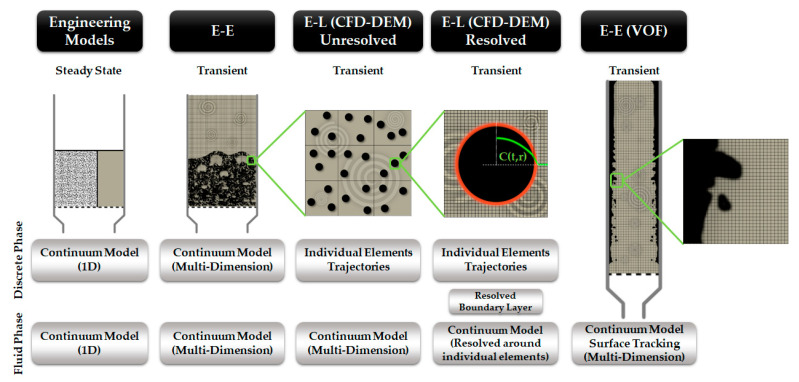
Illustration of different multi-phase flow modeling approaches for the fluidized bed technology (adapted from [169,288]).

Reactor modeling approaches can be divided into two main categories: the engineering models and the 3D CFD models. Figure 14 illustrates these approaches, which are briefly explained in the next sections. Since the current state-of-the-art main technology of gasification is the FB reactor, the main focus of this section is on this technology as well.

#### 7.1.1. Complex vs. Ideal Models

Besides the engineering or CFD models that are described in the subsections below, it is worth mentioning that in the initial phases of the study, it should be assessed and decided if using a complex model is necessary or not. First, the degree of complexity of different models in a multi-phase flow should be compatible with each other. Bal and Rein have demonstrated that to have a reliable model for the pyrolysis of non-charing polymers, if a simple heat transfer model is used, implementing the complex kinetic model is not justified. The reason is that the degree of complexity of the heat transfer model substantially affects the final results [287]. If the surface temperature of the polymer is not predicted correctly, the most comprehensive kinetic models won’t result in correct product distribution. 

Secondly, it should be analyzed under what conditions ideal-flow models—i.e., batch, continuous-stirred-tank, and plug-flow-reactor—can be used and when the flow pattern has to be calculated in detail in three dimensions. As was mentioned before, this depends on how the time scales of different phenomena at different locations compare. As an example, if the limiting step in the heat transfer is in the reactor wall compared to the radial heat transfer, then a 1D model with a closure model for the wall heat transfer coefficient would suffice to predict the desired outcome compared to a 2D or 3D model. Considering the mass transfer, if in a system the mixing is perfect, then a CSTR model would result in the desired outcome. On the other hand, if there is no perfect mixing and back-diffusion compared to the convection, then the Péclet number is high and a PFR model can be considered for the system. However, determining these regimes, especially for complex processes and reactor configurations is not straightforward.

Experimental techniques can be used to assess the flow regime and determine whether it can be a candidate to be modeled as an ideal CSTR or PFR, by determining the residence time distribution [289] or degree of mixing [290]. The challenge associated with the experimental approaches is that for every configuration, particle type, and flow condition, the experiment should be repeated. Consequently, another alternative that can be less expensive is using numerical methods.

**Figure 15:**
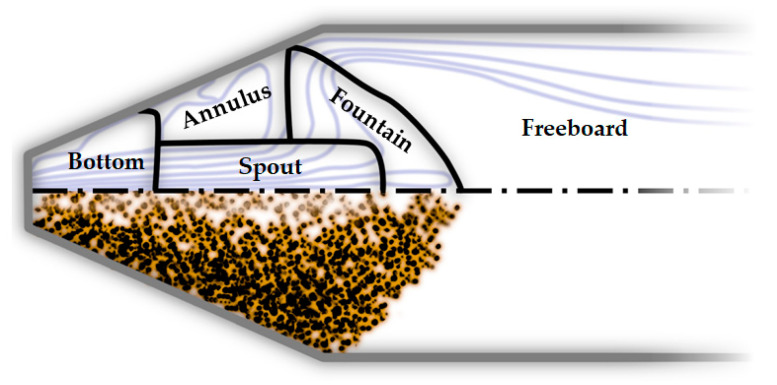
Schematic of the spouted bed reactor divided into five zones, based on the streamlines and flow regimes obtained by the CFD simulations to be used in the ERN model. Reprinted (adapted) with permission from Ref. [61]. Copyright 2014 American Chemical Society.

Simple non-reactive CFD simulations can be used in this regard. If the simulation results show ideal behavior, an ideal reactor model can be used to implement the kinetic models. In a more advanced version of this approach, it is possible to mimic the system behavior by considering ideal reactor models for different areas. This can be done via Equivalent Reactor Network (ERN) generation. In these approaches, based on the flow behavior in different zones of the reactor, it is divided into a network of simplified ideal reactors and then the reactive simulations are done for that network to decrease the computational costs. Following this approach, Du et al. [61] created an ERN of a spouted bed reactor for air gasification of PE (Figure 15). They divided their reactor into CSTR, PFR, and equilibrium reactor models (Section 4.1.1), using the auto-zoning technique [291,292]. If these simplifications cannot be done and the time scales overlap, more advanced reactor models should be used, which are described briefly in the next subsections.

#### 7.1.2. Engineering Models

In the simplest approach of simulating the multi-phase flow within a non-ideal reactor model, the engineering models are usually developed in 1D (the axial direction—see Figure 14), which are justified by the fact that there are no radial temperature gradients inside the reactor, except in the near-wall region (high radial thermal conductivity). Using these models, it is possible to capture the transport phenomena between different phases at a low computational cost and without the need to solve the Navier-Stokes equations.

Different types of fluidized beds can be modeled via this approach. One of the methods in this approach is the bubbling flow model, which is usually referred to as the “two-phase flow model” [293,294]. In this method, the reactor is considered to operate in two different phases (Figure 14). One phase, which is called the bubble phase (or low-density phase), is composed of the gas phase. Bubbles are in contact—through their surface—with the second phase, which is called emulsion (or high-density phase) and includes the mixture of gas and solid. The bubble phase is considered to be in plug flow, while for the emulsion phase, axial dispersion is introduced in the model to account for the back-mixing of the particles in the bed. Homogeneous reactions take place in the bubble phase and both homogeneous and heterogeneous reactions are assumed to occur in the emulsion phase. This approach has been the most utilized model for FBs [122] in engineering applications and was used widely in the modeling of coal [295,296] or biomass [297,298] pyrolysis and gasification in FB reactors. Hence, for the PWG with the gas-solid-only assumption, these research studies can be of great help. Table 5 shows a number of research studies that implemented this approach for gasification of PW, as well as coal and biomass.

To summarize the engineering models section, it is worth reviewing the literature in this regard. Slapak et al. utilized a two-phase model (including the kinetics of carbon steam gasification) to determine the minimum height of the fluidized bed for the steam gasification process for PVC waste [299]. No further details are provided by the authors regarding the two-phase model and its results. In the other work, Martínez-Lera and Pallarés Ranz [122] developed a modified two-phase model for the polyolefin gasification. They considered LDPE (Low-Density Polyethylene) as the reference feedstock. Their model includes three main parts: hydrodynamics, pyrolysis, and homogeneous reactions. For hydrodynamics, an important issue associated with the two-phase model is the production of a large amount of gas in the emulsion phase compared to the circulating gas [122]. This affects the void fraction. To deal with this problem, they implemented the method of De Souza-Santos [300], which takes into account the effect of generated gas via implementing an expansion factor, to increase the precision of the emulsion phase void fraction in different locations of the reactor. Further details about different correlations in this regard can be found in the review article by Gómez-Barea and Leckner [301]. For the pyrolysis model, they assumed it as instantaneous, and for the homogeneous reactions, they used a global mechanism of 18 reactions.

From Table 5 it can also be noticed that many of the large-scale reactors are modeled using this engineering approach. Moreover, they are mainly developed using in-house code. These two demonstrate the simplicity and lower computational costs of the two-phase model compared to the more complex simulation frameworks such as CFD.

Finally, it is worth mentioning that for the molten plastic gasification in a bubbly flow regime, some similar approaches can also be used to save computational costs. Due to the high viscosity of the molten plastics, which makes their mixing imperfect, the ideal models can not be used. According to Degaleesan et al. [302], the difference between the reactor sizes, using the ideally fully mixed and non-perfectly mixed liquid, can be up to 20 times. Hence, to be able to capture this phenomenon while avoiding the high computational costs of CFD simulations, some simplified reactor models, the so-called compartment models, can be used which are similar to creating the ERN model [61] (which was described earlier). Consequently, compartmentalization can also be done based on the prior knowledge about the flow regime, obtained via, e.g., CFD simulations [303]. The concept of this approach is to divide the reactor into different compartments, each of them as plug flow, axial dispersion model, or ideally mixed reactors. After dividing the reactor into different compartments, the desired transport equations between the compartments are solved or correlations are used to obtain the desired velocity, temperature, or concentration profile throughout the reactor [304,305]. The advantage of using this approach is that it is possible to apply the axial dispersion models [302] and coalescence and breakup of the bubbles [305], and calculate the gas hold up, bubble size, species concentrations, and diffusion and convection velocities, within different regions of the reactor.

#### 7.1.3. 3D Computational Fluid Dynamics

A more comprehensive technique to assess the process in the reactor scale when the time scales overlap and a high-resolution simulation of the reactor is needed, is the CFD simulation. This technique has been used since the 1960s. However, considering its high computational costs, it has gained much more attention only recently (based on the statistics from [16]) due to the higher available computational resources. It is worth clarifying at the beginning of this section that CFD, in this review, refers to both fluids-only and coupled fluid-discrete phases (solid particles/liquid droplets/bubbles). Moreover, the CFD approaches, such as DNS or PR-DNS, that are usually used to study the particle scale phenomena [229,232,234,255,256,262], such as internal and external transport phenomena, are not discussed in this section, but the examples of their application have been provided in Section 5.2 and Section 6.3. In the CFD simulations, mainly two different approaches can be used to deal with different phases: Eulerian-Eulerian (E-E) and Eulerian-Lagrangian (E-L). The general concept of these approaches together with their examples in the PWG or similar processes are discussed below.

##### Eulerian-Eulerian

In the E-E approach, the phases are assumed to be continuous interpenetrating flows of fluid elements. In this case, the presence of different phases in a cell is determined by the volume fraction and the conservation equations are solved for each phase by considering a common pressure for them as a common practice [306]. The equations of this approach are described in the Supplementary Information. To simulate the solid phase using this approach, the kinetic theory of granular flow (KTGF) [307] is used to close the solid-phase momentum equations [46], by using various empirical closure models [308,309]. Consequently, for the cases with the gas-solid-only assumption, the E-E simulation of the PWG is similar to the one for biomass or coal gasification.

If the PWG is going to be done by covering a layer of molten plastic on the fluidizing agent [45], the gas-solid-only assumption can also be helpful. However, the hydrodynamic coefficients, such as specularity and restitution—which reflect the particle–wall and particle–particle interactions, respectively—should be adjusted to account for the effects of the molten plastic layer. In other situations, the molten plastic can dominate the PWG and substantially affect the flow behavior. 

One of these situations is doing the PWG for a bulk of liquid (e.g., in the stirred reactors [68,69]) with the injection of a gasification agent—which is rarely reported. Bubbly flow CFD simulation studies can be helpful in such cases of PWG. In these systems, in addition to the drag force, other forces exerted on the bubbles, i.e., the lift, virtual mass, buoyancy, etc., should be taken into account. The other situation is the molten plastic droplet-only system, which is also rarely reported in the literature. As an example of this case, Yuan et al. [66] proposed a gas-liquid fluidized bed system with the goal of chlorine removal of PVC before pyrolysis. This system can be also simulated in an E-E framework. Bubbly flow and droplet regimes are associated with some challenges, such as bubbles/droplet coalescence and breakup, which are discussed later in this section.

To better capture the complexities associated with the presence of liquid in the system using an E-E framework, another approach is a special version of E-E, which is called the Volume of Fluid (VOF) method [249,271], in which the interface of the immiscible fluids can be tracked. In this approach, shared conservation equations are solved for phases in each cell. VOF is usually used for cases in which a relatively large interface between the gas and liquid is present. The simplified schematic of this approach has been illustrated in Figure 14. Due to the stickiness of the molten plastics and their tendency for coalescence and agglomeration (which makes relatively large clusters), a large interface between phases is created. Consequently, the VOF method can be a better approach to simulate these situations, e.g., for the rotary kiln pyrolysis [310]. A similar situation—i.e., large interface between the gas and liquid phase—is observed also in the falling film systems [67,169,311]. One of the drawbacks of this method is the required high-resolution spatial discretization to track the surface in the cells, which increases the computational costs. Developing solvers based on the GPU (Graphics Processing Unit) architecture [312] is a solution for this problem, which provides an opportunity to increase the precision and resolution of the simulations, and is discussed in more detail at the end of the section.

Table 6 provides more details on simulation studies of the PWG or similar processes using the E-E approach. As can be observed in this table, most of the E-E studies specific to PWG are non-reactive. Lee et al. have studied the hydrodynamic of a circulating fluidized bed reactor (CFB) to gasify the PW with circulating sand [313]. The goal of this study is to find the optimum condition—in this case, the gas superficial velocity and PW particle diameter—under which the solids create a uniform circulating flow. Although they determined the optimized values, they implemented simplifying assumptions such as the gas-solid-only system and mono-dispersed spherical particles. In another study, Du et al. [61] implemented an E-E approach to study the hydrodynamics of a spouted bed reactor for polyethylene air gasification to create the ERN for their reactive simulations. The details of this study have been discussed earlier in this section.

In a different approach and for the molten plastic, Yin et al. [311] have studied a falling film reactor—based on the PP pyrolysis—to assist with the design of this special type of reactor. They incorporated the evaporation model of the molten plastic in their simulations. It was illustrated that the evaporation behavior of the molten plastic on a hot surface is similar to water evaporation, and hence, the same methodology for simulation of water evaporation can be used in this reactor configuration. A very simplified lumped kinetic model was used for the PP pyrolysis. They used the VOF method to simulate the system’s hydrodynamics. One important aspect of their study is the heat transfer from the wall to the molten phase. To obtain the “effective” conductivity of the PP, they conducted an experiment that showed a strong dependence of this parameter on the temperature. They also further investigated, experimentally and numerically, the heat transfer characteristics of the molten plastic (PE, PP, PS, and their mixture) [67] for the same reactor configuration. To increase the precision of their simulation, in another research, they modified the VOF method to include the effect of the drag force [169]. They used the air-water system for the initial studies and then implemented the framework for the molten plastic pyrolysis.

An important parameter reported in Table 6 is the gas-phase residence time, which is crucial in determining the product distribution and tar formation [20]. However, as can be observed from this table, similar to the engineering approach, this value has not been reported in many of the E-E simulation works.

##### Eulerian-Langrangian 

In the E-L approach, the fluid phases are treated in the same way as in the E-E approach. However, to deal with the dispersed phase (solid particles, liquid droplets, or gas bubbles in a bubbly flow regime), they are tracked individually (the focus of this section) or simulated as a cluster of particles/droplets/bubbles. Their motion is governed by Newtonian equations and they are coupled to the continuous fluid phases to transfer momentum, heat, and mass. This, of course, comes at an additional computational cost but provides more accuracy compared to the E-E approach. The equations of this approach are also described in the Supplementary Information.

**Table 5:** Overview of fluidized bed gasification studies using two-phase modeling in engineering approach for different feedstocks.

Feed	Gasification Agent	Time (Reaction/ Space/Residence) (s)	Plant Size (OM-m^3^)	Bed Material	Temperature (°C)	Kinetic	Software/Code	Ref
Plastic (PVC)	Steam	-	Lab	Alumina	~900	-	Inhouse	[299]
Plastic (Poly Olefin)	Air-Steam	Pyrolysis: 0.02 Mixing: 5.4	Pilot (0.02 & 0.67)	-	700–850	Global	Inhouse	[122]
Coal, petcoke	Oxygen-Steam	-	Commercial (72)	-	1100 (Non-isothermal)	Global	Inhouse	[314]
Coal, limestone, inert material	Air-Steam-Carbon Dioxide	Devolatilization: <10	Pilot (0.07)	Limestone, Sand	600–1000	Global	Inhouse (FORTRAN)	[315]
Coal	Air-Steam	-	Lab & Pilot (2.6)	Dolomite	750–950 (Non-isothermal)	Global	Inhouse	[296]
Coal	Oxygen-Steam	Particle residence: 3600	-	-	700–900 (Isothermal)	Global	Inhouse	[295]
Biomass (Wood)	Air-Steam	-	Pilot (0.57)	-	900–950	Global	Inhouse	[297]
Biomass (Wood powder)	Air-Steam	-	-	-	700–900	Global	Inhouse (MATLAB)	[316]
Biomass (Straw)	Air-Steam	-	-	-	-	Equilibrium	Inhouse (FORTRAN)	[317]
Biomass (Sawdust)	Air	-	-	Sand	600–1600	Global	Inhouse	[318]
Biomass (Sawdust)	Air-Oxygen-Steam	Reaction: 140–3000	Pilot (0.06 & 2)	Ofite, Quartz & Silica Sand	700–900	Global	Inhouse	[319]
Biomass (Pine Sawdust, Rice husk)	Air-Steam	-	Lab (0.003) & Pilot (0.2)	-	665–900	Global	Inhouse (FORTRAN)	[298]
Biomass (Beech Wood)	Air-Steam	Gas residence time in the freebord: 2–4	Pilot (0.02)	Silica Sand	800–815	Global	Inhouse	[320]

The E-L approach is divided into two main categories: the unresolved and resolved approaches, which are illustrated in Figure 14. In the first approach, the fluid part is not spatially resolved around the discrete phase [321]. Hence, the cell-average quantities are applied to the particles present in the cell and the effect of particle motions on the flow behavior is determined based on their volume fraction [288]. The common practice in this approach is to use the cells that are at least three to five times larger than the particle size [322]. Consequently, the heat, mass, and momentum transfer between them is expressed by closure models [321] since the phase interactions are not resolved on the particle surface, but the average quantities are used.

The second approach is called the resolved CFD-DEM (Discrete Element Method) [321] (or DNS-DEM [288]). In this approach, the fluid phase grid is resolved around the particle surface, allowing to perform DNS. This method, which is called also Particle-Resolved DNS, as was discussed in Section 5.2 and Section 6.3, can be used to derive the closure models for the interfacial transport phenomena. The drawback of this approach is the high computational cost, in which small time-steps and non-dissipative discretization schemes are required and each individual particle should be tracked. This, for a reactive case, can result in an enormous number of equations (of the order of 1 × 10^7^–1 × 10^10^) that should be solved in each iteration for each time step.

For simulating the PWG with the gas-solid-only assumption in the E-L framework, the normal gas-solid simulations with the spring-dashpot-slider assumption [323]—to calculate the normal and tangential contact forces in the solid phase—can be implemented. However, to include the presence of molten plastic, the framework should be extended. The presence of liquid can affect the interaction between particles and also, between reactor walls and particles. The collision between the particle and particle and/or wall can result in attachment, and in some cases, rebounding (depending on the conditions, e.g., collision intensity, the cohesive forces, the velocity, liquid properties, etc. [324]).

For cases in which a relatively large interface between the gas and liquid is present, and the presence of a solid phase is also important, different approaches in the E-L method can be followed. According to Sun and Sakai [325], many of the conventional methods to simulate the solid particles in a gas-liquid-solid framework, such as CFD-DEM [326] or a combination of DEM and E-E [327], suffer from different sources of inaccuracy. These can include: using empirical closure models for fluid-bubble interactions, using different and separate drag models for continuum and disperse phase, and inconsistent fluid-particle interaction models, among others. To remove these limitations and improve the precision of simulating such a complicated case, they developed a new E-L framework (DEM-VOF) [325]. In their framework, the fluid phases are a continuum, separated by an interface. They are simulated by the VOF method and solid particles are simulated using the DEM particle tracking algorithm. The details of this approach can be found in [325,328,329]. 

An overview of some E-L CFD simulation studies, which can be helpful for the simulation of the PWG process is provided in Table 7. Among the CFD studies specific to the PWG, Janajreh et al. employed an E-L method to assess the feasibility of gasification for different PW types [29]. They simulated an entrained flow gasification in a drop tube reactor, in the presence of air, with particles of 134 μm in diameter. They run the simulations in an ANSYS Fluent environment and have validated their result against the experimental data of a drop tube reactor. To the best of the authors’ knowledge, except for the devolatilization kinetics, no other phenomena or models specific to the PWG (such as particle size change, melting, sticking of the particles, coalescence, and breakup) have been reported to be considered in this research.

**Table 6:** Overview of Eulerian-Eulerian simulation studies for different feedstocks and reactor designs that can be used as a guide in the PWG simulations.

Feed	Type	Gasification Agent/Process Gas	Gas Residence/ Space Time (s)	Plant Size (OM-m^3^)	Bed Material	Temperature (°C)	Phase	Software/Code	Ref
Plastic (Waste)	Circulating FB	Air	1–3	0.1 m^3^	Sand	-	GS	MFIX	[313]
Plastic (PE)	Conical Spouted Bed	Air-Steam	~3	Lab (0.001) Pilot (0.03)	Sand	800–900	GS	Fluent + Aspen Plus	[61]
Molten Plastics (mix PE, PP, and PS) ^1^	Falling Film	Nitrogen	-	Lab (0.002)	-	550–650	GL	OpenFOAM	[169]
Molten Plastic (PP) ^1^	Falling Film	Nitrogen	-	Lab (0.00004)	-	460–500	GL	Fluent	[311]
Molten Plastic (PE, PP, PS, mix) ^1^	Falling Film	Nitrogen	-	Lab (0.002)	-	550–625	GL	-	[67]
MSW, RDF	Plasma (Fixed Bed)	Air-Steam	-	-	-	~2200 (max)	GS	Inhouse (COMMENT)	[330]
MSW, Biomass (Coffee husk, Forest residues, Vines pruning)	Bubbling FB	Air-Steam	-	Semi-Industrial (0.8)	Dolomite (Experimentally)	500–1000	GS	Fluent	[278]
MSW	Bubbling FB	Steam	-	Semi-Industrial	-	850	GS	-	[331]
MSW	Bubbling FB	Air	-	Semi-Industrial (0.8)	Dolomite (Experimentally)	700–900	GS	Fluent	[332]
MSW	Bubbling FB	Air-Carbon Dioxide	-	Semi-Industrial (0.8)	Dolomite (Experimentally)	500–900	GS	Fluent	[279]
MSW	Bubbling FB	Air-Steam-Carbon Dioxide	-	Semi-Industrial (0.8)	Dolomite, NiO/MD Catalyst	700–900	GS	Fluent	[333]
MSW	Bubbling FB	Air	-	Semi-Industrial (0.8)	-	500–700	GS	Fluent	[280]
MSW	Bubbling FB	Air	-	Semi-Industrial (0.8)	Dolomite (Experimentally)	~ 500–700	GS	Fluent	[224]
MSW	Plasma/Melting	Air-Steam	-	Pilot (2.7)	-	~2200 max	GS	Fluent	[225]
MSW	Plasma/Melting	Air	-	-	-	-	GS	-	[65]
Biomass & Plastic (Wood, PE) ^1^	Rotary Kiln	-	-	-	-	-	GS	Fluent	[310]
Biomass	Circulating FB	Air	-	Pilot (0.2)	-	~400–1000	GS	Fluent	[334]
Biomass (Bagasse, Rice husk, Switchgrass)	Bubbling FB	Nitrogen	-	Lab (0.006)	Sand	400–600	GS	Fluent	[335]
Biomass (Coffee husk)	Bubbling FB	Air	-	Pilot	-	~600–1400	GS	Inhouse (COMMENT)	[336]
Biomass (Forest residues)	Bubbling FB	Air	-	Pilot (1)	Dolomite	~ 800	GS	Fluent	[337]
Biomass (Forest residues, Peach Pits, Ground Coffee)	Plasma	Air-Steam	-	-	-	1000–2000	GS	Inhouse (COMMENT)	[338]
Biomass (Pinewood)	Vortex Reactor	Nitrogen	<1 (order of ms)	Lab (0.0001)	-	500–600	GS	Fluent	[339]
Biomass (Wood)	Bubbling FB	Air	-	Lab (0.0004)	Sand	~ 900	GS	Inhouse (FORTRAN)	[340]
Biomass (Wood)	Bubbling FB	Air	-	Lab-Pilot (0.01)	-	700–750	GS	ModifiedK-FIX	[341]
Biomass (Wood)	Bubbling FB	Air	-	Lab (0.0004)	Sand	850	GS	MFIX-based	[342]
Biomass (Wood)	Bubbling FB	Air	-	Lab-Pilot (0.01)	-	~400–800	GS	-	[343]
Biomass (Wood)	Fixed Bed	Air-Steam	-	Pilot (0.22)	-	~450–1000	GS	Fluent	[344]
Biomass (Wood)	Fixed Bed	Air-Steam	<1 (order of ms)	-	-	~650–1300	GS	-	[345]
Coal	Bubbling FB	Air-Steam	-	Pilot (0.07)	-	~400	GS	OpenFOAM	[306]
Coal	Bubbling FB	Air-Oxygen-Steam	-	Pilot (1)	Silica Sand	~900	GS	Fluent	[346]
Coal	Bubbling FB	Air	-	Lab (0.1)	-	~600–1000	GS	Fluent	[347]
Coal	Bubbling FB	Air-Steam	-	Lab (0.07)	Limestone	812, 855	GS	ANSYS	[270]
Coal	Bubbling FB	Air-Steam	-	Lab (0.07)	Sand	821, 846, 855	GS	-	[348]
Coal	Bubbling FB	Air-Steam	-	Lab (0.07)	-	812-866	GS	-	[349]
Coal	Entrained Flow	Air	-	Commercial (15)	-	~370–2000	GS	CFC code PHOENICS (Inhouse)	[350]
Coal	Fixed Bed	Air-Steam	-	Lab (0.01)	-	~600–1300	GS	MFIX	[351]
Glycerin solutions containing xanthan gum ^2^	Bubble Column	Air	-	Lab (0.01)	-	25	GL	CFX (MUSIG)	[352]
Glycerol	FB	Steam	-	0.001	Sand	600–750	GS	Fluent	[353]
Manure Slurry ^2^	Anaerobic Digester	-	-	Industrial (791)	-	35	GL	Fluent	[354]
Water	Bubble Column	Air	-	-	-	-	GL	OpenFOAM	[355]
Water	Bubble Column	Air	-	Lab (0.01)	-	Room	GL	OpenFOAM	[286]
Water ^3^	Bubble Column	Air	-	Lab (0.007) Pilot (4)	-	-	GL	OpenFOAM (OpenQBMM)	[356]
Water	Laboratory Tank	Air	-	Lab (0.02)	-	22	GL	OpenFOAM	[274]
Water ^3^	Vertical Tube	Air	-	Lab (0.01)	-	-	GL	OpenFOAM (twoWayGPBEFoam)	[357]
Water ^4^	Bubble Column	Air	-	Lab (0.07)	-	-	GL	-	[358]
Water ^4^	Bubble Column	-	-	-	-	-	GL	-	[359]
Water ^4^	Bubble Column	Air	-	Lab (0.007) Pilot (0.27)	-	30	GL	CFX	[360]

^1^ VOF method was used. ^2^ Non-Newtonian fluid. ^3^ Quadrature-Based Method of Moments (QBMM) method was used. ^4^ Population balance model (PBM) was implemented.

Simulation of similar processes to PWG can be also of great help. Zhong et al. [226] have simulated the gasification process of pitch-water slurry in two different types of gasifiers using the E-L method. A down-draft single nozzle (pilot-scale) and an opposed multi-nozzle (industrial-scale) entrained-flow gasifier were simulated in this study. Pitch, which is produced from the deasphalting unit, is a heavy compound with a high softening point [226]. Hence, it can be very similar to plastics from the gasification behavior point of view. To validate their framework, they first simulated the gasification of Orimulsion^TM^ (an emulsion of 70% bitumen and 30% water) in the presence of oxygen. Then, they assessed two different gasifiers for the oxygen-gasification of pitch-water slurry. In this study, the pitch particles are surrounded by a water layer to form the pitch-water slurry to be pumped at the top of the gasifier. The simulation framework in this study includes models for slurry atomization, water evaporation, pitch pyrolysis, and heterogeneous and homogeneous gasification reactions. A global reaction scheme of 12 reactions is considered for pyrolysis and gasification reactions. Although water evaporation and pitch pyrolysis is considered in the mass evolution of the particle, the effect of water on the hydrodynamic behavior of the particles has not been reported. They also studied the particle residence time and their conversion, which can be an important parameter also in the PWG.

From Table 6 and Table 7, it can be observed that the average size of the units assessed by different E-L approaches is smaller than the ones modeled by the E-E approach. This demonstrates the limits of the E-L method due to the computational costs. However, with the increase in computational power and the more precise results of this approach, it is expected that, in the near future, the PWG units will be designed and optimized using this approach.

### 7.2. Multi-Phase Flow Modeling Challenges and Possible Solutions

The simplifying assumptions (mentioned at the beginning of this section) can negatively affect the simulation results and their reliability to be used as design and optimization tools. This is while there are some possible solutions to remove those assumptions and increase the precision of the simulations. In the following subsections, these challenges and possible methods to overcome them in different modeling approaches are discussed.

#### 7.2.1. Irregular Shape

If the gasification plant is used to gasify the real-world solid plastic, the feedstock particles are normally in different shapes and roughness. The variety in particle shape of the PW feedstock is a challenge to be dealt with in the reactor-scale simulations in E-E and E-L frameworks. According to Athanassiadis et al. [361], the particle shape has a strong impact on their stress-strain relationship and they declared that this is attributed to their different contact types, as the result of different shapes [362].

In the E-E framework, a detailed description of the particle shapes is not possible since they are defined as a fluid phase. However, an important parameter in this regard is the drag force, which was discussed in Section 6.4.1 and the non-spherical drag correlations can be found in the review article of Ullah et al. [244]. The more challenging part of this problem is that the shape of the particles and their non-sphericity changes due to the attachment of the particles, or their consumption in the pyrolysis and gasification reactions. To the best of the authors’ knowledge, this has not been studied yet for PWG, while it can have drastic effects on the results.

**Table 7:** Overview of Eulerian-Lagrangian simulation studies for different feedstocks and reactor designs that can be used as a guide in the PWG simulations.

Feed	Type	Gasification Agent/Process Gas	SRT (s)	Plant Size (OM-m^3^)	Bed Material	Temperature (K)	Lagrangian Approach	Software	Ref
Plastic (PE, PP, PS, mix)	Entrained Flow	Air	-	Lab (0.005)	-	~50–1100	-	Fluent	[29]
Pitch-water slurry	Entrained Flow	Oxygen	0–50	Pilot (0.2)/Industrial (33)	-	~1500	-	Fluent	[226]
-	Conical Spouted Bed	Air	-	Lab (0.001)	ZiO_2_	25	DEM	Fluent	[363]
Biomass	Bubbling FB	Air-Steam	-	Lab (0.003)	Sand	~800–900	DEM	-	[323]
Biomass	Spouted Bed + DFB	Steam	-	SB Lab (0.01)/DFB Pilot (0.3)	Silica Sand	820–870	MP-PIC	OpenFOAM	[364]
Biomass (Almond prunings)	DFB	Steam	Up to ~100	Pilot (0.7)	Sand	~400–900	MP-PIC	OpenFOAM	[365]
Biomass (Glucose)	FB	Super Critical Water	-	Lab (0.001)	Quartz Sand	~500–600	DEM	Fluent	[366]
Biomass (Pinewood)	Bubbling FB	Steam-Nitrogen	-	Lab (0.06)	Sand	820–920	CGM & DEM	STAR-CGM+12.02	[367]
Biomass (Pinewood)	Bubbling FB	Steam-Nitrogen	-	Lab (0.0005)	Sand	820–920	DEM	OpenFOAM	[368]
Biomass (Pine, Beech, Holm oak, Eucalyptus)	Conical Spouted Bed	Steam-Argon	-	Lab (0.01)	Sand	770–920	MP-PIC	OpenFOAM	[369]
Biomass (Pine, Beech, Holm oak, Eucalyptus)	Entrained Flow	Air-Steam	Up to ~2.5	Lab (0.01)	-	1000–1400	-	OpenFOAM	[370]
Biomass (Raw, Torrefied)	FB	Air-Nitrogen-Steam	-	Lab (0.0001)	Olivine	750–850	DEM	OpenFOAM	[371]
Biomass (Raw, Torrefied) (Forest residues, Spruce)	Entrained Flow	Air-Steam	-	Lab (0.01)	-	1400	-	OpenFOAM	[372]
Biomass (Rice husk)	Entrained Flow	Oxygen-Steam-Carbon Dioxide	-	Lab (0.01)	-	1400	-	OpenFOAM	[373]
Biomass (Rice husk, Cotton stalks, Sugarcane bagasse, Sawdust)	Concentric tube entrained flow	Oxygen	-	Pilot (0.25)	-	~900–2300	DPM	Fluent	[374]
Biomass (Sawdust)	Entrained Flow	Air		Lab (0.015)	-	800–1000	DPM	Fluent	[375]
Biomass (Sawdust, Cotton trash)	Entrained Flow	Air-Steam	-	Pilot (4)	-	~800–1100	-	CFX	[376]
Biomass (Wood pellet)	FB	Steam	Up to ~36	Lab (0.02)	Sand	~600–800	CGM-DEM	Fluent	[377]
Biomass (Wood)	Bubbling FB	Steam	-	Lab (0.06)	Sand	820	DEM	Inhouse (MFIX-DEM)	[378]
Biomass (Wood)	FB	Air	Up to ~84	Lab (0.01)	Charcoal	~500–700	DEM	-	[379]
Coal	Bubbling FB	Air-Steam	Up to ~20	Lab (0.07)	Sand	~ 800	MP-PIC	OpenFOAM	[380]
Coal	Circulating FB	Air	-	Pilot (0.2)	Sand	~600–850	MP-PIC	-	[381]
Coal	Circulating FB	Carbon Dioxide-Oxygen-Nitrogen	-	Pilot (0.03)	Sand	~950 (max)	DPM + MP-PIC	Fluent + CPFD Barracuda	[382]
Coal	Entrained Flow	Oxygen-Steam	-	Industrial	-	1370–1620	-	Fluent	[383]
Coal	Entrained Flow	Air-Steam	-	Lab (0.004)	-	~200–1850	-	Fluent	[384]
Coal	Entrained Flow	Air	-	Pilot (0.26)	-	~700–1900	-	CFX + FORTRAN	[385]
Coal	Two-stage Entrained Flow	Oxygen	-	Industrial (32)	-	~700–2100	DPM	Fluent	[386]
Coal	Updraft gasifier	Air-Steam	-	Industrial (60)	-	~500 (mean)	DPM	Fluent	[387]
Water ^1^	Bubble Column	Air	-	Lab (0.01)	-	-	DEM	OpenFOAM	[388]
Water ^1^	Bubble Column	Air	-	Lab (0.01)	-	-	DEM	OpenFOAM	[389]

^1^ Gas-Liquid system.

In the unresolved E-L framework, the same approach can be followed to include the effect of particle shapes in the phase interaction and drag force. In the resolved simulations, the phase interactions are calculated directly on the particle surface and don’t need the closure models. Moreover, if every particle is tracked in the E-L framework, e.g., the DEM method, extra precision can be added to the simulations regarding the particle shape. There are two main approaches in the DEM simulations to model irregular particle shapes [390]. The first one is the single-particle approach in which fixed shapes, such as circular, ellipse, polygon, superquadrics, or arbitrary shapes are considered to approximate the shape of the desired particles. The drawback of this approach is the limit in the number of pre-defined shapes and less accuracy because of the approximations that are used in this method. The second approach is called composite, clustering [390], or multi-sphere [362] method. In this method, the non-spherical shapes are reconstructed by a set of attached regular shapes, which form a cluster of particles. Consequently, each irregular particle counts as the number of its sub-particles. Besides, the contact of the particles in the DEM framework is calculated based on the contact between their sub-spheres [362], and this results in higher computational costs, which is the drawback of this method [390]. It has been demonstrated that since the interactions of each of the sub-particles have to be taken into account, this can cause a non-linear increase in the computational cost [391].

The irregular shapes can be also added to simulations in case of having liquid in the system. If the bubbly flow is considered for the PWG, the shape deformation of bubbles can be also captured in an E-L framework. In the high-viscous non-Newtonian molten plastic, this deformation becomes more important since the stabilization time for deformed bubbles is higher compared to the normal liquids. The solver (set of models) developed by Peña-Monferrer et al. [389] in the OpenFOAM environment can be a useful tool/guide in this regard. In this solver, it is possible to track the bubble path and expansion—although the coalescence and break-up of the bubbles are ignored to isolate the bubble dynamics and its interaction with the main fluid. Moreover, the bubble’s deformation is considered only in the case that they collide with each other or the wall. In this approach, the volume of the bubbles is considered to be constant and their surface is deformed.

#### 7.2.2. Roughness

Besides the particle shape, their roughness can also play an important role in their hydrodynamic behavior and interactions with other particles, as well as the other phases. This can not be accounted for in the normal E-E simulations. The reason lies in the fact that most of the KTGF models don’t take into account particle roughness [392] and rotation, though this has been shown to have important effects on the stresses in a quasi-static regime [393]. Modified KTGF models can partially solve this problem.

Yang et al. have developed a modified KTFG model to take into account the particle roughness and their rotation [392]. A first-order velocity distribution function was obtained for the particles, which takes into account particle rotation and friction. This has been done by considering the tangential restitution coefficients, as well as the friction coefficient. Similarly, they developed expressions for the flux of translational and rotational fluctuation energy. This was used to account for both the particle slip at the wall and for sticking particle–wall collisions. This resulted in defining a new boundary condition for the particle slip velocity [394]. The modified model was compared to the original KTGF, as well as similar friction-included KTGF models, and demonstrated acceptable results. Moreover, the performance of the model together with the developed boundary conditions was validated against the results of an E-L CFD simulation approach [395] and the experimental data of Magnetic Particle Tracking [396].

The particle roughness is in fact due to its irregular surface shape but on a very small scale. So, in principle in the E-L approach, it can be considered by modeling the particle’s irregular surface shape. However, it requires a very fine resolution of mesh so it can capture the surface’s irregular shapes [397]. Hence, in the DNS, this may be possible but increases substantially the computational cost, which is not desirable. Consequently, the friction factor can be increased in their contact model to capture the particle surface roughness [398].

#### 7.2.3. Polydispersity

The polydispersity of particles can be due to the feedstock characteristic or the consumption of the particles and changing their size. The agglomeration and detachment of the particles can be also another reason for variations in the particle size [399], which is discussed in the next sub-section. The same is observed for droplet and bubbly flow regimes. This can have drastic effects on the simulation results since it directly affects the interfacial area for the heat, mass, and momentum transfer.

As was also declared by Sia et al. [335] the conventional single-dispersed-phase E-E framework is not capable of handling well the polydispersed systems such as PWG. However, there are possibilities to take into account the polydispersity (due to the feedstock characteristic) using the more recent E-E approach. The first one is to use multiple solid phases of different sizes. This can not be used for the continuous change in the particle properties [399]. Hence, this is limited to a few classes of the particles, with different sizes (and/or density, roughness, etc.). Mathiesen et al. [400] implemented the extended KTGF model to use multiple solid phases in their simulations. They took into account three different solid phases with different diameters in the cold-flow simulations of a circulating FB reactor. Similarly, it is possible to define different droplet or bubble phases with different sizes, if their interaction is assumed to have a negligible effect on their size distribution [389]. Following this approach, Cubero et al. have extensively studied the effect of polydispersion on the flow regime, reaction dispersion, and local concentration of different species in a gasification process [306]. They considered seven solid phases from 147 to 1456 μm. An important aspect in this regard is that the extension of the KTGF model to multiple solid phases is not enough and the binary effect of solid phases on each other should be taken into account. To do so, Cubero et al. have accounted for the particle-particle drag force [306] using the correlation proposed by Syamlal [401]. This correlation expresses the drag force between different classes of solids as a function of their density, diameter, binary radial distribution, binary restitution coefficient, and slip velocity [401]. They also took into account the binary collisional pressure in the KTGF model as well as the interphase heat transfer between each solid class and the fluid. However, the heat transfer between different classes of solids, as well as their thermal interaction due to the collision was neglected. This is reported to be a common practice in E-E simulations [306], which can be justified by the intense mixing in their reactor and almost uniform temperature in the bed (maximum 6% temperature difference between different classes). 

The second approach to deal with polydispersity, which is more advanced, practical, and precise, is by means of population balance equation (PBE) that can be used to represent the continuous variation of the dispersed phase properties and is computationally faster [399]. Moreover, it can be implemented to simulate the change in size due to the reactions. In this method, the balance equations are solved for the number density function (distribution of particle sizes), which is affected by the rate of change for each particle size. More details on this method can be found in [402].

There is not a single approach to solve the population balance equations. MOM is one of the frequently used methods in the literature to solve the PBE [399] and it is also not done in a single way. One of the common approaches of the MOM method is the quadrature-based moments [403,404], which considers a polynomial for the distribution of the particle sizes. There is not any unique expression to define the size distribution function and consequently, different approaches have been proposed in the literature such as the extended quadratic method of moments (EQMOM) [405], finite-size domain complete set of trial functions method of moments (FCMOM) [406], and direct quadrature method of moments (DQMOM) [407], among others.

As mentioned before, PBE can be implemented in a CFD framework to account for the change in the particle size due to the consumption, which is very helpful in PWG simulation, whether it is assumed a gas-solid-only or molten plastic droplets system. Using the FCMOM, Ghadirian et al. have coupled a PBE with an E-E framework to simulate a gasification process [399], including the size and density distribution of the particles. To be implemented in the CFD simulations, the PBE should be coupled to the continuity, momentum, and energy balance equations [408], as well as the chemistry, to (1) get data from the flow, (2) update the size distribution, and (3) send the data back to the CFD equations [399]. As an example, they added a source term in their PBE equations for the reaction rate that changes the particle size. So, the PBE gets the chemistry from CFD, solves the population balance equations, and gives back the updated particle size, which affects the hydrodynamic in the system. If the reaction kinetic is also expressed as a function of particle size or surface, then the loop between PBE and CFD becomes closed. They demonstrated the important role of particle size on the hydrodynamic and reaction rate of the system. This is crucial for the systems in which the size of feedstock decreases due to consumption, such as PWG. According to Ghadirian et al. [399], the mixing effects became highlighted in the middle of the reaction—due to the bubble formations—where the particles’ size decreases. However, moving toward the end of the reaction and decreasing the size of the particles, due to the less bubble formation, the mixing effects decreased as well. The average size of particles in their simulation decreased from 450 μm to 150 μm in 30 s of the gasification reaction.

In the E-L approach, particle size change due to the reaction can be captured directly, which is an opportunity that is available in this approach, if they can be tracked. The size change can be correlated to the change in mass, which is present in the continuity equation [409,410].

#### 7.2.4. Aggregation, Coalescence, and Breakup

In the gasification of PW, change in the particle/droplet size is not only due to the consumption but due to aggregation and breakage. This is supposed to be more highlighted in the situations where the presence of molten plastic increases the chance of agglomeration due to its stickiness. This challenging situation can be also solved by implementing the PBE.

Fan et al. have implemented a DQMOM to account for the aggregation and breakage in a FB reactor by considering a source term in the continuity equation of the solid phases [408]. To do this, different kernels can be defined. These kernels are in fact the patterns and functions that determine the consequence of collisions on the size evolution of the dispersed phase. Fan et al. considered different kernels for aggregation and breakage, which are proportional to the collision and disruption of the particles, respectively. For the breakage, a function is also defined to describe the fragmentation due to the breakage. These kernels can be considered as constants (which means they are assumed to be independent of the particle or flow properties) or alternatively, can be derived from the KTGF [408]. They applied their developed model in the CFD simulation of a fluidized bed HDPE production and validated their model for the segregation in polydispersed FBs [407]. Moreover, the defluidization phenomena were captured in their model. Hence, for simulating PWG, which can face this problem [19,281,411], this can be a valuable tool by tuning the kernel parameters based on the sticking properties of molten plastic. It should be noticed that sticking intensity or the minimum stress that causes breakage can change with conditions, e.g., the temperature. However, this is not probable to be captured even by using the KTGF-based kernels, while it can be an important aspect in the PWG.

The coalescence and break-up of bubbles and change in their size in bubbly flow can be similarly captured using population balance methods [358,360]. In this method, the coalescence and breakup of the bubbles can be modeled based on the collision frequency and the coalescence tendency [358]. Similar to the solids, this method solves the transport equation of weighted abscissas and birth and death rate of the bubbles. Heylmun et al. [356] have developed a solver in OpenFOAM (under the project called OpenQBMM) to simulate the polydispersed bubbly flows based on the quadrature-based moment method, taking into account the evolving size distribution and coalescence and breakup. This solver showed good agreement with other simulation works and estimated well the pressure fluctuations as was reported in the experimental data. However, the solver is incapable of capturing well the flow behavior (e.g., gas hold-up) in cases with a high gas flow rate, which can be observed also in the standard two-fluid models. This can be a weak point for simulating the gasification process in which the gas flow rate is usually high.

If these phenomena are going to be captured in the E-L framework in which the particles or bubbles are tracked, different approaches are used. Before expanding this topic, an important drawback of the unresolved E-L simulations—which can get highlighted in cases such as PWG—should be discussed. As was mentioned before, the common practice is to use cell sizes that are at least three to five times larger than the particle size [322]. This limit can be an important constraint in the intensified processes, in which high velocity and turbulent flows are indispensable. The weakness comes from the fact that to better capture the flow properties at high fluid velocities, the cell size should be small enough to maintain the low courant number in the simulations ($Cou=\frac{u\Delta t}{\Delta x}\leq Cou_{max}$). On the other hand, and especially for the PW or similar feedstock, large particles may be present in the feedstock. Even if the particle size is not large at the beginning and the cell size criterion is met, the agglomeration of the particles/droplets/bubbles can increase their size. Hence, coarse grids (to be able to fit the discrete phase in a cell) should be used, which can cause instability problems or inaccuracy in the discretized solutions because of the high velocity of the fluid. A possible solution to this problem is using the multi-grid solver. In the simplest case of this method, one fine and one coarse grid are defined for the fluid. The fine one, on the one hand, is used for simulating the fluid phase flow to prevent the instabilities and inaccuracy that can happen using the coarse grid. The coarse grid, on the other hand, can be used for two goals: first, to obtain the initial reasonable solution (guess) of the fluid flow, based on which the solution on the fine grid can be achieved faster and at a lower computational cost; and second, to solve the particle flow. To couple the fine grid solution of the fluid flow and the coarse grid solution of the particles, the obtained fluid solution is mapped from the fine grid to the coarse grid by arithmetic averaging [412]. 

Even if the presence of liquid in the system is not dominant, it can cause sticking the solid particles to each other (aggregation). To the best of the authors’ knowledge, no article has been published on the PWG in an E-L framework, which includes the effect of liquid presence, and hence, other studies with similar phenomena can be helpful in this regard. Breuninger et al. [363] studied the spouting behavior of a spouted bed reactor of 1.7 million ZrO_2_ particles, which are cohesive powders. In their DEM framework, they considered adhesive forces in addition to drag, gravitational, and contact (normal and tangential) forces.

For the particle-wall interactions, a similar method can be followed to study the sticking of the particles on the surfaces and their agglomeration (and ultimately fouling—Section 2.2). The fouling has been shown to start with the attachment of single particles to the wall, which can be a random phenomenon. The main contributors to this phenomenon are the reactor wall morphology [413] and the flow behavior. The principles of this simulation are similar to the one that was explained above. As a starting point of the framework (initial conditions), it is assumed that a few particles have been already attached to the wall. The incoming particles to the system that collides with those particles, can attach to them based on their contact forces. This approach was followed by Trofa et al. [413] to simulate the particulate fouling in microchannels, which can be a great help for the cases in which the interaction of particles with the wall is important. 

To decrease the complexity and computational costs of simulating such cohesive forces, one approach is to relate this force to the weight of the particle, i.e., they can be assumed to be a function of the gravitational force exerted on the particle [414]. Hence, an extra force, which is a multiplication of weight by a “cohesive force factor” is added to the Newtonian equations of the particle. On the contrary, to increase the precision of simulating the presence of sticky molten plastic in the PWG simulation, the studies of wet particles’ behavior in the fluidized bed reactors are of significant help. In the numerical study that is done by Song et al. [324], a four-stage contact model between the wet particles is considered, which is based on a hysteresis linear spring-dashpot contact model. In this approach, the restitution coefficients of both dry and wet particles play important roles. Hence, the liquid phase properties (viscosity and surface tension) become important since they are used to calculate the spring stiffness, the restitution coefficient of wet particles, and the liquid bridge forces.

Besides the wetted particle approach that was described above, similar methods can be implemented to simulate the coalescence and break up of bubbles or droplets in the cases that liquid molten plastic presence is dominant in the system. For the droplets, simulating the coalescence and breakup is similar to what was explained for the wet particles. However, it is associated with more complexity, because the shape deformation is also added to the other phenomena. For the bubbles, the E-L approaches implemented in bubbly flows can provide guidelines for the PWG simulations. The principles of this approach are similar to the gas-solid framework that was explained above, but different models can be used for the coalescence and breakup phenomena. Xue et al. [388] have simulated the bubbly flow in an E-L framework using the spring-dashpot model to evaluate the collision and contact time of the bubbles. Subsequently, they have implemented the film drainage [415] and bubble breakage models to reflect the coalescence and breakup of the bubbles, respectively. For the former, it is assumed that the coalescence happens in three stages: (1) bubble collision, (2) draining the liquid, and (3) film rupture and coalescence [415]. Consequently, when two bubbles get in contact with each other, if the liquid film drainage time is shorter than the bubbles’ contact time, the coalescence happens [415]. Hence, important parameters are the contact and drainage time. The contact time is a function of coalescence frequency, which is determined by the flow behavior. The drainage time, however, is a function of bubble diameter, liquid properties, and initial and final film thickness. The last two parameters are determined based on the gas-liquid pair. Consequently, for the PWG, this can get very complicated for the same reasons that were described in determining the thermophysical properties in Section 3. Moreover, the coalescence happens due to different mechanisms, i.e., turbulence, buoyancy, and laminar shear, which are described in detail in [415]. The modeling of these mechanisms should be done separately based on their occurrence in different locations of the PW gasifier. Similarly, the breakup mechanisms can be different, which are turbulent fluctuation, shear stress, and interfacial instability [388]. However, the complexity here, compared to the coalescence, is determining the sizes of bubbles after breakage (daughter bubbles). This can be done by defining the probability density functions in the size of the daughter bubbles [388].

#### 7.2.5. Regime Transition

A situation that is common in the bubbly flow regimes, and can happen in other flows as well, is the regime transition [355] in which the continuous phase becomes the dispersed phase in some zones of the reactor. This is likely to happen in PWG because of the aggregation, coalescence, and breakup that were discussed earlier. In these kinds of cases, special care has to be taken in implementing the closure models since they are usually derived for a constant continuous/dispersed phase binary. For example, the drag coefficient correlations are usually reported for, e.g., a single bubble in a bulk of liquid while in some reactor zones, this could be changed into a single liquid droplet in a continuous gas phase. Other than the closure models, another related problem is that the volume fraction that is occupied by each phase can change the dominant dispersed phase and this can cause singular problems [286]. This is more problematic in the E-E simulations in which the phases are considered continuum. In this situation, a numerical method to switch the definition of the dispersed phase is required, which is called the blending function. Li and Christian used a blending function to simulate a bubbly flow [286]. The logic of the blending function is to define a criterion based on the volume fraction of phases in cells to determine the dispersed phase. If the volume fraction of a phase becomes higher than the limit, it becomes known as the continuous phase and the heat, mass, and momentum exchange is calculated based on the other phase, which has changed into the dispersed one. More details on this approach are found in [286]. 

#### 7.2.6. Non-Newtonian Behavior

The last important parameter in the simulation of PWG for the systems in which the effect of the presence of molten plastic on the hydrodynamic is highlighted is the high viscosity and non-Newtonian behavior of molten plastics [187,188]. Although this is less important at high temperatures of the gasifier, to simulate the transient situation in which the molten plastic temperature is still low, it can get crucial. Yin et al. [311] assessed a novel falling film reactor design to pyrolyze the PW. Although they declared the non-Newtonian behavior of the molten plastics, they based their simulations on the Newtonian fluid flow [169,311]. Unfortunately, to the best of the authors’ knowledge, this has not been yet considered in the PWG simulation or similar processes. However, it can be inspired by similar research studies from a hydrodynamic point of view [352,354,416]. To include the non-Newtonian behavior of the molten plastic, the most important parameter is to define the viscosity dependence on the shear rate [352]. Also, Reynolds number and pressure drop can be modified accordingly [354]. Although its implementation is not complex, the computational cost and instability will increase due to the addition of this degree of freedom. 

### 7.3. Multi-Scale Frameworks and Computational Efficiency

**Figure 16:**
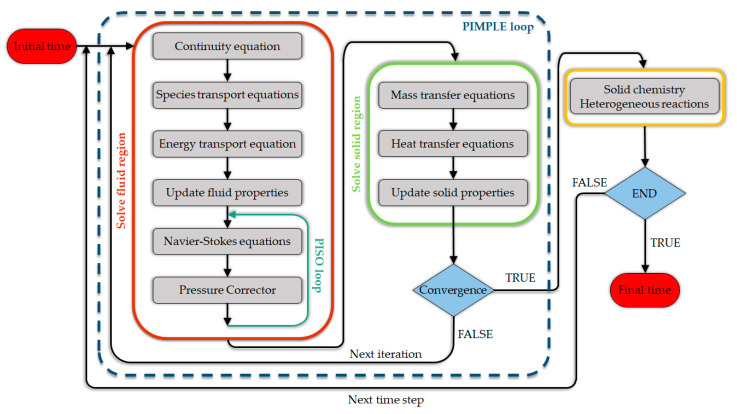
Multi-region catalyticFoam solver algorithm (Redrawn from Ref. [418]). Reprinted from Chemical Engineering Journal, Vol. 283, Tiziano Maffei, Giancarlo Gentile, Stefano Rebughini, Mauro Bracconi, Filippo Manelli, Stefan Lipp, Alberto Cuoci, Matteo Maestri, A multi-region operator-splitting CFD approach for coupling microkinetic modeling with internal porous transport in heterogeneous catalytic reactors, Pages No. 1392–1404, Copyright (2015), with permission from Elsevier. Multi-region catalyticFoam solver algorithm (Redrawn from Ref. [418]). Reprinted from Chemical Engineering Journal, Vol. 283, Tiziano Maffei, Giancarlo Gentile, Stefano Rebughini, Mauro Bracconi, Filippo Manelli, Stefan Lipp, Alberto Cuoci, Matteo Maestri, A multi-region operator-splitting CFD approach for coupling microkinetic modeling with internal porous transport in heterogeneous catalytic reactors, Pages No. 1392–1404, Copyright (2015), with permission from Elsevier.

Although the state-of-the-art multi-scale platforms try to integrate the phenomena at different scales, due to computational and technological limits, they are still far from the ideal situation, which includes all the models from atomic to reactor scale in one modeling platform. This is especially challenging and cumbersome for the PWG. However, for simpler cases, efforts have been made to increase the number of different scales’ phenomena in a single platform. In most of the CFD simulations of multi-phase flows on the reactor scale, the intraparticle phenomena are usually neglected. Recently, however, some efforts have been made to create multi-scale platforms, from intra-particle phenomena to the reactor scale flow. In this regard, Oschmann et al. [417] extended a DEM framework to account for the 3D intra-particle heat transfer mechanisms of the spherical and non-spherical particles, which can increase the precision in simulating the interaction between particles and also between the particles and the wall or the fluid phase. In a more advanced approach, Hardy et al. developed a PR-DNS solver for the weakly compressible and reactive flows [230]. This solver is capable of including internal and interfacial heat and mass transfer together with the reactions. For the PWG, this can be a promising framework due to its capability for the complex reactive flow systems in which the density of the gas can change in different locations of the reactor. Another example of such a comprehensive multi-scale platform is a solver called catalyticFoam that has been developed at POLIMI [418,419]. This simulation platform is able to include the detailed chemistry of heterogeneous catalytic reactions and solve the transport equations for both the fluid and solid phases by implementing a multi-region operator-splitting approach. This results in calculating the concentration and temperature gradients in the solid particles as well as in the fluid phase. This means that the simulation mesh is resolved in the particle regions and the transport equation for intra-particle phenomena is solved in those regions, in addition to the fluid phase. Figure 16 illustrates the algorithm of the mentioned solver. To couple the fluid and solid phases, they implemented a partitioned approach, in which the equations at the interface of both regions are solved and then iteratively adjusted to converge the solution variables at their interface. This solver is designed for a fixed bed catalytic reactor and the flow is considered laminar. Hence, it is still far from what is needed to simulate the real FB PWG. However, it can be a useful initiative based on which a precise multi-scale PWG modeling platform can be built. It should be noticed that including the Newtonian equations for the discrete phase movement would increase a lot the complexity and computational costs of this framework. But considering the advances in computational resources, this can be done soon. 

As can be inferred from the discussions made above, creating a single multi-scale modeling platform to include all the phenomena from sub-atomic to reactor scale is not possible with the current technological and computational resources. However, this can be obtained by coupling different frameworks, which have been developed by different research groups. In this regard, the open-source software packages—which are widely and freely available—are a promising option, especially because they are flexible to be modified for a special application and coupling different platforms. Besides, another important aspect that should be investigated is computational efficiency. For modeling these kinds of complicated multi-phase flows, a huge amount of computational resources are needed. Hence, smart utilization of those resources is a step forward in this field. This is done by dividing the problem domain into multiple sections and using parallelization techniques to use multiple computing cores on high-performance computing systems (HPCs). Depending on the complexity and the desired simulation method, different parallelization approaches are used. This was initially performed on CPUs (Central Processing Units). In this method, usually from a few cores up to the order of magnitude of hundreds of CPU cores are employed to solve the fine mesh problems. However, in the recent decade, developing tools and packages, such as CUDA (NVIDIA Corporation, Santa Clara, CA, USA) [420], has enabled many-cores parallelization, e.g., using GPUs. This way, it is possible to use a much larger number of cores—e.g., one order of magnitude—compared to CPU parallelization. This type of parallelization is usually used for cases in which a larger number of, but less complicated calculations, have to be solved compared to the cases which use CPU parallelization. Using GPUs, it can be possible to simulate the PWG, and in general all multi-phase applications, with a very large number of discrete phase elements (hundreds of millions), for engineering scale applications [421]. Also, another opportunity is to use hybrid approaches. Norouzi et al. [422] have developed a solver to implement CPU parallelization for solving the governing equations of the fluid phase (CFD) and GPU parallelization for DEM calculations via the CUDA platform. They used their framework to efficiently simulate a bubbling FB with a large number of particles using a normal computer in a reasonable amount of time. Hence, the complex and computational expensive simulations of multi-phase PWG systems can be hopefully done at high resolution and with precise models at logical costs via these recent efficient computational methods. 

## 8. Conclusions

PWG is a growing research area because of the drive to move to a more circular economy and its inherent advantages over landfilling, mechanical recycling, or mere energy recovery. The most important strengths of PWG are:

- It does not require extensive sorting of PW.
- It does not necessarily require a catalyst that could be easily deactivated by impurities present in PW.

The most important challenges are:

- The wide variety of plastic types and elements in PW that result in the necessity of substantial upgrading of the syngas, e.g., removal of HCl, or dealing with the fluctuations in the feedstock composition.
- PWG setups are studied and designed based on the existing knowledge when gasifying coal or biomass, and hence are believed to be not optimal for PW. In particular, the presence of liquid is usually neglected.

In this regard, both experimental and numerical methods are essential to improve our fundamental understanding. However, the analysis in this work showed that there are many aspects in the multi-scale modeling of PWG that have not been considered, or if so, not modeled precisely. With the current increase in computational power it becomes possible to develop more fundamental and precise multi-scale models in the coming decade. Accurate modeling approaches of the chemistry and transport phenomena at various scales and a combination of different scales through proper scale-bridging methods will allow a more robust and reliable in-silico optimization of the process, which may eventually lead to a more economic industrial application. Hence, in the following paragraphs, the most important conclusions of this work are explained in detail.

Many of the current experimental data for the PWG are limited to a product distribution, which can be useful only for validating the performance of the whole multi-scale framework, and not the individual sub-models at each scale, e.g., heat or mass transfer models. Hence, besides the necessity of developing specific models for this process, accurate and detailed experimental data, specifically for PWG, should be gathered to validate different models at different scales of the multi-scale framework.

The current feedstock data and models are not appropriate enough to increase the accuracy of the micro-scale models. Liquid and gas-phase pyrolysis reactions have to be accounted for and the coupling to the gasification reactions should be done. Currently, reliable kinetic models to be used in the multi-scale framework are almost not existing, and if they exist, they are limited to a small number of polymer types and pyrolysis. With a more accurate characterization of feed—both the waste stream composition and the detailed composition of its individual components—it is possible to create more reliable kinetic models for the pyrolysis and gasification reactions.

On the particle scale, various phenomena can occur that depend on the reactor type. Even in the simplest case, the multi-component/non-Newtonian molten plastic is present, which complicates modeling the transport phenomena on this scale. This is while the reactions in the liquid and gas phases, as well as the phase transformation, occur concurrently. This is not usually assessed in the multi-scale modeling frameworks of PWG. Hence, it can be concluded that the understanding and modeling of the phenomena that are highlighted in the particle scale should be improved and coupling of both the molecular and particle scale phenomena should be investigated more fundamentally.

Apart from the phenomena at the molecular and particle scales, the interaction among discrete phase fragments (particles, droplets, bubbles) and with the reactor walls can be captured in the reactor models. This includes practical 0D/1D and 3D CFD models to incorporate the effect of multi-phase flow in the multi-scale framework. Hence, considering the improved computational power, the engineering models can be used to create a multi-scale model including the detailed kinetic models, the E-E approach can be applied in engineering studies, and E-L and DNS models can serve the lab-scale studies. Besides the aforementioned interactions, the mesoscale structures that may form during the multi-phase PWG process can be modeled in the higher resolution reactor CFD frameworks to better satisfy the overlap between the micro-and macro-scale phenomena.

As can be inferred from the conclusions above, the recent increases in computational resources can be efficiently applied to improve the scalability of the CFD simulations and create more precise models of PWG at different scales. This can be done by using algorithms to heterogeneously perform different tasks on CPU and GPU clusters. This calls for an effective collaboration between different research areas, which can be better realized in the open-source programming community.

## Figures and Tables

**Table 1:** Examples of different methods to calculate the transport properties of a multi-component gas or liquid phase [92].

	Gas	Liquid
Viscosity	Reichenberg method Wilke method Herning and Zipperer approximation	Grunberg and Nissan method UNIFAC-VISCO method Teja and Rice method
Thermal Conductivity	Wassiljewa Equation Mason and Saxena Modification	Filippov Equation Jamieson correlation Baroncini correlation Rowley method Power Law method
Diffusion Coefficient	Stefan-Maxwell equation Blanc’s law	Perkins and Geankoplis correlation Takahashi et al. correlation Kooijman and Taylor correlation Kett and Anderson method

## Data Availability

Not applicable.

## Supplementary Materials

The following are available online at https://www.mdpi.com/article/10.3390/ma15124215/s1, general equations for the Eulerian-Eulerian and Eulerian-Lagrangian approaches (Equations (S1)–(S19)).

## Nomenclature

**Acronyms**	
CFB	Circulating Fluidized Bed
CGM	Coarse Grain Model
CMC	Continuous Multi-component
DAEM	Distributed Activation Energy Model
DFB	Dual Fluidized Bed reactor
DPM	Discrete Particle Method/Discrete Phase Method
DQMOM	Direct Quadrature Method of Moments
E-E	Eulerian-Eulerian approach
E-L	Eulerian-Lagrangian approach
ERN	Equivalent Reactor Network
FB	Fluidized Bed
FCMOM	Finite-size Domain Complete Set of Trial Functions Method of Moments
FTS	Fischer-Tropsch Synthesis
GL	Gas-Liquid
GS	Gas-Solid
KTGF	Kinetic Theory of Granular Flow
LBM	Lattice-Boltzmann Method
LK	Langmuir-Knudsen Model
MD	Molecular Dynamics
MOM	Method of Moments
MP-PIC	MultiPhase Particle-in-Cell
MSW	Municipal Solid Waste
OM	Order of Magnitude
PBE	Population Balance Equation
PR	Particle Resolved
PW	Plastic Waste
PWG	Plastic Waste Gasification
RDF	Refuse-Derived Fuel
SB	Spouted Bed
SCW	Super Critical Water
SRT	Solid Residence Time
**Roman and Greek Letters**	
A	Pre-exponential factor/Surface area
*Ar*	Aspect ratio of spheroids
Bi	Biot number
$c_{p}$	Specific heat capacity
Cou	Courant number
D	Diffusivity coefficient
Da	Damköhler number
*E*	Activation energy
$\vec{F}$	Force
$\vec{g}$	Gravity acceleration
h	Convective heat transfer coefficient
*H*	Enthalpy
k	Reaction kinetic constant
*L*	Latent heat/Characteristic length
m	Mass
*Ma*	Dimensionless Marangoni number
$n_{l}$	Number of droplets per mass of liquid
$n_{p}$	Number of particles
*Nu*	Nusselt number
p	Pressure
$P_{c}$	The perimeter of the circle equivalent to the maximum projection area of a particle
$P_{mp}$	Maximum projection perimeter
*Pr*	Prandtl number
*Py*	Pyrolysis number
q	Heat
$Q_{r}$	Generated or consumed heat due to reaction
r	Radius
*R*	Production rate/Universal gas constant
*Re*	Reynolds number
s	Solid-liquid interface position
$S_{h}$	Heat transfer between phases
$S_{u}$	Momentum transfer between phases
$S_{y}$	Species transfer between phases
$S_{\rho }$	Net mass transfer rate between phases
*SP*	Particle-based mass source term
*Sc*	Schmidt number
t	Time
*T*	Temperature
$u,\;\vec{u}$	Velocity
*V*	Volume
x	Spatial coordinate/Interface position
$X_{m}$	Monomer conversion
Y	Mass fraction
α	Volume fraction
ε	Porosity, void fraction
θ	Incident angle
λ	Thermal conductivity
μ	Dynamic viscosity
ν	Kinematic viscosity
ρ	Density
σ	Surface tension
$\overset{=}{\tau }$	Stress-strain tensor
ϕ	Sphericity parameter
χ	Circularity
ψ	Shape factor/Particle-based species transfer rate between phases
**Sub/Superscripts**	
0	Initial
I	First
II	Second
B	Bulk
c	Contact
cond	Conduction
conv	Convection
d	Drag
eff	Effective
f	Fluid
i	The i^th^ species
l	Liquid
p	Particle/Particle surface
pr	Pressure gradient
rad	Radiation
reac	Reaction
s	Solid
turb	Turbulent
