# Supplementary material for: Multi-Scale Modeling of Plastic Waste Gasification: Opportunities and Challenges
Source: Materials (Basel). 2022 Jun 14;15(12):4215. doi: 10.3390/ma15124215 (PMC9228121; doi:10.3390/ma15124215)
Supplement: Supplementary file 1 [file materials-15-04215-s001.zip › materials-1737669-supplementary.pdf]

Multi-scale Modeling of Plastic Waste Gasification: Opportunities and Challenges

Supplementary Information

Sepehr Madanikashani [1,2], Laurien A. Vandewalle [1], Steven De Meester [3], Juray De Wilde [2] and Kevin M. Van Geem [1,*]

[1] Laboratory for Chemical Technology (LCT), Faculty of Engineering and Architecture, Ghent University—Technologiepark 125, 9052 Ghent, Belgium; sepehr.madanikashani@ugent.be (S.M.); laurien.vandewalle@ugent.be (L.A.V.)
[2] Materials and Process Engineering (IMAP), Institute of Mechanics, Materials and Civil Engineering (iMMC), Université Catholique de Louvain—Place Sainte Barbe 2, 1348 Louvain-la-Neuve, Belgium; juray.dewilde@uclouvain.be
[3] Laboratory for Circular Process Engineering (LCPE), Faculty of Bioscience Engineering, Ghent University—Campus Kortrijk, Graaf Karel de Goedelaan 5, 8500 Kortrijk, Belgium; steven.demeester@ugent.be

* Correspondence: kevin.zangeem@ugent.be

1. General Equations for the Eulerian-Eulerian Approach

The general form of governing equations for the fluid and solid phase in the Eulerian-Eulerian framework is as follows.

The fluid phase continuity equation is:

$$\frac{\partial}{\partial t}\left(\alpha_f\rho_f\right) + \nabla\cdot\left(\alpha_f\rho_f\vec{u}_f\right) = S_{\rho,s\to f} \tag{S1}$$

where f and s indicate the fluid and solid phases, respectively, t is time, α is volume fraction, ρ is density, and $\vec{u}$ is velocity. S_ρ which is the net mass transfer rate between phases, in the case of PWG, could be the result of evaporation, cracking, or char production.

A similar expression can be written for the solid phase mass conservation equation because they are also considered as fluids:

$$\frac{\partial}{\partial t}\left(\alpha_s\rho_s\right) + \nabla\cdot\left(\alpha_s\rho_s\vec{u}_s\right) = S_{\rho,f\to s} \tag{S2}$$

One of the important transport phenomena in the reactor scale, especially for the fluidized bed technologies is the momentum transfer, formulated as the following equations for the fluid and solid phases, respectively:

$$\frac{\partial\left(\alpha_f\rho_f\vec{u}_f\right)}{\partial t} + \nabla\cdot\left(\alpha_f\rho_f\vec{u}_f\vec{u}_f\right) = -\alpha_f\nabla p + \nabla\cdot\bar{\bar{\tau}}_f + \alpha_f\rho_f\vec{g} + S_{u,s\to f} + \vec{F}_f \tag{S3}$$

$$\frac{\partial(\alpha_s \rho_s \vec{u}_s)}{\partial t} + \nabla \cdot (\alpha_s \rho_s \vec{u}_s \vec{u}_s) = -\alpha_s \nabla p - \nabla p_s + \nabla \cdot \bar{\bar{\tau}}_s + \alpha_s \rho_s \vec{g} + S_{u,f\to s} + \vec{F}_s \quad \text{(S4)}$$

In these equations, a few parameters are added compared to the continuity equations, including p which is pressure, $\bar{\bar{\tau}}$ which is the stress-strain tensor, $\vec{g}$ which is the gravity acceleration, and S_u which is momentum transfer between phases. $\vec{F}$, which is the other forces exerting on the phase, can encompass different forces such as drag, lift, virtual mass, etc. The stress-strain tensor is defined as the following equations:

$$\bar{\bar{\tau}}_f = \alpha_f \rho_f \nu_{\text{eff},f} \left(\nabla \vec{u}_f + \nabla \vec{u}_f^T\right) - \frac{2}{3} \alpha_f \rho_f \nu_{\text{eff},f} \left(\nabla \cdot \vec{u}_f\right) \bar{\bar{I}} \quad \text{(S5)}$$

$$\bar{\bar{\tau}}_s = \rho_s \nu_{\text{eff},s} (\nabla \vec{u}_s + \nabla \vec{u}_s^T) + \rho_s \left(\nu_{B,s} - \frac{2}{3} \nu_{\text{eff},s}\right) (\nabla \cdot \vec{u}_s) \bar{\bar{I}} \quad \text{(S6)}$$

where ν_{eff} is the effective kinematic viscosity which is the sum of molecular viscosity and turbulent viscosity and $\nu_{B,s}$ is the solid bulk viscosity.

The energy equation for the fluid phase (temperature-based):

$$\frac{\partial\left(\alpha_f \rho_f c_{p,f}\right)}{\partial t} + \nabla \cdot \left(\alpha_f \rho_f \vec{u}_f c_{p,f}\right) = \alpha_f \nabla\left(\lambda_{eff,f} \nabla T_f\right) + S_{h,s\to f} + Q_{r,f} \quad \text{(S7)}$$

where c_p is specific heat capacity, λ is thermal conductivity, T is temperature, and S_h is heat transfer between phases, and Q_r is the generated or consumed heat due to reaction. Similar to the effective viscosity, the effective thermal conductivity is obtained from the molecular and turbulent terms, according to the following equation:

$$\lambda_{eff,f} = \lambda_f + \rho_f c_{p,f} \frac{\nu_{turb,f}}{Pr_{turb,f}} \quad \text{(S8)}$$

where Pr_{turb} is the turbulent Prandtl number.

Similarly, the energy equation for the solid phase is expressed as:

$$\frac{\partial\left(\alpha_s \rho_s c_{p,s}\right)}{\partial t} + \nabla \cdot \left(\alpha_s \rho_s \vec{u}_s c_{p,s}\right) = \alpha_s \nabla\left(\lambda_{eff,s} \nabla T_s\right) + S_{h,f\to s} + Q_{r,s} \quad \text{(S9)}$$

where $\lambda_{eff,s}$ is formulated as:

$$\lambda_{eff,s} = \rho_s c_{p,s} \frac{\nu_{eff,s}}{Pr_{eff,s}} \quad \text{(S10)}$$

The species transport equation in the fluid phase is formulated as:

$$\frac{\partial\left(\alpha_f \rho_f Y_{i,f}\right)}{\partial t} + \nabla \cdot \left(\alpha_f \rho_f \vec{u}_f Y_{i,f}\right) = \nabla \cdot \left(\alpha_f \rho_f D_{eff,i,f} \nabla Y_{i,f}\right) + \alpha_f R_{i,f} + S_{y_i,s\to f} \quad \text{(S11)}$$

where Y is the species mass fraction, R is the net production rate, and S_y indicates the species transfer between phases. The effective diffusivity consists of molecular and turbulent diffusivities, as:

$$D_{eff,i,f} = D_{i,f} + \frac{\nu_{turb,f}}{Sc_{turb,f}} \tag{S12}$$

where Sc_{turb} is the turbulent Schmidt number. For the solid phase, the species transport is expressed as:

$$\frac{\partial(\alpha_s \rho_s Y_{i,s})}{\partial t} + \nabla \cdot (\alpha_s \rho_s \vec{u}_s Y_{i,s}) = \alpha_s R_{i,s} + S_{y_i,f\to s} \tag{S13}$$

Note that the diffusion term is omitted in equation S13 since transport of the species in the solid phase is assumed to be solely due to convective transport of the solid-phase particles.

It is worth mentioning that the equations above are written to transfer between fluid and solid phases. Hence, in the case of considering more than two phases, transfer terms that appear in these equations should be expressed as the sum of the transfers of binary phases.

2. General Equations for the Eulerian-Lagrangian Approach

The following equations demonstrate the general expression for the governing equations in the discrete phase in the Eulerian-Lagrangian framework.

The particle phase mass balance equation is:

$$\frac{dm_s}{dt} = SP_{\rho,f\to s} \tag{S14}$$

where m is the particle mass and SP is the particle-based version of the source terms that appear in equations S1 and S2. In the Eulerian-Eulerian framework, the equations are cell-based, while in the Lagrangian, they are particle-based. Hence, in coupling the equations in the E-L approach, this should be taken into account. This way, the mass source term, $S_{\rho,s\to f}$, which appears in equation S1, is defined as [1]:

$$S_{\rho,s\to f} = \sum_{i=1}^{n_p} SP_{\rho_i,s\to f} \tag{S15}$$

where n_p is the number of particles in the fluid cell.

If the pyrolysis and devolatilization reaction is considered as a single-step process with its defined kinetic parameters, the mass evolution is expressed as the following equation [1]:

$$\frac{dm_s}{dt} = A\, exp\left(-\frac{E}{RT}\right) m_s \tag{S16}$$

where A is the pre-exponential factor, E is the activation energy, and R is the universal gas constant. It is worth mentioning that different models could be implemented in defining the mass balance of the particles. As an example, for the char gasification reaction, different models coupled to the mass transfer limits can be applied, which is demonstrated by Xie et al. [1]

The particle phase momentum equation is:

$$m_s \frac{d\vec{u}_s}{dt} = m_s\vec{g} + \vec{F}_d + \vec{F}_c + \vec{F}_{pr} \tag{S17}$$

where $\vec{g}$ is gravity acceleration and $\vec{F}_d$, $\vec{F}_c$, and $\vec{F}_{pr}$ are the drag, contact, and pressure gradient forces.

The energy balance for the particle is:

$$m_s\, c_{p,s} \frac{dT_s}{dt} = q_{cond} + q_{conv} + q_{rad} + q_{reac} \tag{S18}$$

where c_p is the specific heat capacity, T is temperature, and q_{cond}, q_{conv}, and q_{rad} are conductive, convective, and radiative heat transfer, while q_{reac} is the heat released/absorbed by the reaction.

The species balance equation in the particle phase is:

$$\frac{dm_sY_{i,s}}{dt} = R_{i,s} + \psi_{i,f\rightarrow s} \tag{S19}$$

where Y is the species mass fraction, R is the net production rate of species, and ψ is the particle-based species transfer.

References

[1] Xie J, Zhong W, Shao Y, Li K. Coupling of CFD-DEM and reaction model for 3D fluidized beds. Powder Technol 2019;353:72-83.
